# It’s about time: A synthesis of changing phenology in the Gulf of Maine ecosystem

**DOI:** 10.1111/fog.12429

**Published:** 2019-04-22

**Authors:** Michelle D. Staudinger, Katherine E. Mills, Karen Stamieszkin, Nicholas R. Record, Christine A. Hudak, Andrew Allyn, Antony Diamond, Kevin D. Friedland, Walt Golet, Meghan Elisabeth Henderson, Christina M. Hernandez, Thomas G. Huntington, Rubao Ji, Catherine L. Johnson, David Samuel Johnson, Adrian Jordaan, John Kocik, Yun Li, Matthew Liebman, Owen C. Nichols, Daniel Pendleton, R. Anne Richards, Thomas Robben, Andrew C. Thomas, Harvey J. Walsh, Keenan Yakola

**Affiliations:** ^1^ Department of the Interior Northeast Climate Adaptation Science Center Amherst Massachusetts; ^2^ Department of Environmental Conservation University of Massachusetts Amherst Amherst Massachusetts; ^3^ Gulf of Maine Research Institute Portland Maine; ^4^ Bigelow Laboratory for Ocean Sciences East Boothbay Maine; ^5^ Department of Ecology, Center for Coastal Studies Provincetown Massachusetts; ^6^ University of New Brunswick Fredericton New Brunswick Canada; ^7^ NOAA, Northeast Fisheries Science Center, National Marine Fisheries Service Narragansett Rhode Island; ^8^ School of Marine Sciences University of Maine Orono Maine; ^9^ School of Marine and Atmospheric Sciences Stony Brook University Stony Brook, New York; ^10^ Department of Biology Woods Hole Oceanographic Institution Woods Hole Massachusetts; ^11^ USGS New England Water Science Center, U.S. Geological Survey Augusta Maine; ^12^ Fisheries and Oceans Canada, Bedford Institute of Oceanography Dartmouth Nova Scotia Canada; ^13^ Virginia Institute of Marine Science College of William and Mary Gloucester Point Virginia; ^14^ NOAA, Northeast Fisheries Science Center, National Marine Fisheries Service Orono Maine; ^15^ College of Marine Science University of South Florida St. Petersburg Florida; ^16^ Office of Ecosystem Protection, US EPA New England Boston Massachusetts; ^17^ Anderson Cabot Center for Ocean Life, New England Aquarium, Central Wharf Boston Massachusetts; ^18^ Population Dynamics Branch NOAA Northeast Fisheries Science Center Woods Hole Massachusetts; ^19^ Connecticut Ornithological Association Fairfield Connecticut

**Keywords:** coastal, fish, Gulf of Maine, life cycle, marine, marine invertebrates, marine mammals, migration, phenology, phytoplankton, seabirds, seasonal, timing, zooplankton

## Abstract

The timing of recurring biological and seasonal environmental events is changing on a global scale relative to temperature and other climate drivers. This study considers the Gulf of Maine ecosystem, a region of high social and ecological importance in the Northwest Atlantic Ocean and synthesizes current knowledge of (a) key seasonal processes, patterns, and events; (b) direct evidence for shifts in timing; (c) implications of phenological responses for linked ecological‐human systems; and (d) potential phenology‐focused adaptation strategies and actions. Twenty studies demonstrated shifts in timing of regional marine organisms and seasonal environmental events. The most common response was earlier timing, observed in spring onset, spring and winter hydrology, zooplankton abundance, occurrence of several larval fishes, and diadromous fish migrations. Later timing was documented for fall onset, reproduction and fledging in Atlantic puffins, spring and fall phytoplankton blooms, and occurrence of additional larval fishes. Changes in event duration generally increased and were detected in zooplankton peak abundance, early life history periods of macro‐invertebrates, and lobster fishery landings. Reduced duration was observed in winter–spring ice‐affected stream flows. Two studies projected phenological changes, both finding diapause duration would decrease in zooplankton under future climate scenarios. Phenological responses were species‐specific and varied depending on the environmental driver, spatial, and temporal scales evaluated. Overall, a wide range of baseline phenology and relevant modeling studies exist, yet surprisingly few document long‐term shifts. Results reveal a need for increased emphasis on phenological shifts in the Gulf of Maine and identify opportunities for future research and consideration of phenological changes in adaptation efforts.

## 1 INTRODUCTION

Changes in phenology, or the seasonal timing of recurring events, have emerged as a primary indicator of species responses to climate change (Parmesan, 2006; Parmesan & Yohe, 2003). In terrestrial ecosystems, earlier onset of spring and advances in the timing of emergence, flowering, and arrival times of migratory organisms have been well documented (Dunnell & Travers, 2011; Inouye, 2008; Miller‐Rushing, Inouye, & Primack, 2008; Miller‐Rushing, Lloyd‐Evans, Primack, & Satzinger, 2008; Post, Pedersen, Wilmers, & Forchhammer, 2008; Wiebe & Gerstmar, 2010). However, far fewer examples exist that provide direct evidence for climate‐induced shifts in marine phenology (but see Edwards & Richardson, 2004). A recent evaluation of global marine responses to climate change found that temporal shifts represented only a small percentage of all observations, and were exhibited primarily by phytoplankton, zooplankton, and seabirds; in contrast, observations of changes in abundance and spatial distribution were more widespread (Poloczanska et al., 2013).

Datasets suitable for evaluating shifts in marine phenology exist but are often difficult to access and remain underutilized (Thomas, Fornwall, Weltzin, & Griffis, 2014). Where evaluations of marine phenology have been explored, rates of advancement are faster than in terrestrial systems (Poloczanska et al., 2013). Rapid and differential shifts among dependent species (e.g., predator–prey) and across trophic levels increase the potential for asynchronies and mismatches in food and other resources, thereby leading to negative impacts on individual fitness, population dynamics, and ecosystem function (Doney et al., 2012; Durant et al., 2013; Staudinger et al., 2013). In addition, mismatches with human uses and management tools may occur, resulting in disruptions to fisheries and other ecosystem services, thus impeding resource use, conservation, and management (Mills et al., 2013; Peer & Miller, 2014).

Marine ecological structure and dynamics in the Northwest Atlantic Ocean are highly seasonal (Liu et al., 2005). In particular, the Gulf of Maine (GoM) supports a range of seasonal migrants whose arrival and/or reproduction timing coincides with preferred temperature regimes and peaks in forage resources that provide energy for spawning, support recruitment of early life stages, and fuel long‐distance movements to other regions. Highly migratory species are especially at risk to mismatches with environmental and ecological resources because the conditions in departure and destination habitats may be shifting at different rates (Wood & Kellermann, 2015). For example, long‐distance migrants such as baleen whales, seabirds, and large pelagic fish use the GoM as an intermediate temporal habitat between trips across the Atlantic Ocean and to Arctic and Antarctic ecosystems; all of these regions are exhibiting unprecedented rates of environmental change (Johannessen et al., 2004; Kaufman et al., 2009; Pershing et al., 2015). A diversity of other regional taxa (e.g., diadromous fishes) undertake shorter seasonal and ontogenetic movements between inland (e.g., streams, rivers, and coastal ponds), nearshore (e.g., estuaries and bays), and offshore habitats to forage, spawn or follow preferred habitat conditions.

The GoM and broader U.S. Northeast Shelf are experiencing rapid and intense changes in bottom, air, and sea surface temperatures (SST) during all seasons (Kleisner et al., 2016; Pershing et al., 2015; Shearman & Lentz, 2010; Thomas et al., 2017), and relative to other global oceans, with the most pronounced warming occurring in recent decades (Friedland & Hare, 2007; Pershing et al., 2015). Future projections of seasonal bottom and SST over the coming century indicate the GoM is likely to continue to be a hotspot of warming with rates 2–3 times faster than other global oceans (Saba et al., 2016). Across the region, there is already strong evidence of geographic range shifts in many commercially important fish stocks (Kleisner et al., 2017; Nye, Link, Hare, & Overholtz, 2009; Pinsky, Worm, Fogarty, Sarmiento, & Levin, 2013) as well as major changes in community composition, dominance, and structure (Collie, Wood, & Jeffries, 2008; Dijkstra, Westerman, & Harris, 2011; Griffis & Howard, 2013; Howell & Auster, 2012; Wood, Collie, & Hare, 2009). Nonetheless, a significant gap remains in our understanding of how regional populations, species, and communities are responding to climate impacts through changes in timing of recurring life events. To address this need, the present study focuses on the GoM and aims to: (a) characterize key seasonal environmental and ecological processes, patterns, and events; (b) provide a comprehensive synthesis of evidence for shifts in phenology; (c) evaluate the socio‐ecological implications of regional shifts in phenology; and (d) provide a set of recommendations for phenology‐focused adaptation strategies and actions. The highly seasonal nature of the GoM, coupled with substantial regional warming, makes it a compelling model system for evaluating phenological shifts, identifying related impacts, and developing insights into expected changes in the coming decades for global marine ecosystems and the services they provide to humans.

## 2 KEY ENVIRONMENTAL FEATURES AND PHENOLOGICAL PATTERNS IN THE GoM

**Figure 1:**
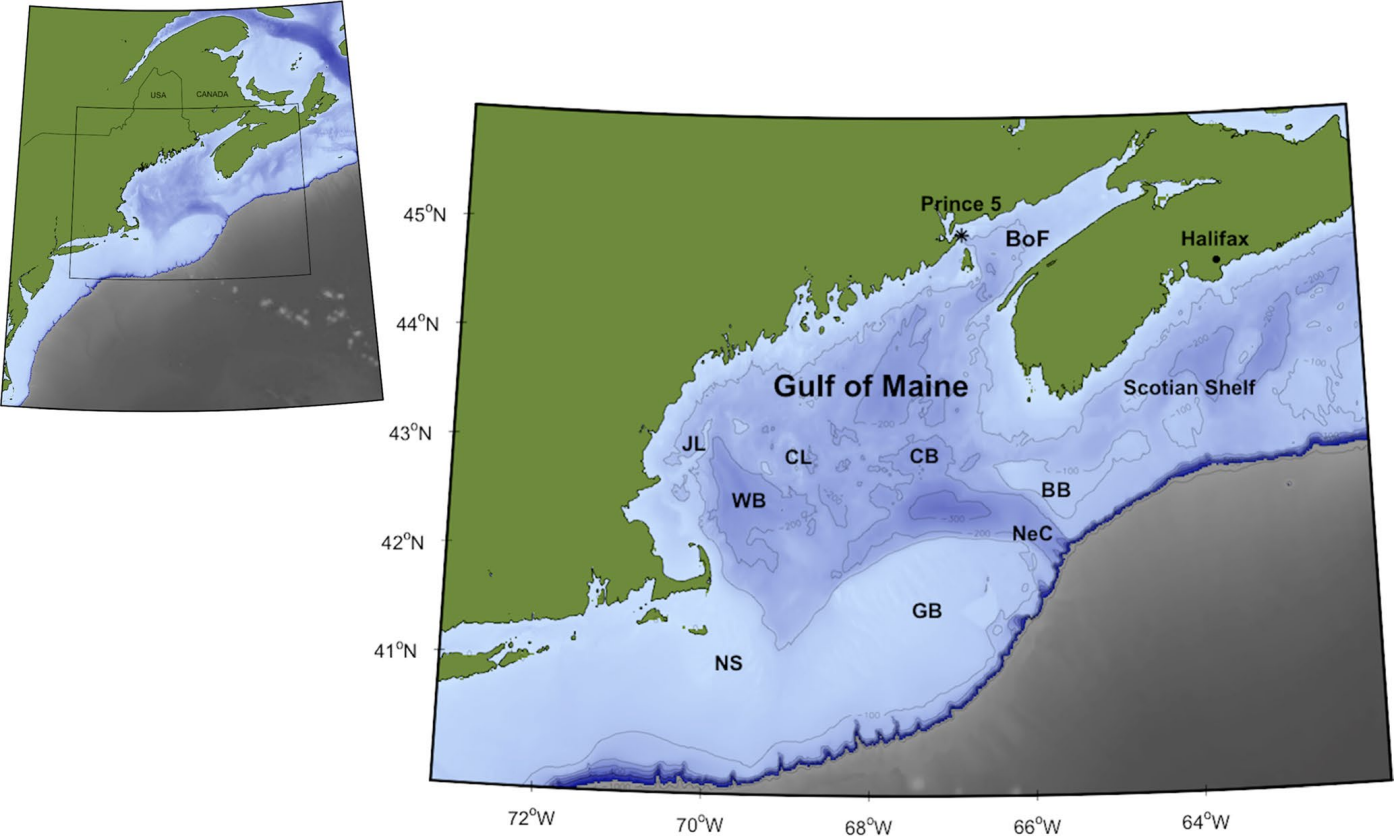
We define the Gulf of Maine as including the Bay of Fundy (BoF), Georges Bank (GB), the western Scotian Shelf, and the neighboring slope sea according to boundaries used in Johnson et al. (2011). JL = Jeffreys Ledge, CL = Cashes Ledge, CB = Crowell Basin, BB = Brown Banks, WB = Wilkinson Basin, NeC = Northeast Channel, and NS = Nantucket Shoals [Colour figure can be viewed at wileyonlinelibrary.com]

**Figure 2:**
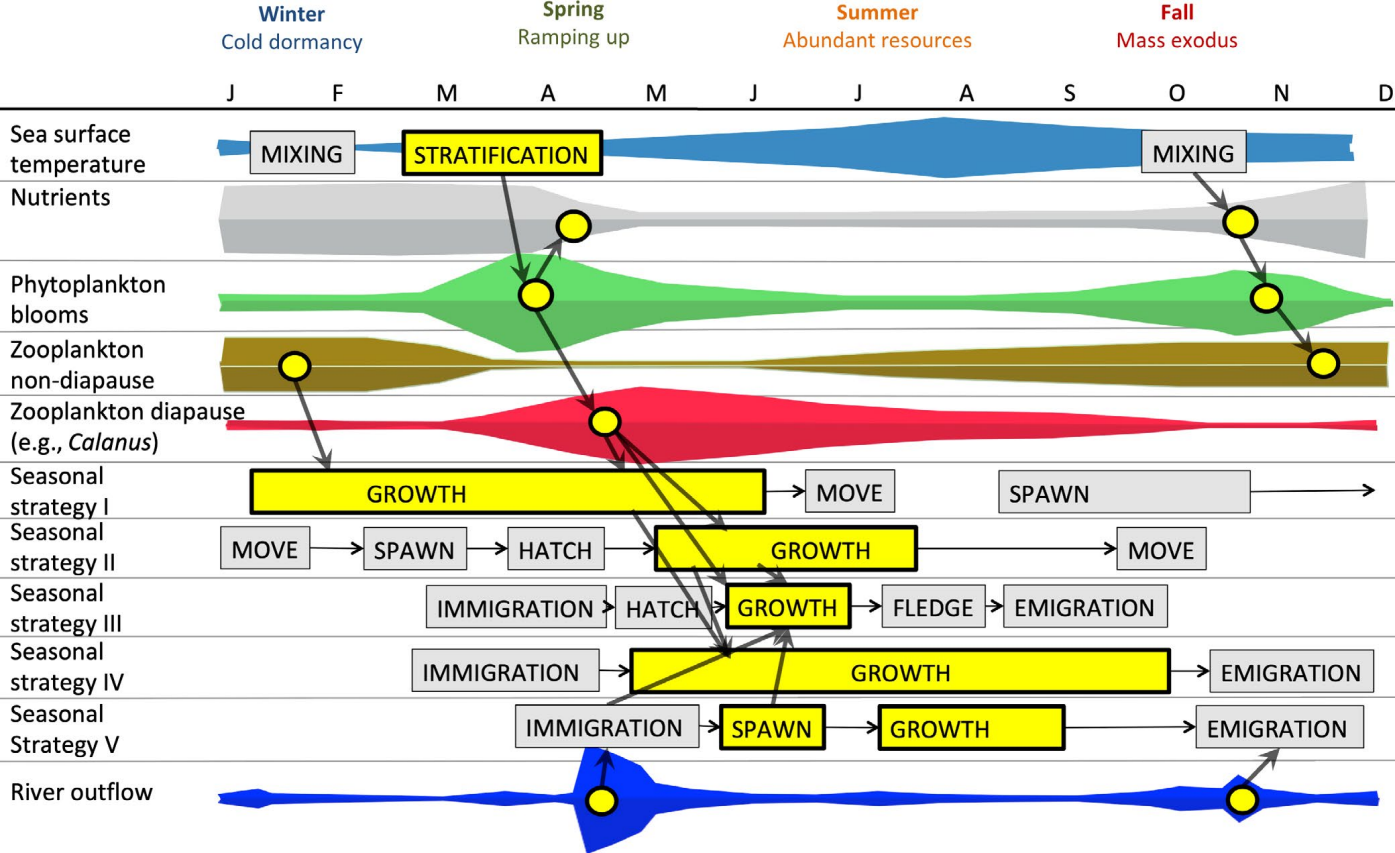
Idealized model of phenological events and linkages in the Gulf of Maine (GoM). Yellow highlighted bars, circles, and arrows indicate match points where one phenological event relies on another. Width of yellow and gray colored bars indicates duration of events. Phytoplankton blooms, zooplankton diapause strategy (specific to *Calanus* copepods), non‐diapause strategy (other zooplankton including euphausiids), and river outflow event timing are depicted based on magnitude measurements. Strategy I (fall spawn, spring growth (e.g., Atlantic herring), Strategy II (spring spawn, summer growth (e.g., Atlantic cod), Strategy III (migratory seabirds), Strategy IV (large pelagics), and Strategy V (anadromous fish) are depicted by a sequence of events with different processes and/or life stages. Note not all possible strategies present in the GoM are shown in figure [Colour figure can be viewed at wileyonlinelibrary.com]

Understanding how species and habitats are responding to climate change requires a firm understanding of baseline characteristics and cycles. As of 2016, there were approximately 3,300 documented species in the GoM (~2,600 fauna and ~700 flora), with new species arriving or still being discovered (Fautin et al., 2010; Gulf of Maine Census, 2016; Johnson et al., 2011). We follow the defined boundaries for the GoM (Figure 1) reported previously in Johnson et al. (2011) and focus on a subset of species that exhibit strong seasonality, have a definitive connection to coastal and marine habitats during a portion of their life history, and are known to provide valued ecosystem services within the region. The following section presents a synthesis of best available information that identifies key seasonal coastal and oceanographic processes linked to the phenology of major functional groups in the GoM (Figure 2). Examples obtained from primary scientific literature (e.g., year day(s), species‐specific events, and locations) on the timing and occurrence of phenological events are listed in Supporting Information Appendix S1.

### 2.1 Spring: ramping up

The spring phytoplankton bloom is a fundamental event in the GoM and greater North Atlantic that stimulates secondary production and ultimately supports a large biomass of marine fauna through seasonal peaks in forage resources that fuel growth, reproduction, and migrations. Spring bloom initiation progresses from east on the Scotian Shelf to west in the GoM (Ji et al., 2007; Platt et al., 2010; Song, Ji, Stock, & Wang, 2010). The timing of bloom initiation varies interannually across the region due to fluctuations in a suite of complex drivers. At a broad scale, differences in sea surface salinity (Ji et al., 2007; Song et al., 2010) and/or irradiance in the surface mixed layer (Ji, Edwards, Mackas, Runge, & Thomas, 2010; Platt et al., 2010; Townsend, Cammen, Holligan, Campbell, & Pettigrew, 1994; Townsend & Spinrad, 1986; Zhai, Platt, Tang, Sathyendranath, & Hernández Walls, 2011) appear to influence bloom variability. Coastal and other tidally mixed regions such as the Bay of Fundy and Georges Bank sustain some phytoplankton production throughout the year (Thomas, Townsend, & Weatherbee, 2003). Springtime productivity along the coast progresses latitudinally from south to north and can differ in timing and magnitude from offshore habitats as a function of temperature, tidal mixing, turbidity, and light availability (Hunter‐Cevera et al., 2016; Tian et al., 2015; Townsend, 1984; Townsend & Spinrad, 1986). O'Reilly and Zetlin (1998) estimated spring bloom duration from in situ samples taken between 1977 and 1988, and found it started as early as February throughout most of the GoM and lasted as late as April in the Great South Channel, Wilkinson and Jordan Basins, and on the Scotian Shelf. In another study conducted between 1998 and 2008, satellite‐derived surface chlorophyll‐a measurements suggested the GoM spring bloom typically lasted from March to June and was primarily influenced by nutrient concentrations (Song et al., 2010).

**Figure 3:**
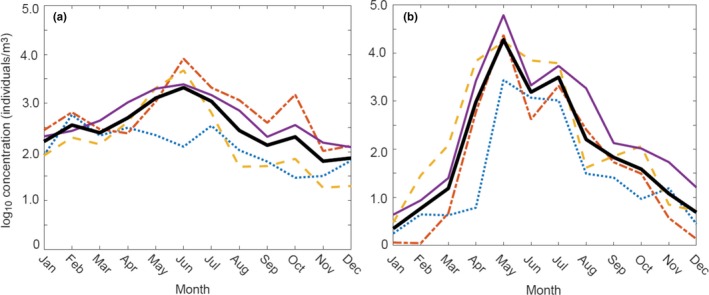
Monthly peaks in the seasonal timing of abundance of (a) late stage (CV) and adult *Calanus finmarchicus*, and (b) juvenile (CI‐CIV) *Calanus* spp. during four historical time periods 1961–1974 (blue dotted line), 1978–1988 (red dot‐dash line), 1992–2000 (yellow dashed line), 2001–2013 (thin solid purple line), and the mean of all years (thick solid black line). Data are from the Gulf of Maine Continuous Plankton Recorder (CPR) dataset, and analysis is after Pershing et al., 2005. See Supporting Information Appendix S2 for additional details [Colour figure can be viewed at wileyonlinelibrary.com]

Historical peaks (1978–2006) in the spring phytoplankton bloom in April immediately precede peak zooplankton abundances in May (Kane, 2009, 2011). During annual increases in secondary production in the central GoM and Scotian Shelf, *Calanus finmarchicus* emerges from overwintering diapause and dominates the zooplankton biomass (Durbin, Gilman, Campbell, & Durbin, 1995; Johnson, Casault, Head, & Spry, 2016; Kane, 1993; Manning & Bucklin, 2005; Runge et al., 2015). Due to its high lipid content, *C. finmarchicus* is a key prey species linked directly and indirectly to seasonal energy accumulation and growth of higher trophic species such as forage and commercial fishes, baleen whales, and seabirds (Goyert, 2014; Nelson & Ross, 1991; Payne, Wiley, Pittman, Clapham, & Jossi, 1990; Pendleton et al., 2009; Richardson, Palmer, & Smith, 2014). While the timing of emergence is variable (Johnson et al., 2008; Maps et al., 2012), mean monthly peaks occurred on average in June for adult and late‐stage *C. finmarchicus,* and in May for juvenile *Calanus* spp. (primarily *C. finmarchicus*) between 1961 and 2013 in the central Gulf (Figure 3; Supporting Information Appendix S2; also see Pershing et al., 2005).

**Figure 4:**
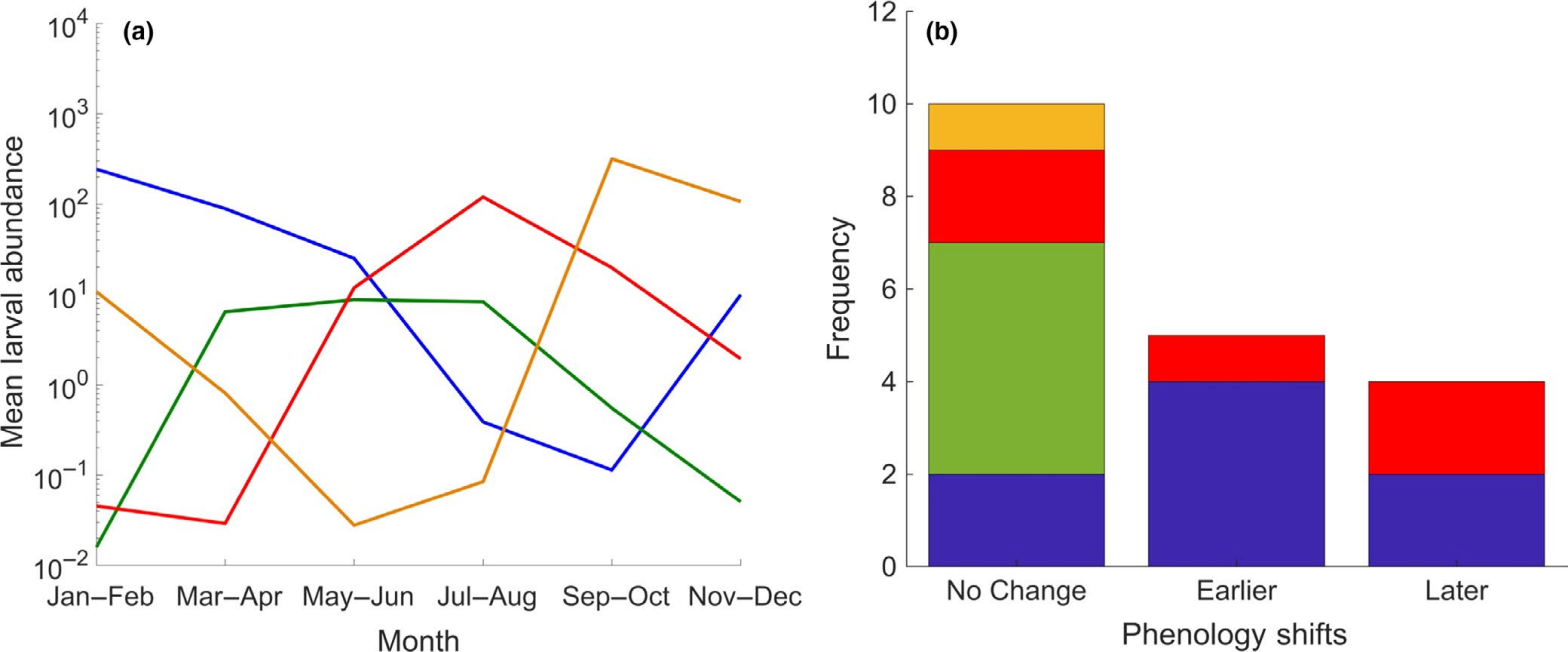
(a) Mean larval abundance (number 10 m^−2^) and (b) phenological shifts in occurrence of 19 ichthyoplankton taxa occurring in Georges Bank and Gulf of Maine regions as reported from Walsh et al. (2015). Larvae were classified into four seasons: winter (blue, 8 taxa), spring (green, 5 taxa), summer (red, 5 taxa), and fall (orange, 1 taxa) based on the three‐highest ranked bi‐monthly occurrences and total abundance was average for each season. Changes in phenology are based on a comparison between two time periods (1977–1987 to 1999–2008) for each taxon [Colour figure can be viewed at wileyonlinelibrary.com]

In coastal waters of the central and western GoM, as well as the outer Bay of Fundy, meroplankton reach peak abundances in spring; this includes planktonic phases of benthic species such as *Balanus *spp. (barnacles) and northern shrimp (*Pandalus borealis*), which begin settling out of the water column approximately in June (Haynes & Wigley, 1969; Johnson et al., 2016; Johnson, Curtis, Pepin, & Runge, 2010; Richards, 2012; Townsend, 1983). Meroplankton also includes ichthyoplankton, which develop later into free‐swimming juveniles (Townsend, 1983; Walsh, Richardson, Marancik, & Hare, 2015). Routine ichthyoplankton surveys of the entire U.S. Northeast shelf identified 45 abundant demersal and pelagic taxa, of which 19 commonly occur in the GoM and Georges Bank (Walsh et al., 2015). Some demersal fishes such as redfishes (*Sebastes* spp.) and flatfishes (e.g., witch flounder (*Glyptocephalus cynoglossus*), American plaice (*Hippoglossoides platessoides*)) increase in occurrence in March–April, maintaining high abundance through the summer (Figure 4a).

Spring is a critical time for many diadromous fish species, which undergo annual inshore movements from the marine environment to coastal freshwater ponds, rivers, and streams to spawn (Saunders, Hachey, & Fay, 2006). Migration timing in alewives (*Alosa pseudoharengus*), blueback herring (*A. aestivalis*), American shad (*A. sapidissima*) Atlantic sturgeon (*Acipenser oxyrinchus oxyrinchus*), and rainbow smelt (*Osmerus mordax*) coincides with changes in spring water temperatures, river flow, and timing of ice breakup (Collette & Klein‐MacPhee, 2002; Melnychuk, Dunton, Jordaan, McKown, & Frisk, 2017; Rupp, 1959). Arrival of diadromous fishes to spawning systems varies with latitude; alewives are the first to arrive, typically in early to mid‐April in more southern systems (e.g., Parker River Estuary, MA: Alexander et al., 2017; Ellis & Vokoun, 2009), and early June in northern systems (e.g., Penobscot River, ME: Huntington, Hodgkins, & Dudley, 2003). Blueback herring and American shad runs start in late April and peak during May and June (Alexander et al., 2017; Collette & Klein‐MacPhee, 2002; Saunders et al., 2006). The spring run of adult Atlantic salmon (*Salmo salar*) to natal streams historically began as early as April but peak activity occurs in June, just a month later than the out‐migration of transitioning smolts, which emigrate in May and June (Meister, 1962; Saunders et al., 2006). As adult anadromous species move inshore and inland to spawn, young catadromous American eels (*Anguilla rostrata*) also migrate as elvers into brackish waters and ascend coastal rivers between late March and June (Collette & Klein‐MacPhee, 2002; Facey & Van Den Avyle, 1987).

Many benthic and pelagic species that inhabit the continental shelf such as American lobster (*Homarus americanus*), Atlantic mackerel (*Scomber scombrus*), and longfin squid (*Doryteuthis pealeii*) move from their deeper offshore overwintering grounds in late spring as water temperatures and prey concentrations rise in shallow coastal areas (Black, Rowell, & Dawe, 1987; Collette & Klein‐MacPhee, 2002; Cooper & Uzmann, 1971; Ennis, 1984; Lange & Sissenwine, 1980; Methratta & Link, 2007; Mills et al., 2013). Conversely, spiny dogfish (*Squalus acanthias*) migrate offshore to deeper, relatively warmer waters for parturition (Methratta & Link, 2007; Sagarese et al., 2014).

Spring marks the time of arrival and peak abundance for a number of highly migratory mammalian and avian species of conservation concern in GoM habitats. Planktivorous North Atlantic right whales (*Eubalaena glacialis*) begin to appear in Cape Cod Bay as early as January, with the total number of whales peaking in March or April as they take advantage of zooplankton blooms (Hamilton & Mayo, 1990; Mayo & Marx, 1990; Pendleton et al., 2009; Schevill, Watkins, & Moore, 1986; Winn, Price, & Sorensen, 1986). Although fin whales (*Balaenoptera physalus*) are seen year‐round, they show strong site fidelity in the GoM, with an influx in the spring and peak occupancy in the summer (Agler, Schooley, Frohock, Katona, & Seipt, 1993; CETAP, 1982). The majority of the GoM humpback whale stock (*Megaptera novaeangliae*) arrives during the spring and summer after traveling thousands of miles from their breeding grounds in the West Indies (Clapham & Mayo, 1987; Katona & Beard, 1990; Kenney, Scott, Thompson, & Winn, 1997; Stevick et al., 2006). Smaller cetaceans such as white‐sided dolphins (*Lagenorhynchus acutus*) and the harbor porpoise (*Phocena phocoena*) also peak in abundance in spring (Kenney et al., 1997). Many of these piscivorous marine mammals as well as highly migratory seabirds migrate into the region in time to take advantage of peaks in abundance of prey such as sand lance (*Ammodytes* spp.) and Atlantic herring (*Clupea harengus*; Hatch, 2002; Payne et al., 1990; Robards, Willson, Armstrong, & Piatt, 2000; Tupper, Anthony, Chenoweth, & MacCluen, 1998).

**Figure 5:**
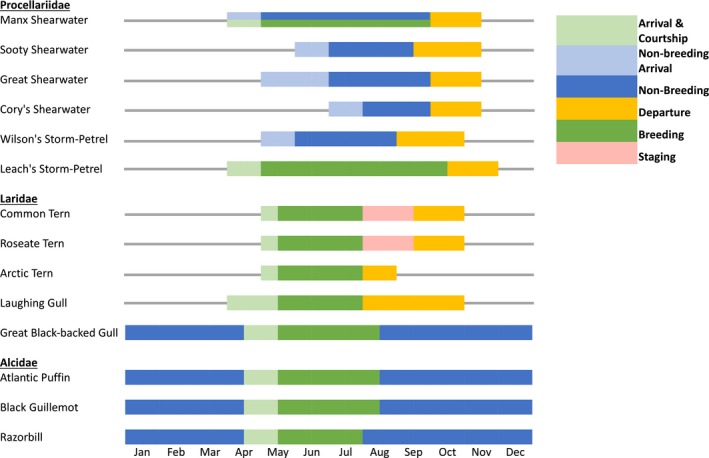
The seasonal phenology of selected migratory seabirds in the Gulf of Maine. Colors depict arrival and courtship (light green), breeding (dark green), non‐breeding arrival (light blue), non‐breeding (dark blue), staging (pink), and departure (orange). Procellariids include: Manx Shearwater (*Puffinus puffinus*), Sooty Shearwater (*Ardenna grisea*), Great Shearwater (*A. gravis*), Cory's Shearwater (*Calonectris diomedea*), Wilson's Storm‐Petrel (*Oceanites oceanicus*) and Leach's Storm‐Petrel (*Oceanodroma leucorhoa*). Larirds include: Common Tern (*Sterna hirundo*), Roseate Tern (*S. dougallii*), Arctic Tern (*S. paradisaea*), Laughing Gull (*Leucophaeus atricilla*), and Great Black‐backed Gull (*Larus marinus*). Alcids include: Atlantic Puffin (*Fratercula arctica*), Black Guillemot (*Cepphus grylle*), and Razorbill (*Alca torda*). Data were adapted from Petrels, Albatrosses & Storm‐Petrels of North America (Howell, 2012) and unpublished data from National Audubon Society Seabird Restoration Program [Colour figure can be viewed at wileyonlinelibrary.com]

The diversity of the marine bird community in the GoM increases in spring as both breeding and non‐breeding species return to the region. Migratory breeding species, such as terns (*Sterna* spp.), alcids (e.g., Atlantic Puffins, *Fratercula arctica*), and Leach's storm‐petrels (*Oceanodroma leucorhoa*), typically arrive at colonies in April or early May (Figure 5). Species breeding at inshore and western colonies usually arrive earlier compared to eastern and offshore colonies, and breeding begins a few weeks to a month later (Howell, 2012). Non‐breeding immature and adult seabird species such as great shearwaters (*Ardenna gravis*) are believed to arrive on Georges Bank in late May and early June to forage during the austral winter (Overholtz & Link, 2007; Powers, 1983; Powers & Backus, 1987). During March and April, offshore spring plankton blooms draw large numbers of gulls and other birds to engage in energetic “plankton‐feeding” behavior (Vermeer, Szabo, & Greisman, 1987).

### 2.2 Summer: an abundance of resources

Peak SSTs and strong stratification of the water column are key oceanographic features of summertime in the GoM. These features result in surface nutrient limitation and phytoplankton biomass levels over deeper basins that can approach levels comparable with the annual winter minimum. In the mid‐coastal region, subsurface chlorophyll maxima occur at approximately 20 m and are dominated by flagellates (Holligan, Balch, & Yentsch, 1984; O'Reilly & Zetlin, 1998; Thomas et al., 2003).

Warmer waters and abundant prey spurred by the spring primary and secondary blooms support high biodiversity during summer. In the central and southern GoM, as well as on Georges Bank, there is a succession of small copepod species including *Centropages typicus*, *Pseudocalanus* spp., and *Metridia lucens *(Pendleton et al., 2009; Record, O'Brien, Stamieszkin, & Runge, 2016). Total copepod abundance in the central GoM remains high from spring through summer (Kane, 2009) and biomass peaks in coastal waters of the western GoM during late July to early August (Manning & Bucklin, 2005). In contrast to much of the GoM, total zooplankton biomass and abundance in the outer Bay of Fundy is typically highest in July to September (Johnson et al., 2016), with overall copepod diversity peaking in September (Johnson et al., 2016; Record, Pershing, & Jossi, 2010).

Ichthyoplankton of some demersal (e.g., fourbeard rockling (*Enchelyopus cimbrius*)) and pelagic species (e.g., hakes (*Merluccius albidus*, *M. bilinearis*) and Atlantic mackerel (*Scomber scombrus*)) reach peak abundances during July to August (Figure 4a; Walsh et al., 2015). Commercially important flounders (Paralichthyidae, Pleuronectidae) and other groundfish utilize inshore habitats as summer nursery grounds (Collette & Klein‐MacPhee, 2002). In addition, a number of larval and juvenile warm water species (GSO, 2016: http://www.gsoproject.org/species-identification/) occur in the GoM during summer after being transported north via the Gulf Stream Current and carried inshore in warm‐core rings (Hare et al., 2002; Wood et al., 2009).

Peak concentrations of forage species (e.g., longfin and shortfin squids (*Illex illecebrosus*)) attract recreationally and commercially important fishes such as bluefish (*Pomatomous saltatrix*) and striped bass (*Morone saxatilis*), which migrate north from overwintering grounds in the south and mid‐Atlantic regions starting in late May to early June (Collette & Klein‐MacPhee, 2002; Wuenschel et al., 2012). Highly migratory large pelagic species including bluefin tuna (*Thunnus thynnus*), basking sharks (*Cetorhinus maximus*), small‐ to medium‐sized cetaceans (e.g., long‐finned pilot whales (*Globicephala melas*); Risso's dolphins (*Grampus griseus*)), and great whales (e.g., North Atlantic right whale)) also utilize offshore waters of the GoM as summer foraging grounds (Campana et al., 2008; Curtis, Zeeman, Summers, Cadrin, & Skomal, 2014; Diamond, 2012; Golet et al., 2015; Kenney et al., 1997; Logan, Golet, & Lutcavage, 2015; Murison & Gaskin, 1989; Siders, Westgate, Johnston, Murison, & Koopman, 2013; Stevenson & Scott, 2005).

Seabirds nest on islands throughout the GoM between mid‐spring (April) and late summer to early fall (August–September). Colony location can directly affect seabird nesting phenology. For example, common terns (*S. hirundo*) nesting on inshore islands may initiate egg laying earlier in the season compared to offshore islands (Hall & Kress, 2004). The timing and abundance of high‐quality forage (e.g., sand lance, herring, and other fishes) is critical for the provisioning, survival, and successful fledging of chicks (Diamond & Devlin, 2003). Egg laying, hatching, and fledging periods also vary among species. For example, razorbills (*Alca torda*) and murres (*Uria* spp.) leave nesting colonies as early as mid‐July, while terns (*Sterna* spp.) begin to leave in late July, and Atlantic puffins depart during August (Figure 5; GOMSWG, 2016; Yakola & Brofsky, 2016).

### 2.3 Fall: exit door southeast

Onset of fall is characterized by increased vertical mixing in surface waters resulting from wind and rapid convective cooling (Findlay, Yool, Nodale, & Pitchford, 2006). These drivers stimulate a fall phytoplankton bloom in the GoM that is smaller in magnitude but broader in duration than the spring bloom. Fall bloom timing has been correlated with SST and salinity (O'Reilly & Zetlin, 1998; Platt et al., 2010; Song et al., 2010; Thomas et al., 2003). Biomass and abundance of the copepod *C. typicus *peaks in fall, dominating GoM and Georges Bank zooplankton communities (Kane, 1993; Manning & Bucklin, 2005; Pendleton et al., 2009; Pershing et al., 2005). Atlantic herring are one of the few species of fish to spawn in fall, though timing varies among populations starting in late summer in the Bay of Fundy and going as late as November and December on Georges Bank (Stevenson & Scott, 2005; Walsh et al., 2015).

The most prominent aspect of the fall season is the mass exodus of many fishes, seabirds, and marine mammals either to offshore habitats or to other ocean basins throughout the Atlantic and Arctic. Many demersal and pelagic fishes move offshore into the deeper, more thermally stable waters of the continental slope and canyons (Friedland & Hare, 2007). Atlantic bluefin tuna leave the GoM over a period of months from October to December as temperatures decline and lipid reserves are fulfilled, largely from feeding on Atlantic herring (Mather, 1995; Wilson et al., 2005). Young of year anadromous fishes such as river herring emigrate from coastal freshwater ponds starting in late summer (July) through November to complete the juvenile portion of their life cycles in the open ocean (Iafrate & Oliveira, 2008; Kosa & Mather, 2001; Yako, Mather, & Juanes, 2002). Salmon smolts move within tributaries and mainstem habitats during the fall but are not believed to fully emigrate from freshwater systems (Meister, 1962). Adult American eels also transition into marine environments, mostly between September and November (Collette & Klein‐MacPhee, 2002).

Although many species move away from the coast in fall, a few fish and invertebrates exhibit an opposite pattern. Brooding northern shrimp females begin an inshore migration in late fall that culminates in the larval hatch period during mid‐ to late winter in nearshore (<50–100 m) waters (Haynes & Wigley, 1969; Richards, 2012). Adult Atlantic herring begin depositing demersal eggs as surface water temperatures decline in late summer through fall (Collette & Klein‐MacPhee, 2002; Stevenson & Scott, 2005), with larval abundance of fall‐spawned fish peaking in September and October (Figure 4a). Historically, there were two pulses of Atlantic salmon entering rivers. The major pulse was spring‐run fish that entered starting in April, and a fall run with fish entering rivers starting in September. A portion of the spring‐run population may stay in the lower reaches of the river before moving upstream and spawning with the fall run primarily in November (Belding & Kitson, 1934; Meister, 1962).

Seabirds tracked using satellite geolocator tags and radio telemetry were found to depart the GoM and other northern habitats for their wintering grounds starting in August. Some species such as Atlantic puffins and shearwaters (*Ardenna* spp., *Calonectris* spp., *Puffinus* spp.) are believed to overwinter in nearby habitats of the Gulf of St. Lawrence and Bay of Fundy (Fayet et al., 2017; Powers, Wiley, Allyn, Welch, & Ronconi, 2017), while others, such as roseate (*Sterna dougallii*), common (*S. hirundo*), and least terns (*Sternula antillarum*), migrate as far as South America, and Arctic terns (*S. paradisaea*) to the Southern Ocean (Egevang et al., 2010; Hays et al., 1997; Nisbet, 1984; Thompson et al., 1997; Veit & Petersen, 1993). Leach's storm‐petrels are one of the last seabirds to fledge their chicks, starting in August until as late as November, before departing for their wintering grounds including South America and the west coast of Africa (Pollet, Hedd, Taylor, Montevecchi, & Shutler, 2014).

### 2.4 Winter: cold dormancy

Gulf of Maine temperatures reach their annual minima in late winter (Richaud, Kwon, Joyce, Fratantoni, & Lentz, 2016). Historical SSTs on the continental shelf during the time period of 1854–2005 showed steady declines starting in August, reaching their lowest values in late February to early March, with some coastal water bodies icing over before warming again in spring (Friedland & Hare, 2007; True & Wiitala, 1990). Typically, surface phytoplankton concentrations are lowest over the Gulf's deep basins in winter. Coastal waters and shallow banks also have a winter phytoplankton minimum but remain somewhat elevated relative to the deep basins (e.g., Georges Bank; O'Reilly & Zetlin, 1998; Thomas et al., 2003). However, late winter blooms in deep basins were observed in 1999 (Durbin et al., 2003) and 2013 (Runge et al., 2015). While water column stratification is often argued as essential for phytoplankton bloom conditions, late winter blooms may be caused by deep light penetration coupled with low wind speeds (Townsend et al., 1994). In such cases, zooplankton populations can keep pace with winter blooms and may be observed at unusually high concentrations (Durbin et al., 2003; Runge et al., 2015). Apart from these rare episodic events, GoM copepod concentration and diversity reach annual lows during winter (Johnson et al., 2016; Kane, 1993, 2009; Manning & Bucklin, 2005; Record et al., 2010).

Many groundfish and pelagic species migrate and concentrate in deeper offshore waters of the outer shelf and slope during the colder months of the year (Collette & Klein‐MacPhee, 2002). Species including Atlantic cod (*Gadus morhua*), haddock (*Melanogrammus aeglefinus*), pollock (*Pollachius virens*), sand lance, and winter flounder (*Pseudopleuronectes americanus*) spawn during the colder months, and larval abundance peaks for 8 of 19 well‐surveyed larval taxa during January and February (Figure 4a).

Gray seals (*Halichoerus grypus*) are one of the few marine mammals to remain year‐round in the GoM. Adults haul‐out during January to breed and pup on coastal islands, beaches, and pack‐ice located throughout the GoM and further north in the Gulf of St. Lawrence (Klimova et al., 2014; Lesage & Hammill, 2001). Sea ice extent affects gray seal winter distributions, pupping success, and maternal energetics (Haller, Kovacs, & Hammill, 1996; Lesage & Hammill, 2001). Although gray seals use coastal mainland habitats for pupping, these areas are associated with increased disturbance from human activities (Lesage & Hammill, 2001). In contrast, preference for ice‐free waters drives harbor seals (*Phoca vitulina concolor*) to move into southern New England during the winter and spring (Baechler, Beck, & Bowen, 2002; Boehme et al., 2012; Burns, 2009). A portion of the North Atlantic right whale population and some seabirds continue to utilize habitats within the GoM during winter (Cole et al., 2013; Fayet et al., 2017), although their activities during this time are still largely unknown.

## 3 EVIDENCE FOR SHIFTING PHENOLOGY IN THE GoM

**Figure 6:**
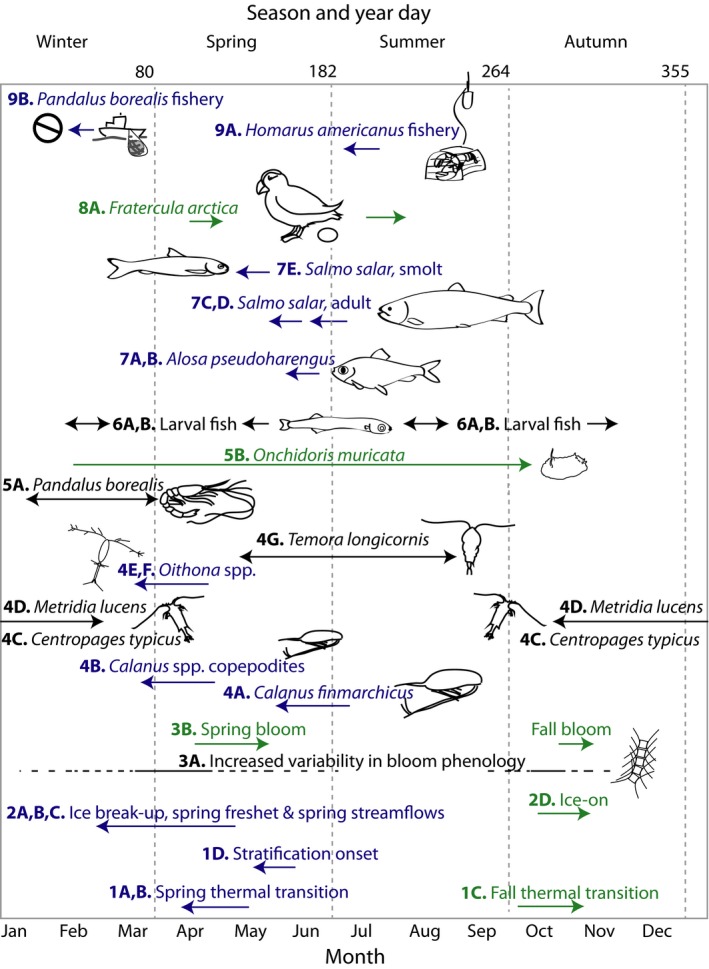
Summary of observed phenological shifts in the Gulf of Maine derived from a comprehensive literature search and expert input. Quantitative details of shifts are found in Table 1. Numbers and letters (e.g., 1A. Spring thermal transition) correspond to Table 1. Arrow color indicates directionality of shift in timing (blue = earlier, green = later) and arrow length depicts the magnitude of each shift, which corresponds to day of year, month, and season (bottom and top of figure). Original artwork by K. Stamieszkin [Colour figure can be viewed at wileyonlinelibrary.com]

Evidence for shifts in phenology was derived from a systematic literature search conducted in ISI Web of Knowledge using the term “phenology” in conjunction with “Northwest Atlantic” and the names of major GoM basins (see Figure 1 for complete list). Initially, 27 studies were found containing relevant information on regional coastal and marine organisms as well as environmental drivers (Supporting Information Appendix S3); however, close examination determined that only four of these studies (Friedland et al., 2015; Lambert, 2013; Richards, 2012; Thomas et al., 2017) explicitly demonstrated shifts in seasonal variables and species’ phenology over time. Two studies projected future changes in diapause duration in the zooplankton species *C. finmarchicus* (Pierson, Batchelder, Saumweber, Leising, & Runge, 2013; Wilson, Banas, Heath, & Speirs, 2016). The remaining 21 studies fell into three categories: (a) baseline studies documenting general patterns in timing or cycles of phenological and environmental events (e.g., Bouchard & Aiken, 2012); (b) modeling studies that explored mechanistic or spatial relationships between phenological events and environmental drivers, but did not evaluate changes in phenological responses over time (e.g., Zhai et al., 2011); and (c) modeling studies that advance our overall ability to understand responses to ecosystem perturbations and support future evaluations of shifts in phenology (e.g., Link, Fulton, & Gamble, 2010). Our author team's knowledge of specific species and systems identified 16 additional studies. The final list of 20 studies that provide direct evidence of phenological shifts in the GoM is summarized in Table 1 and Figure 6. Here we discuss these results in the context of regional climate drivers and future projected changes.

**Table 1:** Observed shifts in timing of biological and environmental events in the Gulf of Maine extracted from 20 studies identified through a literature review and expert input. Shifts are organized by environmental variable, functional ecological groups, and human activities. Numbers and letters (e.g., 1A. Spring thermal transition) correspond to Figure 6

Environmental variable or species	Phenological shift	Location	Years	Season	Environmental driver(s)	Reference
1. Oceanographic features						
A. Spring thermal transition	Progressively earlier by ≥ –0.5 days/year to surpassing a 12°C threshold since 1982.	GoM, Georges Bank, Northeast Shelf	1982–2014	All	Gulf Stream position, atmospheric pressure, and NAO	Thomas et al. (2017)
B. Spring thermal transition	For Georges Bank, eastern and western GOM, spring transition was relatively constant until 2006; between 2006 and 2016, spring transition advanced by 19 days.	Northeast shelf from Cape Hatteras to Nova Scotia	1982–2016	Spring	SST	Friedland et al. (2015)
C. Fall thermal transition	Progressively later by 1.0–1.5 days/years since 1982.	Northeast shelf from Cape Hatteras to Nova Scotia	1982–2014	All	Gulf Stream position, atmospheric pressure, and NAO	Thomas et al. (2017)
D. Stratification	In the eastern GoM, onset day shifted one week earlier in the recent decade relative to the long‐term mean. The western GoM exhibited strong interannual variability but no discernible trend was detected.	Eastern GoM	1978–2013	All	SST and salinity	Li et al. (2015)
2. Hydrography						
A. Ice‐affected stream flows (Ice break‐up)	Earlier last dates in 75% of rivers studied by 11 days on average, mostly since the 1960s.	Northern New England	1936–2000	Winter–spring	Winter–spring air temperatures	Hodgkins et al. (2005)
B. Spring freshet	Shifting earlier from May, to February, March, and April.	Northern New England	1902–2002	Winter–spring	Not specifically tested	Hodgkins et al. (2003)
C. Spring streamflows	Earlier occurrence of winter–spring streamflows in multiple river systems ranged from 4.4 to 8.6 days over 50 to 90‐year periods.	Northeast American rivers	1953–2002	Winter–spring	Air temperature, snowmelt runoff, and precipitation	Hodgkins and Dudley (2006a)
D. Ice‐affected stream flows (Ice‐on)	Later first dates of ice‐affected flows in 25% of rivers studied and decreased total duration of ice‐affected flows.	Northern New England	1936–2000	All	Winter–spring air temperature and precipitation	Hodgkins et al. (2005)
3. Primary production						
A. Phytoplankton	Increased variability of bloom phenology during the 1990s	GoM Basins	1961–2013	All	Not specifically tested	Record et al. (2016)
B. Phytoplankton	Spring and fall bloom mid‐points became later: 8.9 and 4.3 days per decade, respectively, since 1960.	GoM	1912–2015	All	Not specifically tested	Recd et al. (2018)
4. Secondary production						
A. *Calanus finmarchicus*	Adult and late‐stage annual spring increases in peak abundance shifted earlier, from approximately year day 200 to 150 after 1974; period of peak abundance shifted toward early May during 2001–2013.	GoM	1961–2013	All	Circulation and phytoplankton	Pershing et al. (2005, this study; Figure 3a)
B. *Calanus *spp.	Annual spring increase shifted progressively earlier from approximately year day 100 to 50 between 1961 and 2000; in recent years (2001–2013), this increase shifted back toward the long term (1961–2013) mean.	GoM	1961–2013	All	Circulation and phytoplankton	Pershing et al. (2005, this study; Figure 3b)
C. *Centropages typicus*	Elevated annual abundance lasted longer during fall and winter after 1992: approximately year day 150 to 100 (the following year), compared to year day 175 to 10 (the following year) pre−1992.	GoM	1961–2000	All	Circulation and phytoplankton	Pershing et al. (2005)
D. *Metridia lucens*	Elevated annual abundance got progressively longer, from approximately year day 300 to 25 (the following year) during years 1961–1974, to year day 250 to 110 (the following year) after 1992.	GoM	1961–2000	All	Circulation and phytoplankton	Pershing et al. (2005)
E. *Oithona* spp.	Annual low and subsequent increase in abundance shifted progressively earlier through time series, from approximately year day 100 to year day 50.	GoM	1961–2000	All	Circulation and phytoplankton	Pershing et al. (2005)
F. *Oithona *spp.	Annual low and subsequent increase abundance shifted from January/February to September/October between the 1980s and 1990s.	Georges Bank	1980–2000	All	Salinity, circulation	Kane (2007)
G. *Temora longicornis*	Peak abundance broadened from distinct July/August peak in the 1980s, to a broad May/June–July/August peak in the 1990s.	Georges Bank	1980–2000	All	Salinity, circulation	Kane (2007)
5. Macro‐invertebrates						
A. *Pandalus borealis*	Hatch initiation started earlier from a range of year days of 10 to 56 (median = 36); hatch completion ended later (year day 64 to 118 (median = 76)), resulting in overall increase in duration of 44 days (range 16 to 78 days).	Western GoM	1980–83; 1989–2011	Season of fishery (winter–spring)	Bottom and SST	Richards (2012)
B. *Onchidoris muricata*	Spawning extended into summer and recruitment expanded into early fall. Peak spawning shifted from January‐March to May‐June	Newcastle, NH	2005–2009	January–October	Not specifically tested	Lambert (2013)
6. Larval fish						
A. *Melanogrammus aeglefinus, Enchelyopus cimbrius, Pholis gunnellus, Anarhichas spp., Pseudopleuronectes americanus*	Larval occurrence shifted earlier in the winter (4 taxa) and summer (1 taxa) in 1999–2008 compared to 1977–1987.	Georges Bank, GoM	1977–1987 compared to 1999–2008	All	Not specifically tested	Walsh et al. (2015)
B. *Pollachius virens, Merluccius albidus,* *Ammodytes *spp., *Scomber scombrus *	Larval occurrence shifted later in the winter (2 taxa) and summer (2 taxa) in 1999–2008 compared to 1977–1987.	Georges Bank, GoM	1977–1987 compared to 1999–2008	All	Not specifically tested	Walsh et al. (2015)
7. Diadromous fish						
A. *Alosa pseudoharengus*	Spawning run initiation of adult fish shifted earlier by 13 days.	Southern New England streams	1973–1975 compared to 1997–2007	Did not specify, likely only relevant months	Temperature	Ellis and Vokoun (2009)
B. *Alosa pseudoharengus*	Median first capture of adults during spawning migration was earlier by 21.6 days, from approximately year day 165 to 143.	Androscoggin River, Midcoast Maine	1983–2001	Did not specify; likely only relevant months	Not specifically tested	Huntington et al. (2003)
C. *Salmo salar*	Median first capture of adults during spawning migration was earlier by 19.5 days, from approximately year day 195 to 175.	Penobscot River, Midcoast Maine	1986–2001	Did not specify; likely only relevant months	Not specifically tested	Huntington et al. (2003)
D. *Salmo salar*	Date of 50% total return of adult fish migrating to freshwater spawning grounds shifted 10 days earlier, from approximately year day 158 to 148.	Penobscot River, Midcoast Maine	1978–2001	Did not specify; likely only relevant months	River temperature and flow tested; neither significant	Juanes et al. (2004)
E. *Salmo salar*	Smolt seaward migration shifted to earlier dates, from an approximate mean of year day 148 to 135.	North Atlantic Ocean Basin, including Gulf of Maine	1961–2010	March‐August	Air, river, and sea temperatures	Otero et al. (2014)
8. Seabirds						
A. *Fratercula arctica*	Mean date of egg laying shifted later from year day 123 to 135 since 1980; and mean date of fledging shifted from year day 210 to 219 since 1995.	Machias Seal Island, coastal GOM	1980–2014	May–August	Oceanographic conditions affecting prey quality	Whidden (2016) (Masters thesis)
9. Human activities						
A. *Homarus americanus fishery*	Landings peaked 22 days earlier and the season extended longer than usual in 2012 than the mean date from 1981–2011	GoM	1981–2012	All	Anomalous high temperature	Mills et al. (2013)
B. *Pandalus borealis fishery*	Estimated year day of hatch midpoint shifted earlier from 67 to 44; hatch onset was the earliest on record in 2012, leading to early attainment of catch limit and fishery closure.	Cape Ann, MA ‐ Penobscot Bay, ME	1998–2012	All	SST and chlophyll‐a concentration	Richards et al. (2016)

### 3.1 Historical observations and future projections of oceanographic changes

#### 3.1.1 Seasonal cycle of warming and cooling

The seasonal cycle of warming and cooling has undergone significant changes as temperatures have risen. Warming trends were strongest during summer (July ‐ September) at rates of about 1.0°C/decade (1982–2014) in the central GoM (Friedland & Hare, 2007; Thomas et al., 2017). Conversely, average air temperatures over terrestrial environments in New England showed the greatest increases during winter, rising at rates of 0.18°C/decade (1895–2017), which is 2–3 times faster than in other seasons over the past century (Horton et al., 2014; NCEI, 2017). Changes in temperature cycles have shifted the seasonal metrics that define the warmest period of the year. Summer start and end dates in the GoM, as defined by SST thresholds, have shifted approximately 1 day per year since 1982, lengthening the summer period by as much as 2 days per year during this time (Thomas et al., 2017). Initial investigations suggest these changes are associated with regional patterns, such as the Gulf Stream position, atmospheric pressure, and North Atlantic Oscillation (NAO; Thomas et al., 2017). Similar metrics—the spring and fall thermal transition times, each defined as the day of year when a seasonally specific temperature threshold is surpassed for ≥8 consecutive days—have shifted more rapidly in the past decade, with the spring transition advancing two weeks since 2006, and fall transition occurring progressively later with the greatest changes since 2005 (Friedland et al., 2015; Thomas et al., 2017; seasonal updates available at Northeast Fisheries Science Center (NEFSC): http://www.nefsc.noaa.gov/ecosys/current-conditions). Analysis of 26 climate models in the CMIP5 ensemble using the “business as usual” (RCP 8.5) scenario shows warming trends of 0.4°C per decade until the end of this century, but the ensemble of models does not project this warming to further shift the annual cycle in the region (Alexander et al., 2018). In addition, changes in basin‐scale indices of natural climate variation, such as the NAO, Atlantic Multidecadal Oscillation (AMO), and Gulf Stream position, are likely amplifying regional warming and precipitation cycles, and have the potential to further affect seasonal shifts in the environment over the coming decades (Saba et al., 2016; Thomas et al., 2017).

#### 3.1.2 Seasonal stratification and mixing

The seasonal stratification of the water column has shown temporal (both interannual and decadal) and spatial variability. Unlike changes in the thermal cycle that are coherent over the broader Northwest Atlantic region, stratification timing displays substantial regional heterogeneity. The stratification onset day is computed for each year as the date when stratification strength first exceeds 25% of its 1978–2013 median. Over the 35‐year study period, the stratification onset day has fluctuated 1–3 weeks, with distinct patterns in the eastern and western GoM. In the eastern GoM, stratification onset shifted one week earlier in the most recent decade relative to its 35‐year mean date, compared to a 1‐ to 2‐week delay in the late 1980s and 1990s (Li et al., 2015). In the western GoM, stratification onset follows 1–2 week interannual variations with no significant trend over either the entire period or the most recent decade. Regional differences in the response of stratification timing reflect differential effects of changing ocean temperature and salinity (Li et al., 2015).

In recent years, changes in the character and timing of seasonal water mass inputs and outputs through the Northeast Channel have been observed. The Northeast Channel is the primary conduit connecting offshore slope water with deep basins in the GoM. In the early 2000s, the pattern of flow through the deep channel transitioned from a relatively stable flow structure with episodic variability to strongly seasonal; notably, deep water in the channel exhibited consistent reversals of flow in the spring and fall (Smith, Pettigrew, Yeats, Townsend, & Han, 2012). Changes in surface currents (e.g., the Gulf of Maine Coastal Current) and deep water exchanges are driven by interactions between the Gulf Stream and Labrador currents; changes in these currents are causing an increase in the frequency of anomalous warm water pulses and altered supply dynamics of nutrient‐rich deep slope water into the GoM (Brickman, Hebert, & Wang, 2018; Pettigrew et al., 2005; Ramp, Schlitz, & Wright, 1985).

#### 3.1.3 Seasonal shifts in freshwater inputs

Inputs of freshwater to the GoM are driven by the seasonal hydrological cycle over the watershed as well as transport of comparatively fresher water into the GoM from the Scotian Shelf. There is strong evidence for shifts in the timing of winter–spring hydrology and seasonal runoff into coastal areas (Dudley, Hodgkins, McHale, Kolian, & Renard, 2017; Hodgkins, Dudley, & Huntington, 2003). On average, coastal watersheds in the GoM region have experienced an increase in monthly (except January through March) and annual precipitation from 1895 to 2010 (Huntington & Billmire, 2014). There has been an increase in the intensity and persistence (i.e., probability of rainfall occurring on multiple consecutive days) of precipitation from the mid‐20th century to the early 21st century throughout the northeastern United States (Guilbert, Betts, Rizzo, Beckage, & Bomblies, 2015; Madsen & Wilcox, 2012). Pronounced warming during winter and spring is leading to more rain instead of snow, more rain on snow events, as well as decreased snowpack duration, depths, and snow water equivalents in late winter and early spring (Hamburg, Vadeboncoeur, Richardson, & Bailey, 2013; Hodgkins & Dudley, 2006a; Huntington, Hodgkins, Keim, & Dudley, 2004; Vincent et al., 2015). As the snowpack absorbs more liquid from rainfall and melts earlier, high spring river flows, calculated as the winter–spring center‐of‐volume date, have occurred 1–2 weeks earlier in New England, New Brunswick, and Nova Scotia in the 20th and early 21st centuries (Dudley et al., 2017; Hodgkins & Dudley, 2006b; Hodgkins et al., 2003; Vincent et al., 2015). Hodgkins, Dudley, and Huntington (2005) showed significantly earlier last dates (11 days on average) of spring ice‐affected flows (i.e., earlier river ice breakup) in 75% of the New England rivers studied between 1936 and 2000, with most changes observed to have occurred since the 1960s. Changes during fall have been less prominent, with only 25% (4/16) of the rivers studied exhibiting significantly later first dates of ice‐affected (i.e., ice‐on) flows (Hodgkins et al., 2005). In addition, Vincent et al. (2015) showed trends toward earlier river ice breakup in spring at sites in southern Canada for 1950–2012.

Streamflow and runoff magnitudes have also changed in New England rivers in the 20th and early 21st centuries (Huntington & Billmire, 2014). Monthly streamflow trends in 27 regional streams with minimum human influences showed increases in March and decreases in May (Hodgkins & Dudley, 2005). Increases were also observed for most streams in winter and fall, and decreases in summer, but the trends were weaker. Similar trends occurred in monthly streamflow and associated dissolved organic carbon (DOC) export for major rivers draining to the GoM from 1930 to 2013 (Huntington et al., 2016). Collectively, these studies (Table 2) provide evidence that freshwater and DOC export are occurring earlier in the year (generally more in March and April and less in May and June) and that export has increased in the winter, and to a lesser extent in the fall. Climate model simulations predict continued shifts in precipitation and runoff, with increases in magnitude during winter but not in summer for the 21st century, and earlier timing of high spring flows (i.e., earlier winter–spring center‐of‐volume dates) in New England (Campbell, Driscoll, Pourmokhtarian, & Hayhoe, 2011; Hayhoe et al., 2007; Thibeault & Seth, 2014).

**Table 2:** A synthesis of observed changes in hydrological variables influencing the Gulf of Maine region. ↑ = increasing in magnitude, ↓ = decreasing, ← = advancing in time

Hydrologic metric	Winter	Spring	Summer	Fall	Annual	References
Precipitation		↑	↑	↑	↑	Keim, Fischer, and Wilson (2005); Hayhoe et al. (2007); Huntington and Billmire (2014); Vincent et al. (2015)
Rainfall intensity and frequency of large streams					↑	Madsen and Wilcox, (2012); Kunkel et al. (2013); Guilbert et al. (2015)
Duration of dry episodes (>30 days)			↑			Groisman and Knight (2008)
Runoff	↑	↑ in March, April; ↓in May		↑	↑	Hodgkins and Dudley (2005); Huntington and Billmire (2014); Huntington et al. (2016)
Timing of spring freshet		←				Hodgkins et al., (2003); Hodgkins and Dudley (2006b)
Occurrence of river ice	↓					Hodgkins et al. (2005); Vincent et al. (2015)
Timing of river ice‐out		←				Hodgkins et al. (2005); Vincent et al. (2015)
Thickness of river ice	↓					Huntington et al. (2003)
Variance in mean decadal precipitation					↑	Balch, Drapeau, Bowler, and Huntington (2012)
Fluvial export of DOC	↑	↓		↑	↑	Huntington et al. (2016)
Ratio of solid to liquid precipitation					↓	Huntington et al., (2004); Vincent et al. (2015)
Density of late winter snowpack	↑					Hodgkins and Dudley (2006b); Vincent et al. (2015)

### 3.2 Phytoplankton

Phytoplankton bloom phenology differs across the region depending upon the temporal period considered. The timing of the annual spring bloom in the GoM did not show linear trends between the 1980s and 2013 (Record et al., 2016; Song et al., 2010; Thomas et al., 2003), nor on the Scotian Shelf between 1998 and 2008 (Platt et al., 2010). During the 1990s, bloom timing was more variable than compared to the 1980s and 2000s (Record et al., 2016). Over multidecadal time scales, spring and autumn bloom timing both shifted later; rates depended on which phenology metric was used, with the most significant rates at 8.9 and 4.3 days per decade since 1960, for spring and autumn, respectively, when tracking the midpoint of a Gaussian fit (Record, Balch, & Stamieszkin, 2018). Earlier spring blooms were correlated with higher February stratification and lower nutrients (Record et al., 2018), whereas later spring blooms were associated with higher springtime surface salinity (Ji et al., 2007, 2008; Song et al., 2010). Lagged temperature conditions (2–8 months before the bloom) may be important in nearshore areas (Richards, O'Reilly, & Hyde, 2016). Fall bloom timing was consistently found to be negatively correlated with SST and salinity, with higher temperatures and salinity resulting in earlier blooms (Ji et al., 2007, 2008; Record et al., 2018; Song et al., 2010). The variation in responses and associated drivers observed across bloom studies in the region is likely due in part to the oceanographic and atmospheric conditions specific to the time period examined (i.e., season, decade), but may also be related to sampling techniques, choice of phenology metric, and the temporal resolution and methodology used to evaluate each dataset (Ferreira, Visser, MacKenzie, & Payne, 2014).

### 3.3 Zooplankton

The phenology and succession of copepod species in the GoM has shifted over the past several decades. The seasonal cycle from 1961 to 2013 was dominated by *C. finmarchicus* (during winter and spring), followed by a successional period of smaller species (including *C. typicus, Pseudocalanus *spp., *M. lucens, *and *Oithona similis* during fall and winter; Record et al., 2016). Peak timing of abundance for many of these species has shifted and appears to be driven primarily by changes in water mass transport into the GoM, resultant changes in stratification, and phytoplankton phenology (Kane, 2007, 2011; Pershing et al., 2005; Record et al., 2016). Specifically, in the 1980s, the turnover of the different copepod species followed a regular pattern of succession, but in the 1990s, the successional pattern broke down, yielding a less regular pattern typical of an ecological disturbance (Pershing et al., 2005; Record et al., 2016). This change mirrored the regional phytoplankton phenology discussed above.

The timing of peak spring abundance for *C. finmarchicus* has demonstrated high variability and shifts in the GoM region relative to changes in water circulation and zooplankton community structure (MERCINA, 2004; Pershing et al., 2005; Record et al., 2016). Continuous Plankton Recorder (CPR) data show progressively earlier annual spring increases in abundance and higher annual maximum concentrations of adult and subadult (CV‐CVI) *C. finmarchicus* in surface waters over the time series 1961–2013 (Figure 3a, Supporting Information Appendix S2). These trends are more pronounced in juvenile *Calanus* spp. (CI‐CIV) with the overall duration of the annual cycle and maximum concentrations exhibiting increases, though inconsistently, across the four time periods in the historical record (Figure 3b, Supporting Information Appendix S2). Spring concentrations of juveniles increased earlier during more recent periods, shifting from April (1961–1974) to February–March (1978–2013). Later and more gradual declines in abundance were also apparent in late summer, shifting from July during 1961–1974 to August in 2001–2013 (Figure 3b, Supporting Information Appendix S2). Under future climate change, additional shifts in phenology are projected for *C. finmarchicus*, including reduced duration of diapause by more than a month (~39 days) with a 2℃ temperature increase (Pierson et al., 2013) and at least 50% reductions in overwintering dormancy by end of century (2090–2099 compared to 2000–2009) under the RCP 8.5 scenario (Wilson et al., 2016). Projected shifts are size‐dependent and potentially moderated by behavioral changes such as overwintering at deeper depths.

### 3.4 Fish

The most extensive evaluation to date of shifting phenology in GoM fishes was conducted by Walsh et al. (2015). This study combined data from two long‐term ichthyoplankton surveys (Ecosystem Monitoring (EcoMon); Marine Resources Monitoring, Assessment, and Prediction (MARMAP)) to compare changes in the seasonal occurrence of 45 larval fish species between two decades (1977–1987 and 1999–2008) across the Northeast Shelf ecosystem. Of the 19 most abundant fish species examined in the GoM and Georges Bank, larval occurrence of five species shifted earlier, four shifted later, and 10 showed no change between the two periods. Significant shifts were not observed in spring or fall taxa (Figure 4b). Winter taxa were expected to shift later due to later fall onset and warmer water temperatures; however, later shifts were only observed in 2 (sand lance and pollock) of the eight winter species, four winter species shifted earlier (haddock, winter flounder, wolffishes (*Anarhichas* spp.), rock gunnel (*Pholis gunnellus*)) and 2 showed no change (Atlantic cod, Longhorn Sculpin (*Myoxocephalus octodecemspinosus*)). Summer taxa also exhibited variable phenological responses with fourbeard rockling shifting earlier, offshore hake and Atlantic mackerel shifting later, and two other species (Silver hake, cunner (*Tautogolabrus adspersus*)) showing no change (Figure 4b). Overall, results suggest warming is influencing the seasonal occurrence of fish larval stages in complex and inconsistent ways. Although the underlying mechanisms are currently unknown, shifts may be indicative of changes in adult migration and spawning times, larval survival rates, or a combination of these and other factors, particularly during summer and winter. Walsh et al. (2015) also explored shifts in the seasonal occurrence of adult fishes using data from the NEFSC bottom trawl survey but found no definitive evidence for phenological changes.

Shifts in migration timing have been documented in a number of adult and juvenile diadromous fishes throughout the greater GoM region. The median date of arrival of adult Atlantic salmon migrating up the Penobscot River shifted earlier, with advancements of approximately 16 days between 1978 and 2001 (0.7 days/year; Juanes, Gephard, & Beland, 2004) and 19 days between 1986 and 2001 (1.3 days/year; Huntington et al., 2003); stream temperature and flow were identified as the most significant drivers of observed shifts. On a broader scale, emigration of Atlantic salmon smolts to the marine environment throughout their range in the North Atlantic Ocean has advanced 2.5 days/decade between 1961 and 2010 in response to warming in freshwater and marine habitats (Otero et al., 2014). Advances in the timing of spawning migrations of alewife of approximately 22 days have been observed in the Androscoggin River in Maine between 1983 and 2001 (1.2 days/year; Huntington et al., 2003). In addition, Ellis and Vokoun (2009) used water temperatures in four Massachusetts rivers to estimate alewife migration timing had shifted 12–13 days earlier between 1971–1978 and 1997–2007.

### 3.5 Macro‐invertebrates

Shifts in reproductive cycles were found in two macro‐invertebrates inhabiting the GoM. Between 1980 and 2011, the winter/spring hatching period for Northern shrimp commenced progressively earlier and ended progressively later, resulting in an increased overall duration of 44 days of the hatching period (Richards, 2012). Northern shrimp hatch timing is tuned to local bottom temperature throughout their range (Koeller et al., 2009), and the phenology of key reproductive stages has been linked to bottom and SSTs in the GoM with evidence of earlier hatching in warmer years (Richards, 2012). The shift in reproductive timing may also have an impact on early life stage survival, which was negatively correlated with phytoplankton biomass several weeks before the midpoint of the hatch period during 1998–2012 (Richards et al., 2016). A proposed explanation is that plankton species available as food to newly hatched shrimp are less favorable later in the bloom succession than during bloom initiation (Richards et al., 2016).

In a second case, the intertidal dorid nudibranch (*Onchidoris muricata*) expanded its spawning and recruitment periods into summer and early fall, respectively. Peak spawn production was observed to shift from January–March to May–June between surveys conducted in 1970s–1980s and a more recent period in the early 2000s (Lambert, 2013). Although the driver for this shift in phenology remains unresolved, nudibranchs were found in association with a novel prey species of invasive bryozoan (*Membranipora membranacea*), which may have contributed to this shift in addition to warming water temperatures (Dijkstra et al., 2011; Lambert, 2013).

### 3.6 Seabirds

Studies of Atlantic puffins show egg laying and chick fledging shifted later on Machias Seal Island since the mid‐1990s and early 1980s, respectively (Whidden, 2016). Nesting success and fledging condition also declined, possibly due to phenological mismatch with preferred forage fish abundance and changes in spring conditions (Keogan et al., 2018; Kress, Shannon, & O'Neal, 2016).

### 3.7 Human activities

As the timing of biological events change, human activities such as fishing, shipping, and recreation are also expected to change, though often at a lag with natural resources. The timing of human activities has been affected in some invertebrate fisheries in the region. A shift occurred in the American lobster fishery during a marine heat wave in 2012 (Mills et al., 2013). Warming spring water temperatures are associated with lobster molting, inshore migration, and higher activity levels, all of which made the species more available to the Maine coastal fishery. During 2012, temperatures warmed three weeks earlier than normal, and the commercial lobster fishery responded by shifting into its high‐volume summer mode 3–4 weeks earlier, resulting in much higher landings of lobster during June and July than during typical years (Mills et al., 2013). Anomalously warm ocean temperatures also extended the period of fall cooling, which resulted in unusually high landings through the remainder of the year (Mills et al., 2013). Similarly, shifts in migration timing affected the northern shrimp fishery in the GoM, which consists of a winter trawl and trap fishery that exploits brooding females. In 2012, shrimp hatch onset was the earliest on record; the fishery landed its catch limit and closed early, thereby limiting fishing opportunities, particularly for trappers, which begin later than trawling effort (ASMFC, 2012).

## 4 Implications of changing phenology

### 4.1 Ecosystem implications

The strong seasonal cycle in the GoM is an important component of the region's ecology, as is typical for temperate marine ecosystems (Conover, 1992; Edwards & Richardson, 2004). The wide range of conditions experienced by organisms in the GoM throughout an annual cycle necessitates life histories tuned to particular seasons. Therefore, changes in the timing of seasonal environmental and ecological features could impact individual species, food webs, and overall ecosystem productivity. The magnitude of these impacts will depend upon the extent to which ecosystem responses create asynchronies or mismatches with environmental conditions, life history events, and among species including novel trophic interactions (Cushing, 1990; Durant, Hjermann, Ottersen, & Stenseth, 2007; Miller‐Rushing, Høye, Inouye, & Post, 2010; Staudinger et al., 2013; Thackeray et al., 2010).

Shifts and variability in the timing of physical events such as seasonal transitions, onset of water column stratification, or seasonal delivery of freshwater, nutrients, and DOC, can have cascading non‐linear and episodic effects on the timing of various coastal processes including ocean surface transparency, phytoplankton productivity (Balch et al., 2016; Gobler et al., 2017), episodic hypoxia (Diaz & Rosenberg, 2008), and acidification (Bauer et al., 2013; Gledhill et al., 2015). Projected decreases in DOC export during spring and summer (Huntington et al., 2016) could influence stream metabolism by reducing labile carbon for heterotrophic bacterial respiration in benthic and pelagic environments (Meyer, Tate, Edwards, & Crocker, 1988; Volk, Volk, & Kaplan, 1997). These changes, in turn, could affect the supply of energy to higher trophic levels in downstream coastal ecosystems. Changes in the timing and amount of DOC export to coastal waters (Hodgkins & Dudley, 2006b; Hodgkins et al., 2003; Huntington et al., 2016) likely influence marine biogeochemistry, including the development of nuisance and harmful algal blooms (Hayes et al., 2001) as well as carbon sequestration (Schlünz & Schneider, 2000). The amplification or dampening of spring blooms is also dependent on temporal overlap with grazer populations. For example, zooplankton and planktivorous fish abundance can moderate bloom overgrowth in some coastal systems if winter/spring emergence, growth, and/or grazer abundance aligns with bloom timing.

Although widely discussed in the ecological literature, observed in situ examples of mismatches in global marine communities remain scarce. The theory of ecological mismatches known as the “match‐mismatch hypothesis” was originally conceived by Cushing (1969) as a bottom‐up mechanism for explaining variation in year‐class strength of fishes. This hypothesis proposed that good year classes resulted from spatiotemporal alignment (match) of primary and secondary food resources and the pelagic larval period, while poor year classes occurred when there were mismatches (Cushing, 1969, 1990; Peck, Huebert, & Llopiz, 2012). Yet, the underlying mechanisms that alter the timing of phytoplankton blooms and definitive links between secondary production and upper trophic levels are complex, variable, and highly uncertain (Edwards & Richardson, 2004; Friedland et al., 2015; Richards et al., 2016). Phytoplankton bloom timing, magnitude, and composition have species‐ and location‐specific impacts on recruitment (Platt, Fuentes‐Yaco, & Frank, 2003; Trzcinski, Devred, Platt, & Sathyendranath, 2013), but the duration of spatiotemporal overlap between larval fish and prey may be paramount to year‐class success (Kristiansen, Drinkwater, Lough, & Sundby, 2011).

Differential rates of change in temperature and other environmental conditions across GoM habitats can result in mismatches in optimal physiology in migratory species that have sublethal effects on fitness and survival (Friedland, Manning, & Link, 2012; Friedland et al., 2012; Wood & Kellermann, 2015). In addition, climate‐induced range shifts (Kleisner et al., 2016; Nye et al., 2009) are altering community composition along migration corridors and in destination habitats, potentially influencing predator–prey interactions (Friedland et al., 2012; Kordas, Harley, & O'Connor, 2011; Walsh et al., 2015). For example, it is hypothesized that Atlantic salmon post‐smolts may be experiencing historically atypical marine prey and predator fields, resulting from changing ocean conditions and annual cycles (e.g., Thomas et al., 2017) that negatively impact growth and survival (Friedland et al., 2012; Friedland, Shank, Todd, McGinnity, & Nye, 2014; Renkawitz, Sheehan, Dixon, & Nygaard, 2015).

The timing and relative abundance of lipid‐rich prey such as sand lance and Atlantic herring is thought to be critical to sustain higher level predators such as seabirds, which rely on these species for provisioning chicks (Burthe et al., 2012). On seabird breeding colonies in the GoM, warm water‐associated fishes such as butterfish are being fed to chicks and associated with starvation events; consumption of butterfish, which are oval in shape and difficult to swallow, and other low‐quality prey are thought to represent mismatches in primary prey availability (Kress et al., 2016). A recent review concluded global seabird populations have not adjusted their breeding phenology over time (1952–2015) or in response to rising SSTs (Keogan et al., 2018). Given that shifts in timing and distribution have been documented for several primary prey species in the GoM including sand lance, pollock, hake, and fourbeard rockling (Walsh et al., 2015), differential responses, or lack thereof, aligns with the mismatch hypothesis but needs to be formally tested. The seasonal availability of lower quality prey or a mismatch in size (either too small or too big) may be more important to some species than relative abundance (Golet et al., 2015). As has been shown in bluefin tuna, such mismatches may not necessarily result in mortality, but rather influence population dynamics through longer growing and development periods (Golet et al., 2015). This has also been observed in harbor seals, where changes in average gestation length and parturition date were attributed to decreased food availability between 1990 and 2000 (Bowen, Ellis, Iverson, & Boness, 2003).

### 4.2 Implications for fisheries

The efficacy of fishery management tools including fixed seasons, catch limits, and time‐area closures may be compromised when target resources shift more rapidly than dependent fisheries or regulations (Peer & Miller, 2014; Pinsky & Fogarty, 2012). Fisheries that are not regulated by fixed seasons may be able to respond to phenological changes in their target species with more flexibility; however, the supply chain and market system are not necessarily as adaptable (Mills et al., 2013). In addition, shifting phenology can alter the availability of different portions of the stock (e.g., year class or life stage), which can influence how benefits are distributed to fishery participants. The 2012 heat wave provided an example of how changes in the timing of the availability of target resources could affect invertebrate fisheries. The high volume and early timing of the American lobster fishery landings in 2012, coupled with an unusual overlap with the Canadian lobster season, exceeded the capacity of the supply chain (Mills et al., 2013). The ultimate result was a market glut and price collapse that substantially affected individual fishermen (Mills et al., 2013). In contrast to the U.S. fishery, the Canadian lobster management system opens the fishery for set periods of time, with the open season in Scotia‐Fundy lobster fishing areas aligned to target hard shell lobster (Pringle & Burke, 1993). Warmer ocean temperatures are associated with higher proportions of soft shell lobsters during the fishing season (Thakur et al., 2017). These lobsters are more fragile than hard shell lobsters, making them hard to ship and store, and considered poorer quality. Thus, the early warming during 2012 affected the Canadian fishery, not only through the general price collapse, but also through a shift toward increasing catches of a lower‐value component of the stock.

Northern shrimp have supported a fishery in the GoM since the 1930s (Scattergood, 1952). The fishery is managed through controls on open season length and timing as well as total allowable catch (ASMFC, 2011). Earlier reproductive timing and hatching during warmer years (2012 in particular) has sometimes led to a partial mismatch between the availability of shrimp and the open fishing season, with more severe impacts on the late seasonal trap sector (Richards et al., 2012). Other regional finfish fisheries face similar temporal misalignment challenges. Fishermen have raised concerns that silver hake (whiting) now often migrate north of the fishing area by the time the season opens. This resulted in the consideration of a new Amendment by the New England Fishery Management Council (Limited Access Amendment 22) in 2018 on where and when the whiting fishery should be open in the GoM in the future.

In Maine and New Hampshire coastal rivers, earlier ice thinning and melting during the winter and early spring has led to contracted ice fishing seasons for smelt. During warmer years, poor ice integrity has necessitated earlier removal of ice huts due to safety issues to fishermen. Seasonal fishing camps and related industries experience reduced revenue due to fewer fishing days during years when onset of winter is later and spring comes earlier, thus threatening the sustainability of this cultural tradition and popular recreational activity (Personal communication, Maine Department of Marine Resources).

Interactions between climate and fishing pressure can change mortality rates on one or more portions of a population, leading to altered life history diversity and increased sensitivity to cumulative stressors over time (Ohlberger, Thackeray, Winfield, Maberly, & Vollestad, 2014). One manifestation of these consequences has been shown in the Mid‐Atlantic Bight where striped bass migration occurs earlier with higher temperatures; however, the fishing season is static and set to calendar dates intended to enable early migrants to spawn before the season begins. When waters warm earlier, more striped bass move upstream before the fishing season, thereby reducing fishing mortality on spawning females during warm years (earlier runs) in comparison to cool years (later runs; Peer & Miller, 2014). In this case, spawning females are benefiting from shifting phenology, but there may be unintended consequences for other portions of the population and these patterns may not hold true in other systems. When the timing of seasonal migration and aggregation becomes more variable and less predictable, spatial and temporal management approaches are more likely to result in incidental take or bycatch and compromise the protection and recovery of vulnerable species (Dunton et al., 2012, 2015).

For many species, fisheries management depends on predictions of stock productivity over the near‐term to set annual quotas and catch limits. These predictions are often derived from stock assessment models that are parameterized using historical relationships between catch, spawning stock biomass (SSB) or egg production, and abundance of recruits (Beverton & Holt, 1993). In ecosystems that are changing in complex ways with no historical analogue, these relationships may not remain true. Shifts in early life history phenologies (e.g., Walsh et al., 2015) and changes in seasonal timing can further complicate the already difficult task of estimating population productivity, abundance, and appropriate harvest levels for marine species (Henderson, Mills, Thomas, Pershing, & Nye, 2017; Sissenwine & Shepherd, 1987). For example, a recent meta‐analysis evaluated the effects of spring onset and summer duration on seasonal mean stratified biomass of 43 stocks of adult fish and invertebrates on the U.S. Northeast shelf between 1982 and 2014 (Henderson et al., 2017). Longer summers were found to result in extended growing seasons and increased biomass for most temperate stocks, including summer and winter flounder, spiny dogfish, and haddock at 0‐ to 5‐year lags. Conversely, cold‐water species, such as Atlantic cod exhibited decreased biomass at a 1‐year lag, suggesting longer periods of suboptimal temperatures could reduce population recruitment (Henderson et al., 2017).

Spatiotemporal closures have also been used to protect spawning aggregations of important fisheries such as Atlantic cod (Armstrong et al., 2013), Atlantic herring (ASMFC, 2012, 2016), and haddock (Halliday, 1988). Changes in the timing of larval fish occurrence could indicate that the timing of adult spawning in the U.S. Northeast shelf ecosystem has shifted (Walsh et al., 2015). However, temporal closures are typically set to predetermined dates; thus, as spawning times change or are better understood, closure times may need to be adjusted to maintain efficacy (Armstrong et al., 2013). For some species, such as Atlantic herring, ongoing monitoring of spawning condition of the fish is conducted to modify closure dates so they align with actual spawning times (Richardson, Hare, Overholtz, & Johnson, 2010; Wurtzell et al., 2016). Because the timing of closure dates can require multiple years of monitoring and rule iterations prior to implementation (Armstrong et al., 2013), adaptive management practices are needed to ensure closed spawning areas meet intended objectives and are flexible enough to accommodate increasing variability and extreme years (Walters, 1986).

### 4.3 Seasonal management measures

Seasonal management measures serve a variety of management and conservation purposes. Seasonal management areas (SMA), designated shipping channels, and fishery regulations (e.g., 322 Code Mass. Regs. § 12.00 (2013)) have been used to reduce human–wildlife interactions and avoid essential habitat of endangered and protected species such as the North Atlantic right whale. For right whales, the National Marine Fisheries Service (NMFS) implemented vessel speed restrictions within 10 SMAs along the U.S. East Coast, including in the GoM, with the goal of minimizing collisions by vessels during specific times of the year (NMFS, 2008, 2013). Right whales have been occurring at unexpected times and places, and Dynamic Management Areas (DMAs) were implemented to address this issue by establishing protective zones around aggregations of right whales outside of SMAs (NMFS, 2008). With the right whale population at a critically low level (Pace, Corkeron, & Kraus, 2017), there is a need for additional real‐time tools that provide information on whale occurrences (e.g., Whale Alert, 2018: http://www.whalealert.org/), estimate occupancy probability in known habitats, characterize phenological trends, and identify new areas where right whales may be likely to occur (Meyer‐Gutbrod & Greene, 2018).

Changes in whale or bird migration and breeding times can interact with human activities in both positive and negative ways. With spring arriving earlier and summer lasting longer, tourism and outdoor recreation in coastal areas are likely to increase. While whale and bird watching industries may benefit from longer tourism seasons that increase revenue (Nelson et al., 2013), these and other seasonal activities could act to increase disturbance to wildlife during critical life stages, further complicate existing conflicts, and may require additional actions or closures. For example, beach closures and restricted vehicle access are an ongoing management issue for spring and summer nesting shore and seabirds, such as the federally threatened piping plover (*Charadrius melodus*). Harbor seals are also sensitive to human disturbance during their spring–summer pupping season when pups are bound to coastal haul‐out sites, potentially leading to increased abandonment and stranding incidents (Curtin & Garrod, 2008; Newsome & Rodger, 2008).

Coastal alteration projects such as beach nourishment and restoration activities, as well as dredge and fill operations, pose threats to fish and wildlife due to general habitat disturbances, increased suspended sediments in the water column, lower dissolved oxygen, and direct mortality to some species. Time of year (TOY) restrictions on in‐water work are routinely used to protect critical life stages by establishing periods during which species use spatially constrained habitats or are particularly vulnerable to stress (Evans, Diamond, & Kelly, 2015). TOY restrictions are typically established based on average conditions, with some room for annual variation. In Massachusetts, recommended TOY restrictions for diadromous alewives are currently set between April 1 and June 15 to protect spawning migrations and habitats. However, during recent years, alewife were observed migrating upriver as early as late February–early March (Annual Summaries from Massachusetts Division of Marine Fisheries, Association to Preserve Cape Cod (APCC), and Massachusetts Bays National Estuary Program (MassBays)). If shifts are occurring in these systems, regulatory agencies may need to reconsider the duration of TOY windows, increase monitoring to support in‐season modification during extreme years, and/or use precautionary temporal buffers that allow for greater variation in phenology (Evans et al., 2015). Similar considerations will also apply to hydropower and water drawdown operations that affect seasonal flows and aquatic‐marine habitat connectivity.

### 4.4 Marine spatial planning

Industrial marine activities such as oil and gas exploration and offshore wind development require information to be collected on species of conservation, commercial, and recreational interest throughout the year. These and other human activities create potential conflicts between resource use and conservation needs and have led to the advancement of Marine Spatial Planning as a framework for more holistic ecosystem management. For example, placement of structures and operation schedules within migration corridors has been raised as a major concern for sea and shorebirds, marine mammals, and some fishes. Several state and regional plans have been released or are near completion, including the Northeast Ocean Plan (Northeast Regional Planning Body, 2016), Massachusetts Ocean Management Plan (Massachusetts Energy & Environmental Affairs, 2015), and Rhode Island Ocean Special Area Management Plan (2010). Although non‐binding, these plans document and characterize resource and habitat use, which support siting and other decisions that may reduce conflicts among resource users and protect species’ habitat needs. Although data collection related to climate‐induced changes in phenology is encouraged (e.g., Chapter 5, Northeast Ocean Plan), the majority of information gathered and used across these efforts is static and lacks the temporal and spatial resolution needed to inform management actions that account for seasonal migration, reproduction, or other essential habitat use. This information could inform when activities related to the construction or installation of new projects should be avoided and when operations should be restricted to avoid disruptions to highly sensitive species and critical phenological events. Further, because offshore wind turbines exist on 20‐ to 30‐year leases, current Environmental Impact Assessments (EIA) do not sufficiently consider potential changes in migrations and habitat use within the lifetime of the projects. Increased consideration of seasonal or higher resolution temporal data and projections of habitat occupancy and suitability would strengthen future EIAs (Ryan, Danylchuk, & Jordaan, 2018).

## 5 CONFOUNDING FACTORS FOR DETECTING AND UNDERSTANDING SHIFTS IN PHENOLOGY

### 5.1 Spatial versus temporal shifts

A range of sampling design and ecological factors can confound the detection and interpretation of phenological signals. In terrestrial studies, particularly for plants, researchers have been able to isolate changes in temporal variables due to the stationary or location‐based characteristics of the study system (de Keyzer, Rafferty, Inouye, & Thomson, 2016; Primack et al., 2009). In contrast, disentangling temporal and spatial shifts in highly dynamic marine systems is difficult, if not impossible, due to challenges associated with detecting and tracking marine organisms under water. Some of the longest marine data series in the greater Northwest Atlantic region (e.g., NEFSC bottom trawl survey; NEFSC ecosystem monitoring surveys for oceanographic conditions and plankton (EcoMon)) utilize stratified random sampling designs in which exact sampling locations vary during each survey. Most animals sampled are mobile or planktonic, and depending on their level of site fidelity of life history stage, may not consistently occur in the same location over seasonal and annual time periods. Shifts in survey timing, geographic heterogeneity, sampling intensity within and across strata, and changes in survey spatial coverage can confound detection and interpretation of phenological changes (Jordaan et al., 2012; de Keyzer et al., 2016). In addition, sampling an entire marine ecosystem is costly and can be affected by budget shortfalls, which result in a lack of sampling platforms, gaps in long‐term time series and in some cases, the complete loss of a time series (e.g., NEFSC Continuous Plankton Recorder sampling aboard Ships of Opportunity). Tracking phenology within a defined area and taking into consideration habitat and species diversity at the population and community scales are all important factors to overcoming these challenges (de Keyzer et al., 2016). While study design and data collection methodology are clearly important for capturing phenological events, the advancement of this field often requires using long‐term historical datasets that were not collected with the same methods or with phenology in mind. Therefore, choice of phenology metric, preprocessing (e.g., combining time series, data smoothing, filtering), and analytical techniques also play important roles in overcoming data deficiencies as well as isolating and interpreting signal from noise across spatiotemporal scales (Ferreira et al., 2014).

### 5.2 Effects of population changes on phenology

Variation in population size and structure (size, age, sex) due to natural fluctuations, fishing mortality, or conservation efforts may confound detection of phenological shifts (Tillotson & Quinn, 2018). Different life history stages may have unique phenologies, while density‐dependent effects can also influence seasonal events such as the timing and duration of migration and spawning. For example, the timing of river herring and Atlantic herring movements is related to the size of individual fish, with larger fish arriving first on spawning grounds (Lambert, 1987; Marjadi et al., 2019). If the proportion of large fish in the population changed over time, this could appear as a shift in spawning phenology whether or not it was real. Changes in the proportion of non‐breeding individuals in a population may also confound phenological signals. For example, movement patterns of breeding and non‐breeding seabirds differ greatly, with non‐breeding individuals lacking the strict arrival and colony‐centered constraints of breeding birds. Thus, how questions are framed, the choice of phenology metrics (e.g., first or mean arrival time), and a firm understanding of how population size and composition may be changing over time are important factors to consider when interpreting perceived shifts in timing (Ferreira et al., 2014). In addition, novel interactions can result as species expand their ranges and shift residence times in seasonal habitats where they were previously absent or only intermittently present (Collie et al., 2008; Wood et al., 2009), as in the case of the documented bryozoan–nudibranch interaction (Lambert, 2013; Lambert, Bell, & Harris, 2016).

### 5.3 Variation in methodology and metrics for assessing phenology

There are a number of confounding factors that can impede assessments of phenology in the marine environment and beyond. Perhaps most importantly is the selection of the most suitable phenology indicator (i.e., characteristic that may be changing over time) and metric (i.e., estimator of change) that aligns best with the question of interest (Ferreira et al., 2014). When studies target or discuss similar indicators (e.g., arrival) but use different metrics (e.g., first vs. mean arrival time) to track changes over time this compromises precision, introduces error, and can be especially problematic in multi‐species analyses or impact assessments (Ferreira et al., 2014). Marine and aquatic studies vary substantially in how phenological shifts have been evaluated and reported. Some studies relate shifts in timing to environmental conditions (Juanes et al., 2004). In other cases, biological events are presented in the context of, or in parallel with environmental drivers, but not directly related (Huntington et al., 2003; Walsh et al., 2015). A few studies have developed metrics or indices that incorporate environmental drivers to help understand timing such as the weighted‐mean migration temperature developed for diadromous fishes (Ellis & Vokoun, 2009; Quinn & Adams, 1996). Further, phenological shifts have been measured over continuous time periods (e.g., Zhai et al., 2011) as well as between discrete historical and contemporary periods (e.g., Walsh et al., 2015).

Many phenology studies rely on presence/absence records to track the timing of species occurrence. Ideally, a phenology study would have multiple non‐detections (absences) before the first detection of a phenological event. However, in marine systems such data may be difficult to collect with confidence over certain spatiotemporal scales and types of events. Metrics that take into account detection uncertainties (e.g., percentiles of cumulative occurrences) may be more appropriate for characterizing phenological shifts, for example, in highly migratory species (e.g., Dufour, Arrizabalaga, Irigoien, & Santiago, 2010), where there are known gaps in spatiotemporal coverage, knowledge of population‐wide patterns is poor, and/or in cases where species’ behavior could produce multiple detections of the same individuals (e.g., sentinels moving in and out of a system). Choice of metric also depends on whether a species is being tracked at the individual level (e.g., from tag numbers or photo analyses: Ramp, Delarue, Palsbøll, Sears, & Hammond, 2015) or at the population level, where information on individuals and demographics is largely unknown. In addition, using changes in one life stage as a proxy for plasticity in another can lead to spurious conclusions if life stages are not synchronized or are exhibiting differential responses to environmental cues (Rosset et al., 2017; Walsh et al., 2015).

Clearly identifying how an indicator is measured and explicitly defining metrics reduces confusion and unintended errors when interpreting and comparing results among systems. Further, capturing the true magnitude of variation in metric estimates over time may better indicate when a population or community is approaching a threshold and potential regime shift (Pearse, Davis, Inouye, Primack, & Davies, 2017). New modeling approaches that account for bias and uncertainty are emerging and can overcome some sampling and data deficiencies (Ferreira et al., 2014; Pearse et al., 2017). Communication and coordination between scientists and monitoring programs of how best to track indicators will also improve studies of marine phenology.

Lastly, it is important to note that there is likely a strong publishing bias toward reporting significant responses and omitting stable results. Parmesan (2007) noted that the omission of non‐significant findings provides an incomplete view of phenological responses and inflates the strength of the observed responses. Non‐detectable responses (no shift) may indicate a species is adapting in place (Beever et al., 2016), and important to report as there are implications for mismatches with other species that are shifting. Therefore, to gain a complete and balanced understanding of how phenology is changing in the GoM, future studies should consider reporting significant and non‐significant shifts.

## 6 ADAPTATION STRATEGIES RELATED TO PHENOLOGY

As direct and indirect impacts of climate change are increasingly evident across the GoM, adaptation strategies are critically needed to reduce vulnerability and uncertainty to cumulative stressors, as well as to increase ecosystem resilience and adaptive capacity (Beever et al., 2016; Heller & Zavaleta, 2009; Stein et al., 2013). Adaptation strategies could better account for phenological change through expanded, coordinated, and high‐resolution monitoring programs (to track changes), vulnerability assessments (to prioritize focus areas), and forecast models and dynamic management tools (to improve decision‐making) that consider ongoing and projected temporal system changes.

### 6.1 Monitoring

A highly dynamic system like the GoM may require multiple decades of observations to detect shifts and separate confounding factors from true phenological drivers (Cohen, Lajeunesse, & Rohr, 2018). An average of approximately 40 years of data was considered in the twenty studies that provided direct evidence of shifts in this synthesis. The lack of definitive relationships between seasonal temperatures and marine phenological responses globally (Poloczanska et al., 2013) and regionally (this study) suggests temperature alone may not be sufficient for explaining shifts. Species respond to multiple cues that are changing predictably (photoperiod) and differentially (e.g., temperature and hydrology), thus simultaneous monitoring, more sophisticated modeling (Pearse et al., 2017), and consideration of multiple drivers are needed (Jonsson & Jonsson, 2009). Such integrative studies are becoming possible through collaborative partnerships and big data networks such as the Northeastern Regional Association of Coastal Ocean Observing Systems (NERACOOS: www.neracoos.org/), as well as citizen science and volunteer monitoring programs. These efforts seek to increase the availability of an array of high‐quality environmental and ecological datasets for broad‐scale climate‐relevant analyses but need to be updated regularly and have sufficient coverage to support phenological studies. Examples of long‐term monitoring programs in the GoM that if properly maintained could be used to further explore phenology‐related questions are compiled in Supporting Information Appendix S4.

Coordinated regional monitoring that builds on historical datasets of community composition and occurrence, migration timing, and life stage‐specific information across broad temporal periods and seasonal habitats is needed to test theories of how changes in phenology affect species interactions (Staudinger et al., 2013). However, some long‐term monitoring programs are being scaled back (e.g., Continuous Plankton Recorder Survey) at a time when expanded effort is needed to capture ecosystem‐level changes. Surveys that are fixed in time (i.e., effort begins and ends on predetermined days of year) are particularly susceptible to inadvertently missing shifts in phenological events. It has been hypothesized that species that have historically migrated in and out of the region might become year‐round residents if conditions change substantially (Ramp et al., 2015). However, potential for year‐round residency is limited by the need for concurrent shifts in prey or novel interactions. Determining whether a greater proportion of a population is remaining in the region year‐round or, conversely, detecting increasing variability or non‐linear effects will help distinguish if species are adapting to novel conditions or approaching thresholds and regime shifts (Pearse et al., 2017; Powell, Tyrrell, Milliken, Tirpak, & Staudinger, 2017).

### 6.2 Assessing risk and vulnerability

Climate Change Vulnerability Assessments (CCVA) have become widely used to synthesize how species and systems will be exposed to climate impacts and their expected responsiveness given inherent traits related to sensitivity and adaptive capacity (Beever et al., 2016; Glick, Stein, Edelson, and (Eds.)., 2011; IPCC, 2007). Methodology for CCVAs varies widely, and phenological responses may be evaluated explicitly (e.g., changes in migration timing) or indirectly (e.g., changes in growth, production, and reproduction: Berry, Ogawa‐Onishi, & McVey, 2013; Hare et al., 2016; ICES, 2017; Johnson & Welch, 2010; Pacifici et al., 2015; Staudinger, Morelli, & Bryan, 2015). More traditional assessments such as the State Wildlife Action Plans are beginning to consider climate‐related threats and cite shifts in phenology as causing ecological disruptions (e.g., predator–prey), disturbance to life cycles, and decreased reproductive success as a concern for some Regional Species of Greatest Conservation Need (Northeast Fish & Wildlife Diversity Technical Committee, 2016); yet, corresponding actions are still largely undefined. Additional monitoring and modeling of seasonal oceanographic processes and timing of ecological events will be useful to inform risk assessment and adaptation actions for regional species, ecological communities, and human activities.

### 6.3 Forecast models and dynamic management tools

Research on fine‐scale spatiotemporal oceanographic processes for the GoM has been limited due to the lack of a regional downscaled model. Future projections based on the ensemble of CMIP5 models show strong warming in all months, but strongest in summer (Alexander et al., 2018). Higher resolution models better resolve patterns in the regional Atlantic circulation and water mass distribution, and project much stronger (almost twice as fast) warming than the coarser resolution models (Saba et al., 2016). Improvements and extensions to current models and development of other high‐resolution models improve our ability to assess impacts and species responses in GoM subregions.

Several forecasts of phenological events have been developed (Payne et al., 2017), including one in the GoM of the threshold conditions and timing of when the Maine lobster fishery can be expected to shift into its high landings summer mode (Mills, Pershing, & Hernández, 2017). This model uses buoy‐based temperature observations and historical fishery data to forecast the timing of the uptick in fishery landings 3–4 months in advance (Mills et al., 2017). Similar models that rely on temperature observations, biological lags, and historical temperature–biology relationships have been developed in other regions to forecast the timing of salmon runs (e.g., Anderson & Beer, 2009). Short‐term forecasts such as NOAA's Gulf of Maine Region Quarterly Climate Impacts and Outlook (http://www.gulfofmaine.org/public/climate-network/climate-outlook/) are another early warning tool to help regional communities and managers prepare and adapt to extreme years. The expansion of forecast models for a wider range of climatic conditions, species, life history events, and management applications may become more widespread in the GoM with advancements in understanding of how phenology is changing, as impacts become more well known, and stakeholder needs arise (Hobday, Spillman, Paige Eveson, & Hartog, 2016; Payne et al., 2017).

## 7 FUTURE RESEARCH AVENUES FOR PHENOLOGY IN THE GULF OF MAINE

Key gaps in knowledge revealed through this synthesis can guide new research initiatives to reduce uncertainty and help regional managers prepare for increasingly variable conditions in the GoM. In many cases, strong anecdotal information exists pertaining to possible changes in phenology for valued and potentially vulnerable functional groups, but rigorous phenology‐focused analyses of existing data sets have yet to be performed. Where possible, we highlight potential long‐term survey and monitoring datasets that may be useful for evaluating phenological responses and ecological disruptions. However, substantial effort may be needed to digitize, compile, and/or organize these existing resources into formats conducive for addressing questions at the appropriate spatiotemporal scale. The use of statistical estimators (Pearse et al., 2017) and gap‐filling or other preprocessing techniques (Cole et al., 2013; Ferreira et al., 2014) may enhance the value of existing data sets for investigating phenology changes.

### 7.1 Oceanographic changes

The timing and occurrence of extreme seasonal events such as marine fog and coastal storms can vary widely over local scales and have major impacts on species movements, ecological processes, and human navigation. Information on coastal storms including tropical cyclones, hurricanes, and Nor'easters is increasing, but reporting is largely at annual scales. Tropical storms and hurricanes have been increasing in occurrence and intensity, and storm tracks are shifting northward in U.S. Atlantic coastal areas (Frumhoff, McCarthy, Melillo, Moser, & Wuebbles, 2007; Holland & Webster, 2007). With oceans warming earlier in the year and reaching greater overall temperatures (Thomas et al., 2017), the possibility of changes in timing of extreme events as well as their frequency and magnitude is increased. Occurrence of extreme events during summer is of particular concern for GoM species that complete critical life stages during this time such as colonial nesting seabirds, pupping pinnipeds, and spawning fish populations.

### 7.2 Phytoplankton and Zooplankton

Satellite‐based and in situ studies of phytoplankton bloom timing have found that SST, salinity, and nutrients can impact the timing of spring and fall blooms to varying degrees (Ji et al., 2007, 2008; Record et al., 2018; Song et al., 2010). Yet, additional research is needed to determine whether and how changes in timing, availability, and community composition cascade up to affect higher trophic levels. For example, diapause duration of zooplankton, especially *C. finmarchicus*, is related to lipid levels accumulated during this critical period of energetic development. If this period is truncated (Pierson et al., 2013; Wilson et al., 2016), larval fish survival and the energetic condition of higher level predators such as North Atlantic right whales that forage on copepods could be affected (Friedland et al., 2015; Irigoien, Harris, Head, & Harbour, 2000; Pendleton et al., 2009; Trzcinski et al., 2013). While earlier bloom timing has been related to longer bloom duration on a global scale (Friedland et al., 2018), the hypothesis that earlier, longer blooms act to increase the probability of overlap with larval fish during their critical period (Friedland et al., 2016) remains untested in the GoM. Understanding these multi‐trophic interactions will be key to advancing phenological research in the region and beyond.

### 7.3 Diadromous fish

Diadromous fishes offer a rare example of migratory marine organisms that are relatively easy to monitor. Because they make multiple transitions between freshwater and marine habitats through fixed and often highly visible locations such as dams and culverts, their migration and spawning timing can be routinely observed (Martins, Hinch, Cooke, & Patterson, 2012). Diadromous fish are a multi‐phylogenetic group that produce a range of egg sizes, exhibit early and late maturation, and reach maturity over small and large body sizes as well as a variety of different schedules (Gross, 1987); thus, phenological change may vary widely among taxa. In addition, tributary characteristics (e.g., stream morphology and depth), habitat conditions (flow and temperature), and buffering capacity to climatic extremes (Chezik, Anderson, & Moore, 2017) are expected to influence populations differently across their range. Adult alewife and Atlantic salmon migrations have been relatively well tracked in several GoM tributaries (Ellis & Vokoun, 2009; Huntington et al., 2003; Juanes et al., 2004). However, a meta‐analysis of latitudinal variation in timing, environmental drivers, and population estimates across diadromous species and regions would help determine whether populations are keeping pace with changing conditions, maintaining population strength, or exhibiting localized declines. Increased effort is needed to capture entire life cycles, especially the growth, survival, and emigration (e.g., river herring) of juveniles as they transition from freshwater to marine systems. Additional information on these aspects of life history and phenology would help resolve underlying drivers of year‐class strength, population dynamics, and species interactions.

### 7.4 Marine fish

Demersal and pelagic fishes and invertebrates have been routinely monitored by programs such as spring and fall bottom trawl surveys conducted by federal and state agencies (Azarovitz, 1981; King, Camisa, & Manfredi, 2010; Sherman, Stepanek, Pierce, Tetrault, & O’Donnell, 2015). However, these are broad‐scale surveys, and spatial and temporal coverage may not be adequate for evaluating nuanced changes in phenology (Hare et al., 2016). Sampling at higher temporal frequencies within small, fixed, or ecologically important areas (e.g., spawning grounds) is valuable for tracking phenological change. Increased collection and synthesis of phenological data across multiple life stages would increase understanding of species’ adaptive capacity and improve vulnerability analyses, especially for larval and juvenile stages, and species that make multiple ontogenetic transitions between distinct habitats (Hare et al., 2016; Petitgas et al., 2013).

Fisheries‐dependent data have the potential to provide local‐scale information on multiple species. Historical records of daily landings and observer data from fish weirs and regional fishing ports are ideal for assessing changes in phenology due to their fixed locations, long seasons of operations, and historical archives (Alexander et al., 2017; Matthiessen & Toner, 1963). Newspaper fishing reports, online forums, fishermen's personal knowledge, and traditional ecological knowledge of tribal nations also serve as alternative or supplemental data sources.

### 7.5 Seabirds

Long‐term phenology records exist for a number of breeding seabirds in databases maintained by state, federal (U.S. Fish and Wildlife Service), academic (e.g., University of New Brunswick), and nonprofit organizations (e.g., National Audubon Seabird Restoration Program) in the GoM. Observations include chick provisioning, hatching, productivity, chick condition, and return rates that can be used to address current management and conservation concerns related to phenology (Kress et al., 2016; Northeast Fish & Wildlife Diversity Technical Committee, 2016). For non‐breeding seabirds that utilize GoM habitats (e.g., shearwaters), phenology data may be limited and difficult to acquire because these species are rarely observed from land, and spatial distributions over open ocean habitats are patchy and unpredictable. A major knowledge gap for this group is how environmental and ecological conditions on and total distances to overwintering grounds influence the timing of movements to and from the GoM. Satellite tags have yielded some recent successes in tracking short‐term, local movements in the GoM (Powers et al., 2017), but methods are not yet sufficient or designed to track long‐term phenological patterns.

Phenology research on understudied seabirds has the potential to be augmented through citizen science programs such as eBird (http://ebird.org) and the USA National Phenology Network, 2018 (https://usanpn.org/). Through these platforms, amateur and professional birders can contribute a diversity of observations with precise timing and location information as trip reports (checklists) that are stored in a central database, from which data can be queried and analyzed to evaluate specific questions. To date, application of these online databases has focused on studies of terrestrial songbirds and comparatively few seabird species. Substantial occurrence data exist for marine species in some areas of the GoM, particularly within eBird, and when paired with overlapping environmental data (e.g., satellite data), has the potential to evaluate shifts and asynchronies in phenology (e.g., Mayor et al., 2017).

### 7.6 Marine mammals and other large pelagics

To the best of our knowledge, no studies published to date have documented phenological shifts in marine mammals in the GoM. However, just to the northeast in the Gulf of St. Lawrence, Ramp et al. (2015) documented advancements in arrival, departure, and residence times by humpback and fin whales over a 27‐year period. Occurrences of these and other whales, as well as large pelagic animals such as seals, basking sharks, bluefin tuna, and sea turtles, have been monitored since the early 1980s using systematic aerial and shipboard marine mammal surveys (Brown, Kraus, Slay, & Garrison, 2007; CeTAP, 1982). While survey effort has been variable, many of these datasets could support the development of occupancy and habitat suitability models to evaluate and verify anecdotal observations of occurrences in increasingly uncharacteristic locations in the GoM at anomalous times of the year (e.g., North Atlantic right whales; The Chronicle Herald, 2015).

For commercially harvested species such as highly migratory tunas, fisheries‐dependent data from commercial longlining or recreational charter boats could provide a basis for understanding seasonal habitat use as long as the influence of weather, economic conditions, and fishing regulations were carefully considered (Dufour et al., 2010). A key question pertaining to a range of marine mammals and other highly migratory species that use the GoM as a seasonal foraging ground is whether changes in seasonal onset and duration will continue to support primary prey species. If forage fishes such as sand lance, Atlantic herring, and mackerel become less predictable or mismatched, predators (e.g., bluefin tuna) may change their migration patterns accordingly. Alternatively, longer growing seasons could increase forage availability and residence time of seasonal migrants in the GoM. Such shifts have implications for regional trophic dynamics (e.g., predatory demand on prey populations) and could expose some species to stressors (e.g., fishing activities) not experienced at the same magnitude elsewhere in their life history. Information on this guild could be improved by establishing routine fishery‐independent sampling, tracking movements using acoustic and satellite tagging technologies, and working cooperatively with fishermen that maintain detailed logbooks of catches, as has occurred in Atlantic sturgeon (Dunton et al., 2015; Melnychuk et al., 2017).

## 8 SUMMARY AND CONCLUSIONS

Research on marine phenology has a long history in the GoM region, yet the majority of studies conducted to date have focused primarily on documenting and describing baseline patterns and cycles in seasonal events or developing modeling frameworks to resolve mechanistic relationships of species responses to environmental drivers. The results of this synthesis yielded a surprisingly small number of studies (*N* = 20) showing direct evidence of shifts in timing in biotic and abiotic events. It is possible this is an artifact of under‐reporting non‐significant results, and indicative of stable populations that are adapting in place (Beever et al., 2016; Parmesan, 2007). Similar to previous research in terrestrial systems (Miller‐Rushing et al., 2008; Parmesan & Yohe, 2003; Primack et al., 2009), the most common phenological responses found in the GoM were advancements in (earlier) timing, notably spring onset, spring and winter hydrological metrics, zooplankton abundance, some larval fishes, and diadromous fish migration patterns. Later timing was limited to fall onset, spring and fall phytoplankton blooms, occurrence of a few larval fishes, and reproduction and fledging in one species of seabird (Atlantic puffin). Changes in the duration of phenological events generally increased, including abundance peaks in zooplankton, spawning/early life history periods of macro‐invertebrates, and lobster fishery landings. Ice‐affected streamflow was the only seasonal event exhibiting a reduction in duration, and two studies projected decreased diapause duration in the future for the zooplankton species, *C. finmarchicus* (Table 1). Overall, rates of phenological shifts were species‐ and event‐specific, and responses varied depending on the environmental driver and the spatial and temporal scales evaluated.

This comprehensive review summarizes the state of the science of shifting phenology in the GoM region and identifies infomation gaps related to taxonomic groups of high conservation and management concern. Our findings demonstrate a clear need for increased emphasis on phenological research in the region and should serve as a catalyst for future investigations. We have highlighted a number of case studies where actions can be taken to reduce uncertainty and guide adaptation efforts to avoid disruption of the ecosystem services in a rapidly changing ocean.

## References

[fog12429-bib-0001] Agler, B. A. , Schooley, R. L. , Frohock, S. E. , Katona, S. K. , & Seipt, I. E. (1993). Reproduction of photographically identified fin whales, Balaenoptera physalus, from the Gulf of Maine. Journal of Mammalogy, 74(3), 577–587. 10.2307/1382276

[fog12429-bib-0002] Alert, W. (2018). *Whale Alert [App]* Retrieved from http://www.whalealert.org/

[fog12429-bib-0003] Alexander, K. E. , Leavenworth, W. B. , Willis, T. V. , Hall, C. , Mattocks, S. , Bittner, S. M. , … Jordaan, A. (2017). Tambora and the mackerel year: Phenology and fisheries during an extreme climate event. Science Advances, 3(1), e1601635 10.1126/sciadv.1601635 28116356 PMC5242558

[fog12429-bib-0004] Alexander, M. A. , Scott, J. D. , Friedland, K. D. , Mills, K. E. , Nye, J. A. , Pershing, A. J. , & Thomas, A. C. (2018). Projected sea surface temperatures over the 21st century: Changes in the mean, variability and extremes for large marine ecosystem regions of Northern Oceans. Elem Sci Anth, 6(1), 9 10.1525/elementa.191

[fog12429-bib-0005] Anderson, J. J. , & Beer, W. N. (2009). Oceanic, riverine, and genetic influences on spring chinook salmon migration timing. Ecological Applications, 19(8), 1989–2003. 10.1890/08-0477.1 20014573

[fog12429-bib-0006] Armstrong, M. P. , Dean, M. J. , Hoffman, W. S. , Zemeckis, D. R. , Nies, T. A. , Pierce, D. E. , … McKiernan, D. J. (2013). The application of small scale fishery closures to protect Atlantic cod spawning aggregations in the inshore Gulf of Maine. Fisheries Research, 141, 62–69. 10.1016/j.fishres.2012.09.009

[fog12429-bib-0007] ASMFC (2011). *Amendment 2 to the Interstate Fishery Management Plan for Northern Shrimp* (p. 87). Retrieved from http://www.asmfc.org/uploads/file/northernShrimp_Amendment2_2011.pdf

[fog12429-bib-0008] ASMFC . (2012). *Addendum 1 to Amendment 2 to the Interstate Fishery Management Plan for Northern Shrimp* (p. 15). Retrieved from http://www.asmfc.org/uploads/file/noShrimpAddendumI_Nov2012.pdf

[fog12429-bib-0009] ASMFC . (2016). *Amendment 3 to the Interstate Fishery Management Plan for Atlantic Herring* (p. 106). Retrieved from http://www.asmfc.org/uploads/file/57042f26Amendment3_RevisedApril2016.pdf

[fog12429-bib-0010] Azarovitz, T. R. (1981). A brief historical review of the Woods Hole laboratory trawl survey time series In Doubleday W. G., & Rivard D. (Eds.), Bottom trawl surveys. Canadian Special Publication of Fisheries and Aquatic Sciences, 58, 62–67. Retrieved from http://www.ices.dk/sites/pub/CM%20Doccuments/1997/Y/1997_Y33.pdf

[fog12429-bib-0011] Baechler, J. , Beck, C. A. , & Bowen, W. D. (2002). Dive shapes reveal temporal changes in the foraging behaviour of different age and sex classes of harbour seals (*Phoca vitulina*). Canadian Journal of Zoology, 80(9), 1569–1577. 10.1139/z02-150

[fog12429-bib-0012] Balch, W. M. , Drapeau, D. T. , Bowler, B. C. , & Huntington, T. G. (2012). Step‐changes in the physical, chemical and biological characteristics of the Gulf of Maine, as documented by the GNATS time series. Marine Ecology Progress Series, 450, 11–35. 10.3354/meps09555

[fog12429-bib-0013] Balch, W. , Huntington, T. , Aiken, G. , Drapeau, D. , Bowler, B. , Lubelczyk, L. , & Butler, K. (2016). Toward a quantitative and empirical dissolved organic carbon budget for the Gulf of Maine, a semienclosed shelf sea. Global Biogeochemical Cycles, 30(2), 268–292. 10.1002/2015GB005332

[fog12429-bib-0014] Bauer, J. E. , Cai, W.‐J. , Raymond, P. A. , Bianchi, T. S. , Hopkinson, C. S. , & Regnier, P. A. G. (2013). The changing carbon cycle of the coastal ocean. Nature, 504(7478), 61–70. 10.1038/nature12857 24305149

[fog12429-bib-0015] Beever, E. A. , O'Leary, J. , Mengelt, C. , West, J. M. , Julius, S. , Green, N. , … Hofmann, G. E. (2016). Improving conservation outcomes with a new paradigm for understanding species’ fundamental and realized adaptive capacity: A new paradigm for defining adaptive capacity. Conservation Letters, 9(2), 131–137. 10.1111/conl.12190

[fog12429-bib-0016] Belding, D. L. , & Kitson, J. A. (1934). Spring‐run and fall‐run Atlantic salmon. Transactions of the American Fisheries Society, 64(1), 225–230. 10.1577/1548-8659(1934)64[225:SAFAS]2.0.CO;2

[fog12429-bib-0017] Berry, P. , Ogawa‐Onishi, Y. , & McVey, A. (2013). The vulnerability of threatened species: Adaptive capability and adaptation opportunity. Biology, 2(3), 872–893. 10.3390/biology2030872 24833051 PMC3960872

[fog12429-bib-0018] Beverton, R. J. H. , & Holt, S. J. (1993). On the dynamics of exploited fish populations (first). Dordrecht, the Netherlands: Springer Netherlands.

[fog12429-bib-0019] Black, G. A. P. , Rowell, T. W. , & Dawe, E. G. (1987). Atlas of the biology and distribution of the squids Illex illecebrosus and Loligo pealei in the Northwest Atlantic. Ottawa, ON: Department of Fisheries and Oceans Retrieved from http://www.dfo-mpo.gc.ca/Library/106107.pdf

[fog12429-bib-0020] Boehme, L. , Thompson, D. , Fedak, M. , Bowen, D. , Hammill, M. O. , & Stenson, G. B. (2012). How many seals were there? The global shelf loss during the last glacial maximum and its effect on the size and distribution of grey seal populations. PLoS ONE, 7(12), e53000 10.1371/journal.pone.0053000 23300843 PMC3530534

[fog12429-bib-0021] Bouchard, G. M. , & Aiken, R. B. (2012). Latitudinal variation in the reproductive cycle and size of the northern rock barnacle *Semibalanus balanoides* (L.) (Cirripedia, Archaeobalanidae) in the Bay of Fundy. Crustaceana, 85(7), 779–787. 10.1163/156854012X650214

[fog12429-bib-0022] Bowen, D. , Ellis, S. L. , Iverson, S. J. , & Boness, D. J. (2003). Maternal and newborn life‐history traits during periods of contrasting population trends: Implications for explaining the decline of harbour seals (*Phoca vitulina*), on Sable Island. Journal of Zoology, 261(2), 155–163. 10.1017/S0952836903004047

[fog12429-bib-0023] Brickman, D. , Hebert, D. , & Wang, Z. (2018). Mechanism for the recent ocean warming events on the Scotian Shelf of eastern Canada. Continental Shelf Research, 156, 11–22. 10.1016/j.csr.2018.01.001

[fog12429-bib-0024] Brown, M. W. , Kraus, S. D. , Slay, C. K. , & Garrison, L. P. (2007). Surveying for discovery, science, and management In Kraus S. D., & Rolland R. M. (Eds.), The urban whale: North Atlantic right whales at the crossroads (pp. 105–137). Cambridge, MA: Harvard University Press.

[fog12429-bib-0025] Burns, J. J. (2009). Harbor seal and Spotted seal: *Phoca vitulina* and *P. largha* In Perrin W. F., Würsig B., & Thewissen J. G. M. (Eds.), Encyclopedia of marine mammals (2nd ed., pp. 533–542). London, UK: Academic Press.

[fog12429-bib-0026] Burthe, S. , Daunt, F. , Butler, A. , Elston, D. a. , Frederiksen, M. , Johns, D. , … Wanless, S. (2012). Phenological trends and trophic mismatch across multiple levels of a North Sea pelagic food web. Marine Ecology Progress Series, 454, 119–133. 10.3354/meps09520

[fog12429-bib-0027] Campana, S. E. , Gibson, J. , Brazner, J. , Marks, L. , Joyce, W. , Gosselin, J.‐F. , & Lawson, L. (2008). *Status of basking sharks in Atlantic Canada (No. 2008/004)* . Fisheries and Oceans. Retrieved from http://uni.hi.is/scampana/files/2016/01/basking-shark-Res-Doc-2008_004_e.pdf

[fog12429-bib-0028] Campbell, J. L. , Driscoll, C. T. , Pourmokhtarian, A. , & Hayhoe, K. (2011). Streamflow responses to past and projected future changes in climate at the Hubbard Brook Experimental Forest, New Hampshire. United States. Water Resources Research, 47(2), W02514 10.1029/2010WR009438

[fog12429-bib-0029] CETAP (1982). *Characterization of marine mammals and turtles in the Mid‐ and North Atlantic areas of the U.S. Outer Continental Shelf. Final Report. contract AA551‐CT8‐48* . Bureau of Land Management, U.S. Department of Interior, Washington D.C. # pp. 570. Retrieved from http://www.nefsc.noaa.gov/psb/docs/CETAP%201982%20Final%20Report.pdf

[fog12429-bib-0030] Chezik, K. A. , Anderson, S. C. , & Moore, J. W. (2017). River networks dampen long‐term hydrological signals of climate change: River networks dampen climate change. Geophysical Research Letters, 44, 7256–7264. 10.1002/2017GL074376

[fog12429-bib-0031] Clapham, P. J. , & Mayo, C. A. (1987). Reproduction and recruitment of individually identified humpback whales, *Megaptera novaeangliae*, observed in Massachusetts Bay, 1979–1985. Canadian Journal of Zoology, 65(12), 2853–2863. 10.1139/z87-434

[fog12429-bib-0032] Cohen, J. M. , Lajeunesse, M. J. , & Rohr, J. R. (2018). A global synthesis of animal phenological responses to climate change. Nature Climate Change, 8(3), 224–228. 10.1038/s41558-018-0067-3

[fog12429-bib-0033] Cole, T. V. N. , Hamilton, P. , Henry, A. G. , Duley, P. , Pace, R. M. , White, B. N. , & Frasier, T. (2013). Evidence of a North Atlantic right whale *Eubalaena glacialis* mating ground. Endangered Species Research, 21(1), 55–64. 10.3354/esr00507

[fog12429-bib-0034] Collette, B. B. , & Klein‐MacPhee, G. (2002). Bigelow and Schroeder’s fishes of the Gulf of Maine, third edition (Subsequent ed.). Washington, DC: Smithsonian Books.

[fog12429-bib-0035] Collie, J. S. , Wood, A. D. , & Jeffries, H. P. (2008). Long‐term shifts in the species composition of a coastal fish community. Canadian Journal of Fisheries and Aquatic Sciences, 65(7), 1352–1365. 10.1139/F08-048

[fog12429-bib-0036] Conover, D. O. (1992). Seasonality and the scheduling of life history at different latitudes. Journal of Fish Biology, 41, 161–178. 10.1111/j.1095-8649.1992.tb03876.x

[fog12429-bib-0037] Cooper, R. A. , & Uzmann, J. R. (1971). Migrations and growth of deep‐sea lobsters. Homarus Americanus. Science, 171(3968), 288–290. 10.1126/science.171.3968.288 5538842

[fog12429-bib-0038] Curtin, S. , & Garrod, B. (2008). Vulnerability of marine mammals to diving tourism activities In Garrod B., & Gössling S. (Eds.), New frontiers in marine tourism (pp. 93–113). Amsterdam, the Netherlands: Elsevier.

[fog12429-bib-0039] Curtis, T. H. , Zeeman, S. I. , Summers, E. L. , Cadrin, S. X. , & Skomal, G. B. (2014). Eyes in the sky: Linking satellite oceanography and biotelemetry to explore habitat selection by basking sharks. Animal Biotelemetry, 2, 12 10.1186/2050-3385-2-12

[fog12429-bib-0040] Cushing, D. H. (1969). The regularity of the spawning season of some fishes. ICES Journal of Marine Science, 33(1), 81–92. 10.1093/icesjms/33.1.81

[fog12429-bib-0041] Cushing, D. H. (1990). Plankton production and year‐class strength in fish populations: An update of the match/mismatch hypothesis In Blaxter J. H. S. & Southward A. J. (Eds.), Advances in marine biology (Vol. 26, pp. 249–293). London, UK: Academic Press.

[fog12429-bib-0042] de Keyzer, C. W. , Rafferty, N. E. , Inouye, D. W. , & Thomson, J. D. (2016). Confounding effects of spatial variation on shifts in phenology. Global Change Biology, 23(5), 1783–1791. 10.1111/gcb.13472 27550575

[fog12429-bib-0043] Diamond, A. W. (2012). Managing for migrants: The Gulf of Maine as a global “hotspot” for long‐distance migrants In Stephenson R. L., Annala J. H., Runge J. A., & Hall‐Arber M. (Eds.), Advancing an ecosystem approach in the Gulf of Maine (Vol. 79, pp. 311–320). Bethesda, MD: American Fisheries Society Symposium.

[fog12429-bib-0044] Diamond, A. W. , & Devlin, C. M. (2003). Seabirds as indicators of changes in marine ecosystems: Ecological monitoring on Machias Seal Island. Environmental Monitoring and Assessment, 88(1–3), 153–181. 10.1023/A:1025560805788 14570414

[fog12429-bib-0045] Diaz, R. J. , & Rosenberg, R. (2008). Spreading dead zones and consequences for marine ecosystems. Science, 321(5891), 926–929. 10.1126/science.1156401 18703733

[fog12429-bib-0046] Dijkstra, J. A. , Westerman, E. L. , & Harris, L. G. (2011). The effects of climate change on species composition, succession and phenology: A case study. Global Change Biology, 17(7), 2360–2369. 10.1111/j.1365-2486.2010.02371.x

[fog12429-bib-0047] Doney, S. C. , Ruckelshaus, M. , Emmett Duffy, J. , Barry, J. P. , Chan, F. , English, C. A. , … Talley, L. D. (2012). Climate change impacts on marine ecosystems. Annual Review of Marine Science, 4(1), 11–37. 10.1146/annurev-marine-041911-111611 22457967

[fog12429-bib-0048] Dudley, R. W. , Hodgkins, G. A. , McHale, M. R. , Kolian, M. J. , & Renard, B. (2017). Trends in snowmelt‐related streamflow timing in the conterminous United States. Journal of Hydrology, 547, 208–221. 10.1016/j.jhydrol.2017.01.051

[fog12429-bib-0049] Dufour, F. , Arrizabalaga, H. , Irigoien, X. , & Santiago, J. (2010). Climate impacts on albacore and bluefin tunas migrations phenology and spatial distribution. Progress in Oceanography, 86(1–2), 283–290. 10.1016/j.pocean.2010.04.007

[fog12429-bib-0050] Dunnell, K. L. , & Travers, S. E. (2011). Shifts in the flowering phenology of the northern Great Plains: Patterns over 100 years. American Journal of Botany, 98(6), 935–945. 10.3732/ajb.1000363 21613073

[fog12429-bib-0051] Dunton, K. J. , Chapman, D. , Jordaan, A. , Feldheim, K. , O’Leary, S. J. , McKown, K. A. , & Frisk, M. G. (2012). Genetic mixed‐stock analysis of Atlantic sturgeon *Acipenser oxyrinchus oxyrinchus* in a heavily exploited marine habitat indicates the need for routine genetic monitoring. Journal of Fish Biology, 80(1), 207–217. 10.1111/j.1095-8649.2011.03151.x 22220899

[fog12429-bib-0052] Dunton, K. J. , Jordaan, A. , Conover, D. O. , McKown, K. A. , Bonacci, L. A. , & Frisk, M. G. (2015). Marine distribution and habitat use of Atlantic sturgeon in New York lead to fisheries interactions and bycatch. Marine and Coastal Fisheries, 7(1), 18–32. 10.1080/19425120.2014.986348

[fog12429-bib-0053] Durant, J. M. , Hjermann, D. Ø. , Falkenhaug, T. , Gifford, D. J. , Naustvoll, L. J. , Sullivan, B. K. , … Stenseth, N. C. (2013). Extension of the match‐mismatch hypothesis to predator‐controlled systems. Marine Ecology Progress Series, 474, 43–52. 10.3354/meps10089

[fog12429-bib-0054] Durant, J. M. , Hjermann, D. Ø. , Ottersen, G. , & Stenseth, N. C. (2007). Climate and the match or mismatch between predator requirements and resource availability. Climate Research, 33, 271–283. 10.3354/cr033271

[fog12429-bib-0055] Durbin, E. G. , Campbell, R. G. , Casas, M. C. , Ohman, M. D. , Niehoff, B. , Runge, J. , & Wagner, M. (2003). Interannual variation in phytoplankton blooms and zooplankton productivity and abundance in the Gulf of Maine during winter. Marine Ecology Progress Series, 254, 81–100. 10.3354/meps254081

[fog12429-bib-0056] Durbin, E. G. , Gilman, S. L. , Campbell, R. G. , & Durbin, A. G. (1995). Abundance, biomass, vertical migration and estimated development rate of the copepod *Calanus finmarchicus* in the southern Gulf of Maine during late spring. Continental Shelf Research, 15(4), 571–591. 10.1016/0278-4343(94)00060-Z

[fog12429-bib-0057] Edwards, M. , & Richardson, A. J. (2004). Impact of climate change on marine pelagic phenology and trophic mismatch. Nature, 430(7002), 881–884. 10.1038/nature02808 15318219

[fog12429-bib-0058] Egevang, C. , Stenhouse, I. J. , Phillips, R. A. , Petersen, A. , Fox, J. W. , & Silk, J. R. D. (2010). Tracking of Arctic terns Sterna paradisaea reveals longest animal migration. Proceedings of the National Academy of Sciences of the United States of America, 107(5), 2078–2081. 10.1073/pnas.0909493107 20080662 PMC2836663

[fog12429-bib-0059] Ellis, D. , & Vokoun, J. C. (2009). Earlier spring warming of coastal streams and implications for alewife migration timing. North American Journal of Fisheries Management, 29(6), 1584–1589. 10.1577/M08-181.1

[fog12429-bib-0060] Ennis, G. P. (1984). Small‐scale seasonal movements of the american lobster *Homarus americanus* . Transactions of the American Fisheries Society, 113(3), 336–338. 10.1577/1548-8659(1984)113<336:SSMOTA>2.0.CO;2

[fog12429-bib-0061] Evans, T. G. , Diamond, S. E. , & Kelly, M. W. (2015). Mechanistic species distribution modelling as a link between physiology and conservation. Conservation. Physiology, 3(1), cov056 10.1093/conphys/cov056 PMC4778482 27293739

[fog12429-bib-0062] Facey, D. E. , & Van Den Avyle, M. J. (1987). *Life histories and environmental requirements of coastal fishes and invertebrates (North Atlantic) –American eel* (U.S. Fish and Wildlife Service Biological Report No. 82(11.74), and U.S. Army Corps of Engineers Report No. TR EL‐82‐4) (p. 28). Washington, DC. Retrieved from https://babel.hathitrust.org/cgi/pt?xml:id=mdp.39015086472738;view=1up;seq=3

[fog12429-bib-0063] Fautin, D. , Dalton, P. , Incze, L. S. , Leong, J.‐A. , Pautzke, C. , Rosenberg, A. , … Wolff, N. , 2010). An overview of marine biodiversity in United States waters. PLoS One, 5(8), e11914 10.1371/journal.pone.0011914 20689852 PMC2914028

[fog12429-bib-0064] Fayet, A. L. , Freeman, R. , Anker‐Nilssen, T. , Diamond, A. , Erikstad, K. E. , Fifield, D. , … Guilford, T. (2017). Ocean‐wide drivers of migration strategies and their influence on population breeding performance in a declining seabird. Current Biology, 27(24), 3871–3878.e3. 10.1016/j.cub.2017.11.009 29199078

[fog12429-bib-0065] Ferreira, A. S. , Visser, A. W. , MacKenzie, B. R. , & Payne, M. R. (2014). Accuracy and precision in the calculation of phenology metrics. Journal of Geophysical Research: Oceans, 119(12), 8438–8453. 10.1002/2014JC010323

[fog12429-bib-0066] Findlay, H. S. , Yool, A. , Nodale, M. , & Pitchford, J. W. (2006). Modelling of autumn plankton bloom dynamics. Journal of Plankton Research, 28(2), 209–220. 10.1093/plankt/fbi114

[fog12429-bib-0067] Friedland, K. D. , & Hare, J. A. (2007). Long‐term trends and regime shifts in sea surface temperature on the continental shelf of the northeast United States. Continental Shelf Research, 27(18), 2313–2328. 10.1016/j.csr.2007.06.001

[fog12429-bib-0068] Friedland, K. D. , Leaf, R. T. , Kane, J. , Tommasi, D. , Asch, R. G. , Rebuck, N. , … Saba, V. S. (2015). Spring bloom dynamics and zooplankton biomass response on the US Northeast Continental Shelf. Continental Shelf Research, 102, 47–61. 10.1016/j.csr.2015.04.005

[fog12429-bib-0069] Friedland, K. D. , Manning, J. P. , & Link, J. S. (2012). Thermal phenological factors affecting the survival of Atlantic salmon, *Salmo salar*, in the Gulf of Maine In Stephenson R. L., Annala J. H., Runge J. A., & Hall‐Arber M. (Eds.), Advancing an ecosystem approach in the Gulf of Maine (Vol. 79, pp. 393–407). Bethesda, MD: American Fisheries Society.

[fog12429-bib-0070] Friedland, K. D. , Manning, J. P. , Link, J. S. , Gilbert, J. R. , Gilbert, A. T. , & O’Connell, A. F. (2012). Variation in wind and piscivorous predator fields affecting the survival of Atlantic salmon, *Salmo salar*, in the Gulf of Maine. Fisheries Management and Ecology, 19(1), 22–35. 10.1111/j.1365-2400.2011.00814.x

[fog12429-bib-0071] Friedland, K. D. , Mouw, C. B. , Asch, R. G. , Ferreira, A. S. A. , Henson, S. , Hyde, K. J. W. , … Brady, D. C. (2018). Phenology and time series trends of the dominant seasonal phytoplankton bloom across global scales. Global Ecology and Biogeography, 27(5), 551–569. 10.1111/geb.12717

[fog12429-bib-0072] Friedland, K. D. , Record, N. R. , Asch, R. G. , Kristiansen, T. , Saba, V. S. , Drinkwater, K. F. , … Ji, R. (2016). Seasonal phytoplankton blooms in the North Atlantic linked to the overwintering strategies of copepods. Elementa: Science of the Anthropocene, 4, 99 10.12952/journal.elementa.000099

[fog12429-bib-0073] Friedland, K. D. , Shank, B. V. , Todd, C. D. , McGinnity, P. , & Nye, J. A. (2014). Differential response of continental stock complexes of Atlantic salmon (*Salmo salar*) to the Atlantic Multidecadal Oscillation. Journal of Marine Systems, 133, 77–87. 10.1016/j.jmarsys.2013.03.003

[fog12429-bib-0074] Frumhoff, P. C. , McCarthy, J. J. , Melillo, J. M. , Moser, S. C. , & Wuebbles, D. J. (2007). *Confronting climate change in the US Northeast: Science, impacts, and solutions* . Synthesis Report of the Northeast Climate Impacts Assessment (NECIA). Cambridge, MA: Union of Concerned Scientists (UCS), 160. Retrieved from https://www.ucsusa.org/sites/default/files/legacy/assets/documents/global_warming/pdf/confronting-climate-change-in-the-u-s-northeast.pdf

[fog12429-bib-0075] Gledhill, D. , White, M. , Salisbury, J. , Thomas, H. , Misna, I. , Liebman, M. , … Doney, S. (2015). Ocean and coastal acidification off New England and Nova Scotia. Oceanography, 25(2), 182–197. 10.5670/oceanog.2015.41

[fog12429-bib-0076] Glick, P. , Stein, B. A. , & Edelson, N. A. (Eds.). (2011). Scanning the conservation horizon: A guide to climate change vulnerability assessment. Washington, DC: National Wildlife Federation Retrieved from https://www.nwf.org/~/media/pdfs/global-warming/climate-smart-conservation/nwfscanningtheconservationhorizonfinal92311.ashx

[fog12429-bib-0077] Gobler, C. J. , Doherty, O. M. , Hattenrath‐Lehmann, T. K. , Griffith, A. W. , Kang, Y. , & Litaker, R. W. (2017). Ocean warming since 1982 has expanded the niche of toxic algal blooms in the North Atlantic and North Pacific oceans. Proceedings of the National Academy of Sciences of the United States of America, 114(19), 4975–4980. 10.1073/pnas.1619575114 28439007 PMC5441705

[fog12429-bib-0078] Golet, W. J. , Record, N. R. , Lehuta, S. , Lutcavage, M. , Galuardi, B. , Cooper, A. B. , & Pershing, A. J. (2015). The paradox of the pelagics: Why bluefin tuna can go hungry in a sea of plenty. Marine Ecology Progress Series, 527, 181–192. 10.3354/meps11260

[fog12429-bib-0079] GOMSWG (2016). Gulf of Maine Seabird Working Group 32nd Annual Summer Meeting (p. 48). Bremen, ME: Hog Island Retrieved from http://gomswg.org/minutes.html

[fog12429-bib-0080] Goyert, H. (2014). Relationship among prey availability, habitat, and the foraging behavior, distribution, and abundance of common terns Sterna hirundo and roseate terns *S. dougallii* . Marine Ecology Progress Series, 506, 291–302. 10.3354/meps10834

[fog12429-bib-0081] Griffis, R. , & Howard, J. (2013). Oceans and marine resources in a changing climate. A technical input to the 2013 National Climate Assessment (p. 249). Washington, DC: Island Press.

[fog12429-bib-0082] Groisman, P. Y. , & Knight, R. W. (2008). Prolonged dry episodes over the conterminous United States: New tendencies emerging during the last 40 years. Journal of Climate, 21(9), 1850–1862. 10.1175/2007JCLI2013.1

[fog12429-bib-0083] Gross, M. R. (1987). Evolution of diadromy in fishes In American Fisheries Society Symposium (Vol. 1, pp. 14–25). Retrieved from http://labs.eeb.utoronto.ca/gross/Gross1987.pdf

[fog12429-bib-0084] GSO (2016). *Species identification* . Retrieved from http://www.gsoproject.org/species-identification/

[fog12429-bib-0085] Guilbert, J. , Betts, A. K. , Rizzo, D. M. , Beckage, B. , & Bomblies, A. (2015). Characterization of increased persistence and intensity of precipitation in the northeastern United States: Northeastern precipitation trends. Geophysical Research Letters, 42(6), 1888–1893. 10.1002/2015GL063124

[fog12429-bib-0086] Gulf of Maine Census (2016). *Gulf of Maine register of marine species* . Retrieved from http://www.gulfofmaine-census.org/about-the-gulf/biodiversity-of-the-gulf/lists/gulf-of-maine-register-of-marine-species/

[fog12429-bib-0087] Hall, C. S. , & Kress, S. W. (2004). Comparison of common tern reproductive performance at four restored colonies along the Maine coast, 1991–2002. Waterbirds, 27(4), 424–433. 10.1675/1524-4695(2004)027[0424:COCTRP]2.0.CO;2

[fog12429-bib-0088] Haller, M. A. , Kovacs, K. M. , & Hammill, M. O. (1996). Maternal behaviour and energy investment by grey seals (*Halichoerus grypus*) breeding on land‐fast ice. Canadian Journal of Zoology, 74(8), 1531–1541. 10.1139/z96-167

[fog12429-bib-0089] Halliday, R. G. (1988). Use of seasonal spawning area closures in the management of haddock fisheries in the Northwest Atlantic. NAFO Scientific Council Studies, 12, 27–36. Retrieved from https://archive.nafo.int/open/studies/s12/halliday.pdf

[fog12429-bib-0090] Hamburg, S. P. , Vadeboncoeur, M. A. , Richardson, A. D. , & Bailey, A. S. (2013). Climate change at the ecosystem scale: A 50‐year record in New Hampshire. Climatic Change, 116(3–4), 457–477. 10.1007/s10584-012-0517-2

[fog12429-bib-0091] Hamilton, P. K. , & Mayo, C. A. (1990). *Population characteristics of right whales (Eubalaena glacialis) observed in Cape Cod and Massachusetts Bays, 1978–1986* . Report of the International Whaling Commission Special Issue, 12, 203–208. Retrieved from https://archive.iwc.int/pages/search.php?search=!collection34&bc_from=themes#

[fog12429-bib-0092] Hare, J. A. , Borggaard, D. L. , Friedland, K. D. , Anderson, J. , Burns, P. , Chu, K. , & Saba, V. S. (2016). *Northeast Regional Action Plan – NOAA Fisheries Climate Science Strategy (NOAA Technical Memorandum No. NMFS‐NE‐239)* (p. 94). U.S. Department of Commerce. Retrieved from https://www.nefsc.noaa.gov/program_review/2017-review-SSB/background%20docs/4_NE_Regional_climate_Action_Plan.pdf

[fog12429-bib-0093] Hare, J. A. , Churchill, J. H. , Cowen, R. K. , Berger, T. J. , Cornillon, P. C. , Dragos, P. , … Lee, T. N. (2002). Routes and rates of larval fish transport from the southeast to the northeast United States continental shelf. Limnology and Oceanography, 47(6), 1774–1789. 10.4319/lo.2002.47.6.1774

[fog12429-bib-0094] Hare, J. A. , Morrison, W. E. , Nelson, M. W. , Stachura, M. M. , Teeters, E. J. , Griffis, R. B. , … Griswold, C. A. (2016). A vulnerability assessment of fish and invertebrates to climate change on the Northeast U.S. Continental Shelf. PLOS ONE, 11(2), e0146756 10.1371/journal.pone.0146756 26839967 PMC4739546

[fog12429-bib-0095] Hatch, J. (2002). *Arctic Tern: Sterna paradisaea* . Retrieved from https://birdsna.org/Species-Account/bna/species/arcter/introduction

[fog12429-bib-0096] Hayes, M. L. , Bonaventura, J. , Mitchell, T. P. , Prospero, J. M. , Shinn, E. A. , Van Dolah, F. , & Barber, R. T. (2001). How are climate and marine biological outbreaks functionally linked? Hydrobiologia, 460(1/3), 213–220. 10.1023/A:1013121503937

[fog12429-bib-0097] Hayhoe, K. , Wake, C. P. , Huntington, T. G. , Luo, L. , Schwartz, M. D. , Sheffield, J. , … Wolfe, D. (2007). Past and future changes in climate and hydrological indicators in the US Northeast. Climate Dynamics, 28(4), 381–407. 10.1007/s00382-006-0187-8

[fog12429-bib-0098] Haynes, E. B. , & Wigley, R. L. (1969). Biology of the Northern Shrimp, *Pandalus borealis*, in the Gulf of Maine. Transactions of the American Fisheries Society, 98(1), 60–76. 10.1577/1548-8659(1969)98[60:BOTNSP]2.0.CO;2

[fog12429-bib-0099] Hays, H. , DiCostanzo, J. , Cormons, G. , De Tarso Zuquim Antas, P. , do Nascimento, J. L. X. , de Lima Serrano do Nascimento, I. , & Bremer, R. E. (1997). Recoveries of roseate and common terns in South America. Journal of Field Ornithology, 68, 79–90.

[fog12429-bib-0100] Heller, N. E. , & Zavaleta, E. S. (2009). Biodiversity management in the face of climate change: A review of 22 years of recommendations. Biological Conservation, 142(1), 14–32. 10.1016/j.biocon.2008.10.006

[fog12429-bib-0101] Henderson, M. E. , Mills, K. E. , Thomas, A. C. , Pershing, A. J. , & Nye, J. A. (2017). Effects of spring onset and summer duration on fish species distribution and biomass along the Northeast United States continental shelf. Reviews in Fish Biology and Fisheries, 27(2), 411–424. 10.1007/s11160-017-9487-9.1016/j.biocon.2008.10.006

[fog12429-bib-0102] Hobday, A. J. , Spillman, C. M. , Paige Eveson, J. , & Hartog, J. R. (2016). Seasonal forecasting for decision support in marine fisheries and aquaculture. Fisheries Oceanography, 25, 45–56. 10.1111/fog.12083

[fog12429-bib-0103] Hodgkins, G. A. , & Dudley, R. W. (2005). *Changes in the magnitude of annual and monthly streamflows in New England, 1902–2002 (U.S. Geological Survey Scientific Investigations Report No. SIR2005‐5235)* (p. 37). Reston, VA. Retrieved from https://pubs.er.usgs.gov/publication/sir20055135

[fog12429-bib-0104] Hodgkins, G. A. , & Dudley, R. W. (2006a). Changes in late‐winter snowpack depth, water equivalent, and density in Maine, 1926–2004. Hydrological Processes, 20(4), 741–751. 10.1002/hyp.6111

[fog12429-bib-0105] Hodgkins, G. A. , & Dudley, R. W. (2006b). Changes in the timing of winter–spring streamflows in eastern North America, 1913–2002. Geophysical Research Letters, 33(6), 1–5. 10.1029/2005GL025593

[fog12429-bib-0106] Hodgkins, G. A. , Dudley, R. W. , & Huntington, T. G. (2003). Changes in the timing of high river flows in New England over the 20th Century. Journal of Hydrology, 278(1–4), 244–252. 10.1016/S0022-1694(03)00155-0

[fog12429-bib-0107] Hodgkins, G. A. , Dudley, R. W. , & Huntington, T. G. (2005). Changes in the number and timing of days of ice‐affected flow on Northern New England rivers, 1930–2000. Climatic Change, 71(3), 319–340. 10.1007/s10584-005-5926-z

[fog12429-bib-0108] Holland, G. J. , & Webster, P. J. (2007). Heightened tropical cyclone activity in the North Atlantic: Natural variability or climate trend? Philosophical Transactions of the Royal Society A: Mathematical, Physical and Engineering Sciences, 365(1860), 2695–2716. 10.1098/rsta.2007.2083 17666389

[fog12429-bib-0109] Holligan, P. M. , Balch, W. M. , & Yentsch, C. M. (1984). The significance of subsurface chlorophyll, nitrite and ammonium maxima in relation to nitrogen for phytoplankton growth in stratified waters of the Gulf of Maine. Journal of Marine Research, 42(4), 1051–1073. 10.1357/002224084788520747

[fog12429-bib-0110] Horton, R. M. , Yohe, G. , Easterling, W. , Kates, R. , Ruth, M. , Sussman, E. , … Lipschultz, F. (2014). Chapter 16: Northeast In Melillo J. M., Richmond T. C., & Yohe G. W. (Eds.), Climate change impacts in the United States: The third national climate assessment (pp. 372–395). U.S. Global Change Research Program. Retrieved from http://s3.amazonaws.com/nca2014/high/NCA3_Climate_Change_Impacts_in_the_United%20States_HighRes.pdf

[fog12429-bib-0111] Howell, P. , & Auster, P. J. (2012). Phase shift in an estuarine finfish community associated with warming temperatures. Marine and Coastal Fisheries, 4(1), 481–495. 10.1080/19425120.2012.685144

[fog12429-bib-0112] Howell, S. N. G. (2012). Petrels, albatrosses, and storm‐petrels of North America: A photographic guide. Princeton, NJ: Princeton University Press.

[fog12429-bib-0113] Hunter‐Cevera, K. R. , Neubert, M. G. , Olson, R. J. , Solow, A. R. , Shalapyonok, A. , & Sosik, H. M. (2016). Physiological and ecological drivers of early spring blooms of a coastal phytoplankter. Science, 354(6310), 326–329. 10.1126/science.aaf8536 27846565

[fog12429-bib-0114] Huntington, T. G. , Balch, W. M. , Aiken, G. R. , Sheffield, J. , Luo, L. , Roesler, C. S. , & Camill, P. (2016). Climate change and dissolved organic carbon export to the Gulf of Maine. Journal of Geophysical Research: Biogeosciences, 121(10), 2700–2716. 10.1002/2015JG003314

[fog12429-bib-0115] Huntington, T. G. , & Billmire, M. (2014). Trends in precipitation, runoff, and evapotranspiration for rivers draining to the Gulf of Maine in the United States. Journal of Hydrometeorology, 15(2), 726–743. 10.1175/JHM-D-13-018.1

[fog12429-bib-0116] Huntington, T. G. , Hodgkins, G. A. , & Dudley, R. W. (2003). Historical trend in river ice thickness and coherence in hydroclimatological trends in Maine. Climatic Change, 61(1), 217–236. 10.1023/A:1026360615401

[fog12429-bib-0117] Huntington, T. G. , Hodgkins, G. A. , Keim, B. D. , & Dudley, R. W. (2004). Changes in the proportion of precipitation occurring as snow in New England (1949–2000). Journal of Climate, 17(13), 2626–2636. 10.1175/1520-0442(2004)017<2626:CITPOP>2.0.CO;2

[fog12429-bib-0118] Iafrate, J. , & Oliveira, K. (2008). Factors affecting migration patterns of juvenile river herring in a coastal Massachusetts stream. Environmental Biology of Fishes, 81(1), 101–110. 10.1007/s10641-006-9178-1

[fog12429-bib-0119] ICES . (2017). *Report of the ICES/PICES Workshop on Regional climate change vulnerability assessment for the large marine ecosystems of the northern hemisphere (WKSICCME‐CVA), 19–22 July 2017 (No. ICES CM 2017/SSGEPD:23)* (p. 69). Copenhagen, Denmark: ICES Headquarters. Retrieved from http://ices.dk/sites/pub/Publication%20Reports/Expert%20Group%20Report/SSGEPD/2017/01%20WKSICCME-CVA%20-%20Report%20of%20the%20Workshop%20on%20Regional%20climate%20change%20vulnerability%20assessment%20for%20the%20large%20marine%20ecosystems%20of%20the%20northern%20hemisphere.pdf

[fog12429-bib-0120] Inouye, D. W. (2008). Effects of climate change on phenology, frost damage, and floral abundance of montane wildflowers. Ecology, 89(2), 353–362. 10.1890/06-2128.1 18409425

[fog12429-bib-0121] IPCC . (2007). Climate change 2007: the physical science basis: contribution of Working Group I to the Fourth Assessment Report of the Intergovernmental Panel on Climate Change. (M. L. Parry, Canziani, O. F., Palutikof, J. P., van der Linden, P. J., & Hanson, C. E., Eds.). Cambridge, UK; New York, NY: Cambridge University Press Retrieved from https://www.ipcc.ch/pdf/assessment-report/ar4/wg1/ar4-wg1-frontmatter.pdf

[fog12429-bib-0122] Irigoien, X. , Harris, R. P. , Head, R. N. , & Harbour, D. (2000). North Atlantic Oscillation and spring bloom phytoplankton composition in the English Channel. Journal of Plankton Research, 22(12), 2367–2371. 10.1093/plankt/22.12.2367

[fog12429-bib-0123] Ji, R. , Davis, C. S. , Chen, C. , Townsend, D. W. , Mountain, D. G. , & Beardsley, R. C. (2007). Influence of ocean freshening on shelf phytoplankton dynamics. Geophysical Research Letters, 34(24), L24607 10.1029/2007GL032010

[fog12429-bib-0124] Ji, R. , Davis, C. S. , Chen, C. , Townsend, D. W. , Mountain, D. G. , & Beardsley, R. C. (2008). Modeling the influence of low‐salinity water inflow on winter‐spring phytoplankton dynamics in the Nova Scotian Shelf‐Gulf of Maine region. Journal of Plankton Research, 30(12), 1399–1416. 10.1093/plankt/fbn091

[fog12429-bib-0125] Ji, R. , Edwards, M. , Mackas, D. L. , Runge, J. A. , & Thomas, A. C. (2010). Marine plankton phenology and life history in a changing climate: Current research and future directions. Journal of Plankton Research, 32(10), 1355–1368. 10.1093/plankt/fbq062 20824042 PMC2933132

[fog12429-bib-0126] Johannessen, O. M. , Bengtsson, L. , Miles, M. W. , Kuzmina, S. I. , Semenov, V. A. , Alekseev, G. V. , … Cattle, H. P. (2004). Arctic climate change: Observed and modelled temperature and sea‐ice variability. Tellus A, 56(4), 328–341. 10.1111/j.1600-0870.2004.00060.x

[fog12429-bib-0127] Johnson, C. , Casault, B. , Head, E. , & Spry, J. (2016). *Optical, chemical, and biological oceanographic conditions on the Scotian Shelf and in the eastern Gulf of Maine in 2014* . (DFO Can. Sci. Advis. Sec. Res. Doc. No. 2016/003) (p. v +51). Retrieved from http://waves-vagues.dfo-mpo.gc.ca/Library/362284.pdf

[fog12429-bib-0128] Johnson, C. , Curtis, A. , Pepin, P. , & Runge, J. (2010). *Spatial patterns in zooplankton communities and their seasonal variability in the Northwest Atlantic* . The Atlantic Zone Monitoring Program/Le Programme De Monitorage De La Zone Atlantique AZMP Personnel/Personnel Du PMZA, 9, 27–31. Retrieved fromhttp://citeseerx.ist.psu.edu/viewdoc/download?doi=10.1.1.721.5353&rep=rep1&type=pdf#page=27

[fog12429-bib-0129] Johnson, C. L. , Leising, A. W. , Runge, J. A. , Head, E. J. H. , Pepin, P. , Plourde, S. , & Durbin, E. G. (2008). Characteristics of *Calanus finmarchicus* dormancy patterns in the Northwest Atlantic. ICES Journal of Marine Science, 65(3), 339–350. 10.1093/icesjms/fsm171

[fog12429-bib-0130] Johnson, C. L. , Runge, J. A. , Curtis, K. A. , Durbin, E. G. , Hare, J. A. , Incze, L. S. , … Van Guelpen, L. (2011). Biodiversity and ecosystem function in the Gulf of Maine: Pattern and role of zooplankton and pelagic nekton. PLOS ONE, 6(1), e16491 10.1371/journal.pone.0016491 21304990 PMC3031589

[fog12429-bib-0131] Johnson, J. E. , & Welch, D. J. (2010). Marine Fisheries Management in a changing climate: A review of vulnerability and future options. Reviews in Fisheries Science, 18, 106–124. 10.1080/10641260903434557

[fog12429-bib-0132] Jonsson, B. , & Jonsson, N. (2009). A review of the likely effects of climate change on anadromous Atlantic salmon Salmo salar and brown trout Salmo trutta, with particular reference to water temperature and flow. Journal of Fish Biology, 75(10), 2381–2447. 10.1111/j.1095-8649.2009.02380.x 20738500

[fog12429-bib-0133] Jordaan, A. , Frisk, M. G. , Incze, L. S. , Wolff, N. H. , Hamlin, L. , & Chen, Y. (2012). Multivariate dissemination of species relationships for use in marine spatial planning. Canadian Journal of Fisheries and Aquatic Sciences, 70(2), 316–329. 10.1139/cjfas-2011-0516

[fog12429-bib-0134] Juanes, F. , Gephard, S. , & Beland, K. F. (2004). Long‐term changes in migration timing of adult Atlantic salmon (*Salmo salar*) at the southern edge of the species distribution. Canadian Journal of Fisheries and Aquatic Sciences, 61, 2392–2400. 10.1139/f04-207

[fog12429-bib-0135] Kane, J. (1993). Variability of zooplankton biomass and dominant species abundance on Georges Bank, 1977–1986. Fishery Bulletin, 91, 464–474.

[fog12429-bib-0136] Kane, J. (2007). Zooplankton abundance trends on Georges Bank, 1977–2004. ICES Journal of Marine Science, 64(5), 909–919. 10.1093/icesjms/fsm066

[fog12429-bib-0137] Kane, J. (2009). A comparison of two zooplankton time series data collected in the Gulf of Maine. Journal of Plankton Research, 31(3), 249–259. 10.1093/plankt/fbn119

[fog12429-bib-0138] Kane, J. (2011). Multiyear variability of phytoplankton abundance in the Gulf of Maine. ICES Journal of Marine Science, 68(9), 1833–1841. 10.1093/icesjms/fsr122

[fog12429-bib-0139] Katona, S. K. , & Beard, J. A. (1990). *Population size, migrations and feeding aggregations of the humpback whale (Megaptera novaeangliae) in the western North Atlantic Ocean* . Report of the. International Whaling Commission (Special Issue, 12), 295–306. Retrieved from https://archive.iwc.int/pages/home.php

[fog12429-bib-0140] Kaufman, D. S. , Schneider, D. P. , McKay, N. P. , Ammann, C. M. , Bradley, R. S. , Briffa, K. R. … Arctic Lakes 2k Project Members (2009). Recent warming reverses long‐term arctic cooling. Science, 325(5945), 1236–1239. 10.1126/science.1173983 19729653

[fog12429-bib-0141] Keim, B. D. , Fischer, M. R. , & Wilson, A. M. (2005). Are there spurious precipitation trends in the United States Climate Division database? Geophysical Research Letters, 32(4), 1–3. 10.1029/2004GL021985

[fog12429-bib-0142] Kenney, R. D. , Scott, G. P. , Thompson, T. J. , & Winn, H. E. (1997). Estimates of prey consumption and trophic impacts of cetaceans in the USA northeast continental shelf ecosystem. Journal of Northwest Atlantic Fishery Science, 22, 155–171. 10.2960/J.v22.a13

[fog12429-bib-0143] Keogan, K. , Daunt, F. , Wanless, S. , Phillips, R. A. , Walling, C. A. , Agnew, P. , … Lewis, S. (2018). Global phenological insensitivity to shifting ocean temperatures among seabirds. Nature Climate Change, 8(4), 313–318. 10.1038/s41558-018-0115-z

[fog12429-bib-0144] King, J. R. , Camisa, M. J. , & Manfredi, V. M. (2010). *Massachusetts Division of Marine Fisheries trawl survey effort, lists of species recorded, and bottom temperature trends, 1978‐2007 (Massachusetts Division of Marine Fisheries Technical Report No. TR‐38)* (p. 157). Retrieved from http://www.mass.gov/eea/docs/dfg/dmf/publications/tr-38.pdf

[fog12429-bib-0145] Kleisner, K. M. , Fogarty, M. J. , McGee, S. , Barnett, A. , Fratantoni, P. , Greene, J. , … Pinsky, M. L. (2016). The effects of sub‐regional climate velocity on the distribution and spatial extent of marine species assemblages. PLOS ONE, 11(2), e0149220 10.1371/journal.pone.0149220 26901435 PMC4762943

[fog12429-bib-0146] Kleisner, K. M. , Fogarty, M. J. , McGee, S. , Hare, J. A. , Moret, S. , Perretti, C. T. , & Saba, V. S. (2017). Marine species distribution shifts on the U.S. Northeast Continental Shelf under continued ocean warming. Progress in Oceanography, 153, 24–36. 10.1016/j.pocean.2017.04.001

[fog12429-bib-0147] Klimova, A. , Phillips, C. D. , Fietz, K. , Olsen, M. T. , Harwood, J. , Amos, W. , & Hoffman, J. I. (2014). Global population structure and demographic history of the grey seal. Molecular Ecology, 23(16), 3999–4017. 10.1111/mec.12850 25041117

[fog12429-bib-0148] Koeller, P. , Fuentes‐Yaco, C. , Platt, T. , Sathyendranath, S. , Richards, A. , Ouellet, P. , … Aschan, M. (2009). Basin‐scale coherence in phenology of shrimps and phytoplankton in the North Atlantic Ocean. Science, 324(5928), 791–793. 10.1126/science.1170987 19423827

[fog12429-bib-0149] Kordas, R. L. , Harley, C. D. G. , & O’Connor, M. I. (2011). Community ecology in a warming world: The influence of temperature on interspecific interactions in marine systems. Journal of Experimental Marine Biology and Ecology, 400(1), 218–226. 10.1016/j.jembe.2011.02.029

[fog12429-bib-0150] Kosa, J. T. , & Mather, M. E. (2001). Processes contributing to variability in regional patterns of juvenile river herring abundance across small coastal systems. Transactions of the American Fisheries Society, 130(4), 600–619. 10.1577/1548-8659(2001)130<0600:PCTVIR>2.0.CO;2

[fog12429-bib-0151] Kress, S. W. , Shannon, P. , & O’Neal, C. (2016). Recent changes in the diet and survival of Atlantic puffin chicks in the face of climate change and commercial fishing in midcoast Maine. USA. FACETS, 1(1), 27–43. 10.1139/facets-2015-0009

[fog12429-bib-0152] Kristiansen, T. , Drinkwater, K. F. , Lough, R. G. , & Sundby, S. (2011). Recruitment variability in North Atlantic cod and match‐mismatch dynamics. PLoS ONE, 6(3), e17456 10.1371/journal.pone.0017456 21408215 PMC3049760

[fog12429-bib-0153] Kunkel, K. E. , Karl, T. R. , Brooks, H. , Kossin, J. , Lawrimore, J. H. , Arndt, D. , … Wuebbles, D. (2013). Monitoring and understanding trends in extreme storms: State of knowledge. Bulletin of the American Meteorological Society, 94(4), 499–514. 10.1175/BAMS-D-11-00262.1

[fog12429-bib-0154] Lambert, T. C. (1987). Duration and intensity of spawning in herring *Clupea harengus* as related to the age structure of the mature population. Marine Ecology Progress Series, 39, 209–220. Retrieved from http://www.jstor.org/stable/24825682

[fog12429-bib-0155] Lambert, W. J. (2013). Population biology of an intertidal dorid nudibranch (*Onchidoris muricata*) in the Southern Gulf of Maine, U.S.A.: Changes in phenology due to an invasive prey? American Malacological Bulletin, 31(1), 17–23. 10.4003/006.031.0109

[fog12429-bib-0156] Lambert, W. J. , Bell, G. R. R. , & Harris, L. G. (2016). Growth and reproduction of the dorid nudibranch *Onchidoris muricata* fed native and invasive Bryozoan prey. American Malacological Bulletin, 34(1), 40–50. 10.4003/006.034.0107

[fog12429-bib-0157] Lange, A. M. T. , & Sissenwine, M. P. (1980). Biological considerations relevant to the management of squid (*Loligo pealei* and *Illex illecebrosus*) of the northwest Atlantic. Marine Fisheries Review, 42(7–8), 23–43.

[fog12429-bib-0158] Lesage, V. , & Hammill, M. O. (2001). The status of the grey seal, *Halichoerus grypus*, in the Northwest Atlantic. Canadian Field‐Naturalist, 115(4), 653–662.

[fog12429-bib-0159] Li, Y. , Fratantoni, P. S. , Chen, C. , Hare, J. A. , Sun, Y. , Beardsley, R. C. , & Ji, R. (2015). Spatio‐temporal patterns of stratification on the Northwest Atlantic shelf. Progress in Oceanography, 134, 123–137. 10.1016/j.pocean.2015.01.003

[fog12429-bib-0160] Link, J. S. , Fulton, E. A. , & Gamble, R. J. (2010). The northeast US application of ATLANTIS: A full system model exploring marine ecosystem dynamics in a living marine resource management context. Progress in Oceanography, 87(1–4), 214–234. 10.1016/j.pocean.2010.09.020

[fog12429-bib-0161] Liu , Q. , Xie , S.‐P. , Li , L. , & … N. A. (2005). Ocean thermal advective effect on the annual range of sea surface temperature. Geophysical Research Letters, 32(24), 1–4. 10.1029/2005GL024493

[fog12429-bib-0162] Logan, J. M. , Golet, W. J. , & Lutcavage, M. E. (2015). Diet and condition of Atlantic bluefin tuna (*Thunnus thynnus*) in the Gulf of Maine, 2004–2008. Environmental Biology of Fishes, 98(5), 1411–1430. 10.1007/s10641-014-0368-y

[fog12429-bib-0163] Madsen, T. , & Wilcox, N. (2012). *When it rains, it pours: Global warming and the increase in extreme precipitation from 1948 to 2011* (p. 43). Environment America Research & Policy Center. Retrieved from https://environmentamerica.org/sites/environment/files/reports/When%20It%20Rains%2C%20It%20Pours%20vUS.pdf

[fog12429-bib-0164] Manning, C. , & Bucklin, A. (2005). Multivariate analysis of the copepod community of near‐shore waters in the western Gulf of Maine. Marine Ecology Progress Series, 292, 233–249. 10.3354/meps292233

[fog12429-bib-0165] Maps, F. , Runge, J. A. , Leising, A. , Pershing, A. J. , Record, N. R. , Plourde, S. , & Pierson, J. J. (2012). Modelling the timing and duration of dormancy in populations of *Calanus finmarchicus* from the Northwest Atlantic shelf. Journal of Plankton Research, 34(1), 36–54. 10.1093/plankt/fbr088

[fog12429-bib-0166] Marjadi, M. N. , Roy, A. H. , Jordaan, A. , Gahagan, B. I. , Armstrong, M. , & Whiteley, A. R. (2019). Larger body size and earlier run timing increase alewife reproductive success in a whole lake experiment. Canadian Journal of Fisheries and Aquatic Sciences, 10.1139/cjfas-2017-0451

[fog12429-bib-0167] Martins, E. G. , Hinch, S. G. , Cooke, S. J. , & Patterson, D. A. (2012). Climate effects on growth, phenology, and survival of sockeye salmon (*Oncorhynchus nerka*): A synthesis of the current state of knowledge and future research directions. Reviews in Fish Biology and Fisheries, 22(4), 887–914. 10.1007/s11160-012-9271-9

[fog12429-bib-0168] Massachusetts Energy and Environmental Affairs (2015). *2015 Final Massachusetts Ocean Management Plan, January 2015* . Retrieved from http://www.mass.gov/eea/waste-mgnt-recycling/coasts-and-oceans/mass-ocean-plan/2015-final-ocean-plan.html

[fog12429-bib-0169] Mather, F. J. (1995). *Historical document: Life history and fisheries of Atlantic bluefin tuna* . NOAA Tech. Memo. SEFC, 370, 1–165. Retrieved from https://repository.library.noaa.gov/view/noaa/8461

[fog12429-bib-0170] Matthiessen, G. C. , & Toner, R. C. (1963). A study of the marine resources of Barnstable County, Massachusetts (p. 114). Edgartown, MA: Marine Research Foundation Inc..

[fog12429-bib-0171] Mayo, C. A. , & Marx, M. K. (1990). Surface foraging behaviour of the North Atlantic right whale, *Eubalaena glacialis*, and associated zooplankton characteristics. Canadian Journal of Zoology, 68(10), 2214–2220. 10.1139/z90-308

[fog12429-bib-0172] Mayor, S. J. , Guralnick, R. P. , Tingley, M. W. , Otegui, J. , Withey, J. C. , Elmendorf, S. C. , … Schneider, D. C. (2017). Increasing phenological asynchrony between spring green‐up and arrival of migratory birds. Scientific Reports, 7(1), 1–10. 10.1038/s41598-017-02045-z 28507323 PMC5432526

[fog12429-bib-0173] Meister, A. L. (1962). Atlantic salmon production in Cove Brook, Maine. Transactions of the American Fisheries Society, 91(2), 208–212. 10.1577/1548-8659(1962)91[208:ASPICB]2.0.CO;2

[fog12429-bib-0174] Melnychuk, M. C. , Dunton, K. J. , Jordaan, A. , McKown, K. A. , & Frisk, M. G. (2017). Informing conservation strategies for the endangered Atlantic sturgeon using acoustic telemetry and multi‐state mark‐recapture models. Journal of Applied Ecology, 54(3), 914–925. 10.1111/1365-2664.12799

[fog12429-bib-0175] MERCINA (2004). Supply‐side ecology and the response of zooplankton to climate‐driven changes in North Atlantic Ocean circulation. Oceanography, 17(3), 60–71. 10.5670/oceanog.2004.31

[fog12429-bib-0176] Methratta, E. T. , & Link, J. S. (2007). Ontogenetic variation in habitat associations for four flatfish species in the Gulf of Maine‐Georges Bank region. Journal of Fish Biology, 70(6), 1669–1688. 10.1111/j.1095-8649.2007.01428.x

[fog12429-bib-0177] Meyer, J. L. , Tate, C. M. , Edwards, R. T. , & Crocker, M. T. (1988). The trophic significance of dissolved organic carbon in streams In Swank W. T., & Crossley D. A. (Eds.), Forest hydrology and ecology at coweeta (Vol. 66, pp. 269–278). New York, NY: Springer New York.

[fog12429-bib-0178] Meyer‐Gutbrod, E. L. , & Greene, C. H. (2018). Uncertain recovery of the North Atlantic right whale in a changing ocean. Global Change Biology, 24(1), 455–464. 10.1111/gcb.13929 29084379

[fog12429-bib-0179] Miller‐Rushing, A. J. , Høye, T. T. , Inouye, D. W. , & Post, E. (2010). The effects of phenological mismatches on demography. Philosophical Transactions of the Royal Society of London B: Biological Sciences, 365(1555), 3177–3186. 10.1098/rstb.2010.0148 20819811 PMC2981949

[fog12429-bib-0180] Miller‐Rushing, A. J. , Inouye, D. W. , & Primack, R. B. (2008). How well do first flowering dates measure plant responses to climate change? The effects of population size and sampling frequency. Journal of Ecology, 96(6), 1289–1296. 10.1111/j.1365-2745.2008.01436.x

[fog12429-bib-0181] Miller‐Rushing, A. J. , Lloyd‐Evans, T. L. , Primack, R. B. , & Satzinger, P. (2008). Bird migration times, climate change, and changing population sizes. Global Change Biology, 14(9), 1959–1972. 10.1111/j.1365-2486.2008.01619.x

[fog12429-bib-0182] Mills, K. , Pershing, A. , Brown, C. , Chen, Y. , Chiang, F.‐S. , Holland, D. , … Wahle, R. (2013). Fisheries management in a changing climate: Lessons from the 2012 ocean heat wave in the Northwest Atlantic. Oceanography, 26(2), 191–195. 10.5670/oceanog.2013.27

[fog12429-bib-0183] Mills, K. E. , Pershing, A. J. , & Hernández, C. M. (2017). Forecasting the seasonal timing of Maine’s Lobster fishery. Frontiers in Marine Science, 4, 1–10. 10.3389/fmars.2017.00337

[fog12429-bib-0184] Murison, L. D. , & Gaskin, D. E. (1989). The distribution of right whales and zooplankton in the Bay of Fundy. Canada. Canadian Journal of Zoology, 67(6), 1411–1420. 10.1139/z89-200

[fog12429-bib-0185] NCEI (2017). *National Centers for Environmental Information (NCEI) [Text]* . Retrieved from https://www.ncei.noaa.gov/node

[fog12429-bib-0187] Nelson, E. J. , Kareiva, P. , Ruckelshaus, M. , Arkema, K. , Geller, G. , Girvetz, E. , … Tallis, H. (2013). Climate change’s impact on key ecosystem services and the human well‐being they support in the US. Frontiers in Ecology and the Environment, 11(9), 483–893. 10.1890/120312

[fog12429-bib-0188] Nelson, G. A. , & Ross, M. R. (1991). Biology and population changes of Northern sand lance (*Ammodytes dubius*) from the Gulf of Maine to the Middle Atlantic Bight. Journal of Northwest Atlantic Fishery Science, 11, 11–27.

[fog12429-bib-0189] Newsome, D. , & Rodger, K. (2008). Impacts of tourism on pinnipeds and implications for tourism management In Higham J., & Lück M. (Eds.), Marine wildlife and tourism management: Insights from the natural and social sciences (pp. 182–205). Wallingford, UK: CABI.

[fog12429-bib-0190] Nisbet, I. C. T. (1984). Migration and winter quarters of North American roseate terns as shown by banding recoveries. Journal of Field Ornithology, 55, 1–142.

[fog12429-bib-0191] NMFS (2008). Endangered fish and wildlife; Final rule to implement speed restrictions to reduce the threat of ship collisions with North Atlantic right whales. Federal Register, 73(198), 60173–60191.

[fog12429-bib-0192] NMFS (2013). Endangered fish and wildlife; Final rule to remove the sunset provision of the final rule implementing vessel speed restrictions to reduce the threat of ship collisions with North Atlantic right whales. Federal Register, 78(236), 73726–73736.

[fog12429-bib-0193] Northeast Fish and Wildlife Diversity Technical Committee . (2016). *Northeast state wildlife action plan database* . [Online database]. Retrieved from https://rcngrants.org/

[fog12429-bib-0194] Northeast Regional Planning Body (2016). *Northeast Ocean Plan [document]* . Retrieved from https://neoceanplanning.org/plan/

[fog12429-bib-0195] Nye, J. , Link, J. , Hare, J. , & Overholtz, W. (2009). Changing spatial distribution of fish stocks in relation to climate and population size on the Northeast United States continental shelf. Marine Ecology Progress Series, 393, 111–129. 10.3354/meps08220gressSeries

[fog12429-bib-0196] O’Reilly, J. E. , & Zetlin, C. (1998). *Seasonal, horizontal, and vertical distribution of phytoplankton chlorophyll a in the Northeast U.S. Continental Shelf Ecosystem (NOAA Technical Report, NMFS No. 139)* (p. 119). U.S. Department of Commerce. Retrieved from http://spo.nwr.noaa.gov/tr139opt.pdf

[fog12429-bib-0197] Ohlberger, J. , Thackeray, S. J. , Winfield, I. J. , Maberly, S. C. , & Vollestad, L. A. (2014). When phenology matters: Age‐size truncation alters population response to trophic mismatch. Proceedings of the Royal Society B: Biological Sciences, 281(1793), 20140938 10.1098/rspb.2014.0938 PMC4173671 25165767

[fog12429-bib-0198] Otero, J. , L’Abée‐Lund, J. H. , Castro‐Santos, T. , Leonardsson, K. , Storvik, G. O. , Jonsson, B. , … Vøllestad, L. A. (2014). Basin‐scale phenology and effects of climate variability on global timing of initial seaward migration of Atlantic salmon (*Salmo salar*). Global Change Biology, 20(1), 61–75. 10.1111/gcb.12363 23966281

[fog12429-bib-0199] Overholtz, W. J. , & Link, J. S. (2007). Consumption impacts by marine mammals, fish, and seabirds on the Gulf of Maine‐Georges Bank Atlantic herring (*Clupea harengus*) complex during the years 1977–2002. ICES Journal of Marine Science: Journal Du Conseil, 64(1), 83–96.

[fog12429-bib-0200] Pace, R. M. , Corkeron, P. J. , & Kraus, S. D. (2017). State‐space mark‐recapture estimates reveal a recent decline in abundance of North Atlantic right whales. Ecology and Evolution, 7, 8730–8741. 10.1002/ece3.3406 29152173 PMC5677501

[fog12429-bib-0201] Pacifici, M. , Foden, W. B. , Visconti, P. , Watson, J. E. M. , Butchart, S. H. M. , Kovacs, K. M. , … Rondinini, C. (2015). Assessing species vulnerability to climate change. Nature Climate Change, 5(3), 215–224. 10.1038/nclimate2448

[fog12429-bib-0202] Parmesan, C. (2006). Ecological and evolutionary responses to recent climate change. Annual Review of Ecology, Evolution, and Systematics, 37(1), 637–669. 10.1146/annurev.ecolsys.37.091305.110100

[fog12429-bib-0203] Parmesan, C. (2007). Influences of species, latitudes and methodologies on estimates of phenological response to global warming. Global Change Biology, 13(9), 1860–1872. 10.1111/j.1365-2486.2007.01404.x

[fog12429-bib-0204] Parmesan, C. , & Yohe, G. (2003). A globally coherent fingerprint of climate change impacts across natural systems. Nature, 421(6918), 37–42. 10.1038/nature01286 12511946

[fog12429-bib-0205] Payne, M. R. , Hobday, A. J. , MacKenzie, B. R. , Tommasi, D. , Dempsey, D. P. , Fässler, S. M. M. , … Villarino, E. (2017). Lessons from the first generation of marine ecological forecast products. Frontiers in Marine Science, 4, 1–15. 10.3389/fmars.2017.00289

[fog12429-bib-0206] Payne, P. M. , Wiley, D. N. , Pittman, S. , Clapham, P. J. , & Jossi, J. W. (1990). Recent fluctuations in the abundance of baleen whales in the southern Gulf of Maine in relation to changes in selected prey. Fishery Bulletin, 88, 687–696.

[fog12429-bib-0207] Pearse, W. D. , Davis, C. C. , Inouye, D. W. , Primack, R. B. , & Davies, T. J. (2017). A statistical estimator for determining the limits of contemporary and historic phenology. Nature Ecology & Evolution, 1(12), 1876 10.1038/s41559-017-0350-0 29109468

[fog12429-bib-0208] Peck, M. A. , Huebert, K. B. , & Llopiz, J. K. (2012). Intrinsic and extrinsic factors driving match–mismatch dynamics during the early life history of marine fishes In Woodward G., Jacob U. & O’Gorman E. J. (Eds.), Advances in ecological research (Vol. 47, pp. 177–302). London, UK: Academic Press.

[fog12429-bib-0209] Peer, A. C. , & Miller, T. J. (2014). Climate change, migration phenology, and fisheries management interact with unanticipated consequences. North American Journal of Fisheries Management, 34(1), 94–110. 10.1080/02755947.2013.847877

[fog12429-bib-0210] Pendleton, D. , Pershing, A. , Brown, M. , Mayo, C. , Kenney, R. , Record, N. , & Cole, T. (2009). Regional‐scale mean copepod concentration indicates relative abundance of North Atlantic right whales. Marine Ecology Progress Series, 378, 211–225. 10.3354/meps07832

[fog12429-bib-0211] Pershing, A. J. , Alexander, M. A. , Hernandez, C. M. , Kerr, L. A. , Le Bris, A. , Mills, K. E. , … Thomas, A. C. (2015). Slow adaptation in the face of rapid warming leads to collapse of the Gulf of Maine cod fishery. Science, 350(6262), 809–812. 10.1126/science.aac9819 26516197

[fog12429-bib-0212] Pershing, A. , Greene, C. , Jossi, J. , Obrien, L. , Brodziak, J. , & Bailey, B. (2005). Interdecadal variability in the Gulf of Maine zooplankton community, with potential impacts on fish recruitment. ICES Journal of Marine Science, 62(7), 1511–1523. 10.1016/j.icesjms.2005.04.025

[fog12429-bib-0213] Petitgas, P. , Rijnsdorp, A. D. , Dickey‐Collas, M. , Engelhard, G. H. , Peck, M. A. , Pinnegar, J. K. , … Nash, R. D. M. (2013). Impacts of climate change on the complex life cycles of fish. Fisheries Oceanography, 22(2), 121–139. 10.1111/fog.12010

[fog12429-bib-0214] Pettigrew, N. R. , Churchill, J. H. , Janzen, C. D. , Mangum, L. J. , Signell, R. P. , Thomas, A. C. , … Xue, H. (2005). The kinematic and hydrographic structure of the Gulf of Maine Coastal Current. Deep Sea Research Part II: Topical Studies in Oceanography, 52(19–21), 2369–2391. 10.1016/j.dsr2.2005.06.033

[fog12429-bib-0215] Pierson, J. J. , Batchelder, H. , Saumweber, W. , Leising, A. , & Runge, J. (2013). The impact of increasing temperatures on dormancy duration in *Calanus finmarchicus* . Journal of Plankton Research, 35(3), 504–512. 10.1093/plankt/fbt022

[fog12429-bib-0216] Pinsky, M. L. , & Fogarty, M. (2012). Lagged social‐ecological responses to climate and range shifts in fisheries. Climatic Change, 115(3–4), 883–891. 10.1007/s10584-012-0599-x

[fog12429-bib-0217] Pinsky, M. L. , Worm, B. , Fogarty, M. J. , Sarmiento, J. L. , & Levin, S. A. (2013). Marine taxa track local climate velocities. Science, 341(6151), 1239–1242. 10.1126/science.1239352 24031017

[fog12429-bib-0218] Platt, T. , Fuentes‐Yaco, C. , & Frank, K. T. (2003). Marine ecology: Spring algal bloom and larval fish survival. Nature, 423(6938), 398–399. 10.1038/423398b 12761538

[fog12429-bib-0219] Platt, T. , Sathyendranath, S. , White, G. N. , Fuentes‐Yaco, C. , Zhai, L. , Devred, E. , & Tang, C. (2010). Diagnostic properties of phytoplankton time series from remote sensing. Estuaries and Coasts, 33(2), 428–439. 10.1007/s12237-009-9161-0

[fog12429-bib-0220] Pollet, I. L. , Hedd, A. , Taylor, P. D. , Montevecchi, W. A. , & Shutler, D. (2014). Migratory movements and wintering areas of Leach’s storm‐petrels tracked using geolocators: Migration and wintering areas of Leach’s storm‐petrels. Journal of Field Ornithology, 85(3), 321–328. 10.1111/jofo.12071

[fog12429-bib-0221] Poloczanska, E. S. , Brown, C. J. , Sydeman, W. J. , Kiessling, W. , Schoeman, D. S. , Moore, P. J. , … Richardson, A. J. (2013). Global imprint of climate change on marine life. Nature Climate Change, 3(10), 919–925. 10.1038/nclimate1958

[fog12429-bib-0222] Post, E. , Pedersen, C. , Wilmers, C. C. , & Forchhammer, M. C. (2008). Warming, plant phenology and the spatial dimension of trophic mismatch for large herbivores. Proceedings of the Royal Society B: Biological Sciences, 275(1646), 2005–2013. 10.1098/rspb.2008.0463 PMC2596357 18495618

[fog12429-bib-0223] Powell, E. J. , Tyrrell, M. C. , Milliken, A. , Tirpak, J. M. , & Staudinger, M. D. (2017). A synthesis of thresholds for focal species along the U.S. Atlantic and Gulf Coasts: A review of research and applications. Ocean & Coastal Management, 148, 75–88. 10.1016/j.ocecoaman.2017.07.012

[fog12429-bib-0224] Powers, K. D. (1983). *Pelagic distributions of marine birds off the northeastern United States (NOAA Technical Memorandum No. NMFS‐F/NEC‐27)* (p. 201). US Department of Commerce. Retrieved from http://nefsc.noaa.gov/nefsc/publications/tm/pdfs/tmfnec27.pdf

[fog12429-bib-0225] Powers, K. D. , & Backus, E. H. (1987). Energy transfer to birds In Backus R. H., & Bourne D. W. (Eds.), Georges bank (pp. 372–375). Cambridge, MA: MIT Press.

[fog12429-bib-0226] Powers, K. , Wiley, D. , Allyn, A. , Welch, L. , & Ronconi, R. (2017). Movements and foraging habitats of great shearwaters *Puffinus gravis* in the Gulf of Maine. Marine Ecology Progress Series, 574, 211–226. 10.3354/meps12168

[fog12429-bib-0227] Primack, R. B. , Ibáñez, I. , Higuchi, H. , Lee, S. D. , Miller‐Rushing, A. J. , Wilson, A. M. , & Silander, J. A. (2009). Spatial and interspecific variability in phenological responses to warming temperatures. Biological Conservation, 142(11), 2569–2577. 10.1016/j.biocon.2009.06.003

[fog12429-bib-0228] Pringle, J. D. , & Burke, D. L. (1993). The Canadian lobster fishery and its management, with emphasis on the Scotian Shelf and the Gulf of Maine In Parsons L. S., & Lear W. H. (Eds.), Perspectives on Canadian marine fisheries management (Vol. 226, pp. 91–122). Canadian Bulletin of Fisheries and Aquatic Sciences. Retrieved from http://www.nrcresearchpress.com/doi/abs/10.1139/9780660150031#page=98

[fog12429-bib-0229] Quinn, T. P. , & Adams, D. J. (1996). Environmental changes affecting the migratory timing of American shad and sockeye salmon. Ecology, 77, 1151–1162.

[fog12429-bib-0230] Ramp, C. , Delarue, J. , Palsbøll, P. J. , Sears, R. , & Hammond, P. S. (2015). Adapting to a warmer ocean—Seasonal shift of baleen whale movements over three decades. PLoS ONE, 10(3), e0121374 10.1371/journal.pone.0121374 25785462 PMC4364899

[fog12429-bib-0231] Ramp, S. R. , Schlitz, R. J. , & Wright, W. R. (1985). The Deep flow through the Northeast Channel, Gulf of Maine. Journal of Physical Oceanography, 15(12), 1790–1808. 10.1175/1520-0485(1985)015<1790:TDFTTN>2.0.CO;2

[fog12429-bib-0232] Record , N. R. , Balch , W. M. , & Stamieszkin , K. (2018). Century‐scale changes in phytoplankton phenology in the Gulf of Maine. PeerJ, 6, e27425v1 10.7287/peerj.preprints.27425v1 PMC6500720 31106049

[fog12429-bib-0233] Record, N. R. , O’Brien, J. D. , Stamieszkin, K. , & Runge, J. A. (2016). Omic‐style statistical clustering reveals old and new patterns in the Gulf of Maine ecosystem. Canadian Journal of Fisheries and Aquatic Sciences, 00, 1–7. 10.1139/cjfas-2016-0151

[fog12429-bib-0234] Record, N. R. , Pershing, A. J. , & Jossi, J. W. (2010). Biodiversity as a dynamic variable in the Gulf of Maine continuous plankton recorder transect. Journal of Plankton Research, 32(12), 1675–1684. 10.1093/plankt/fbq050

[fog12429-bib-0235] Renkawitz, M. D. , Sheehan, T. F. , Dixon, H. J. , & Nygaard, R. (2015). Changing trophic structure and energy dynamics in the Northwest Atlantic: Implications for Atlantic salmon feeding at West Greenland. Marine Ecology Progress Series, 538, 197–211. 10.3354/meps11470

[fog12429-bib-0236] Rhode Island Ocean Special Area Management Plan (2010). *Rhode Island ocean special area management plan: Volume 1 [webpage]* . Retrieved from https://www.mass.gov/service-details/massachusetts-ocean-management-plan

[fog12429-bib-0237] Richards, R. A. (2012). Phenological shifts in hatch timing of northern shrimp Pandalus borealis. Marine Ecology Progress Series, 456, 149–158. 10.3354/meps09717

[fog12429-bib-0238] Richards, R. A. , O’Reilly, J. E. , & Hyde, K. J. W. (2016). Use of satellite data to identify critical periods for early life survival of northern shrimp in the Gulf of Maine. Fisheries Oceanography, 25(3), 306–319. 10.1111/fog.12153

[fog12429-bib-0239] Richards, R. A. , Whitmore, K. , Fischer, J. , Hunter, M. , Waine, M. , & Drew, K. (2012). *Assessment report for Gulf of Maine northern shrimp – 2012* . Atlantic States Marine Fisheries Commission’s Northern Shrimp Technical Committee. Retrieved from http://www.asmfc.org/uploads/file/2012NorthernShrimpAssessment.pdf

[fog12429-bib-0240] Richardson, D. E. , Hare, J. A. , Overholtz, W. J. , & Johnson, D. L. (2010). Development of long‐term larval indices for Atlantic herring (*Clupea harengus*) on the northeast US continental shelf. ICES Journal of Marine Science, 67(4), 617–627. 10.1093/icesjms/fsp276

[fog12429-bib-0241] Richardson, D. E. , Palmer, M. C. , & Smith, B. E. (2014). The influence of forage fish abundance on the aggregation of Gulf of Maine Atlantic cod (*Gadus morhua*) and their catchability in the fishery. Canadian Journal of Fisheries and Aquatic Sciences, 71(9), 1349–1362. 10.1139/cjfas-2013-0489

[fog12429-bib-0242] Richaud, B. , Kwon, Y.‐O. , Joyce, T. M. , Fratantoni, P. S. , & Lentz, S. J. (2016). Surface and bottom temperature and salinity climatology along the continental shelf off the Canadian and U.S. East Coasts. Continental Shelf Research, 124, 165–181. 10.1016/j.csr.2016.06.005

[fog12429-bib-0243] Robards, M. D. , Willson, M. F. , Armstrong, R. H. , & Piatt, J. F. (2000). *Sand lance: a review of biology and predator relations and annotated bibliography. (No. PNW‐RP‐521)* . Portland, OR: U.S. Department of Agriculture, Forest Service, Pacific Northwest Research Station. 10.2737/PNW-RP-521

[fog12429-bib-0244] Rosset, J. , Roy, A. H. , Gahagan, B. I. , Whiteley, A. R. , Armstrong, M. P. , Sheppard, J. J. , & Jordaan, A. (2017). Temporal patterns of migration and spawning of river herring in coastal Massachusetts. Transactions of the American Fisheries Society, 146, 1101–1114. 10.1080/00028487.2017.1341851

[fog12429-bib-0245] Runge, J. A. , Ji, R. , Thompson, C. R. S. , Record, N. R. , Chen, C. , Vandemark, D. C. , … Maps, F. (2015). Persistence of *Calanus finmarchicus* in the western Gulf of Maine during recent extreme warming. Journal of Plankton Research, 37(1), 221–232. 10.1093/plankt/fbu098

[fog12429-bib-0246] Rupp, R. S. (1959). Variation in the life history of the American smelt in inland waters of Maine. Transactions of the American Fisheries Society, 88(4), 241–252. 10.1577/1548-8659(1959)88[241:VITLHO]2.0.CO;2

[fog12429-bib-0247] Ryan, K. , Danylchuk, A. , & Jordaan, A. (2018). Is marine spatial planning enough to overcome biological data deficiencies? Journal of Environmental Assessment Policy and Management, 20(4), 1850012 10.1142/S1464333218500126

[fog12429-bib-0248] Saba, V. S. , Griffies, S. M. , Anderson, W. G. , Winton, M. , Alexander, M. A. , Delworth, T. L. , … Zhang, R. (2016). Enhanced warming of the Northwest Atlantic Ocean under climate change. Journal of Geophysical Research: Oceans, 121(1), 118–132. 10.1002/2015JC011346

[fog12429-bib-0249] Sagarese, S. R. , Frisk, M. G. , Cerrato, R. M. , Sosebee, K. A. , Musick, J. A. , & Rago, P. J. (2014). Application of generalized additive models to examine ontogenetic and seasonal distributions of spiny dogfish (Squalus acanthias) in the Northeast (US) shelf large marine ecosystem. Canadian Journal of Fisheries and Aquatic Sciences, 71(6), 847–877. 10.1139/cjfas-2013-0342

[fog12429-bib-0250] Saunders, R. , Hachey, M. A. , & Fay, C. W. (2006). Maine’s diadromous fish community: Past, present, and implications for Atlantic Salmon recovery. Fisheries, 31(11), 537–547. 10.1577/1548-8446(2006)31[537:MDFC]2.0.CO;2

[fog12429-bib-0251] Scattergood, L. W. (1952). The northern shrimp fishery of Maine. Commercial Fisheries Review, 14, 1–16. Retrieved from https://www.biodiversitylibrary.org/item/99540#page/3/mode/1up

[fog12429-bib-0252] Schevill, W. E. , Watkins, W. A. , & Moore, K. E. (1986). *Status of Eubalaena glacialis off Cape Cod* . Rep. Int. Whal. Comm., Special, (10), 79–82. Retrieved from https://archive.iwc.int/pages/search.php?search=%21collection34&k=

[fog12429-bib-0253] Schlünz, B. , & Schneider, R. R. (2000). Transport of terrestrial organic carbon to the oceans by rivers: Re‐estimating flux‐ and burial rates. International Journal of Earth Sciences, 88(4), 599–606. 10.1007/s005310050290

[fog12429-bib-0254] Shearman, R. K. , & Lentz, S. J. (2010). Long‐term sea surface temperature variability along the U.S. East Coast. Journal of Physical Oceanography, 40(5), 1004–1017. 10.1175/2009JPO4300.1

[fog12429-bib-0255] Sherman, S. A. , Stepanek, K. L. , Pierce, F. , Tetrault, R. , & O’Donnell, C. (2015). *Annual Report on the Maine‐New Hampshire Inshore Trawl Survey January 1, 2015‐December 31, 2015 (NOAA Fisheries Northeast Region Cooperative Research Partners Program Contract No. NA13NMF4720104 (DMR #3025))* (p. 138). Retrieved from http://www.maine.gov/dmr/science-research/projects/trawlsurvey/index.html

[fog12429-bib-0256] Siders, Z. A. , Westgate, A. J. , Johnston, D. W. , Murison, L. D. , & Koopman, H. N. (2013). Seasonal variation in the spatial distribution of basking sharks (*Cetorhinus maximus*) in the Lower Bay of Fundy, Canada. PLoS ONE, 8(12), e82074 10.1371/journal.pone.0082074 24324747 PMC3852988

[fog12429-bib-0257] Sissenwine, M. P. , & Shepherd, J. G. (1987). An alternative perspective on recruitment overfishing and biological reference points. Canadian Journal of Fisheries and Aquatic Sciences, 44(4), 913–918. 10.1139/f87-110

[fog12429-bib-0258] Smith, P. C. , Pettigrew, N. R. , Yeats, P. , Townsend, D. W. , & Han, G. (2012). Regime shift in the Gulf of Maine In American Fisheries Society Symposium (Vol. 79, pp. 185–203). Retrieved from http://grampus.umeoce.maine.edu/dave/Smith-et-al-2012-RegimeShift.pdf

[fog12429-bib-0259] Song, H. , Ji, R. , Stock, C. , & Wang, Z. (2010). Phenology of phytoplankton blooms in the Nova Scotian Shelf‐Gulf of Maine region: Remote sensing and modeling analysis. Journal of Plankton Research, 32(11), 1485–1499. 10.1093/plankt/fbq086

[fog12429-bib-0260] Staudinger, M. D. , Carter, S. L. , Cross, M. S. , Dubois, N. S. , Duffy, J. E. , Enquist, C. , … Turner, W. (2013). Biodiversity in a changing climate: A synthesis of current and projected trends in the US. Frontiers in Ecology and the Environment, 11(9), 465–473. 10.1890/120272

[fog12429-bib-0261] Staudinger, M. D. , Morelli, T. L. , & Bryan, A. M. (2015). *Integrating climate change into Northeast and Midwest State Wildlife Action Plans* Northeast Climate Science Center. Retrieved from http://necsc.umass.edu

[fog12429-bib-0262] Stein, B. A. , Staudt, A. , Cross, M. S. , Dubois, N. S. , Enquist, C. , Griffis, R. , … Pairis, A. (2013). Preparing for and managing change: Climate adaptation for biodiversity and ecosystems. Frontiers in Ecology and the Environment, 11(9), 502–510. 10.1890/120277

[fog12429-bib-0263] Stevenson, D. K. , & Scott, M. L. (2005). *Essential fish habitat source document: Atlantic herring, Clupea harengus, life history and habitat characteristics (2nd edition) (NOAA Technical Memorandum No. NMFS‐NE‐192)* (p. 84). U.S. Department of Commerce. Retrieved from https://www.nefsc.noaa.gov/publications/tm/tm192/tm192.pdf

[fog12429-bib-0264] Stevick, P. T. , Allen, J. , Clapham, P. J. , Katona, S. K. , Larsen, F. , Lien, J. , … Hammond, P. S. (2006). Population spatial structuring on the feeding grounds in North Atlantic humpback whales (*Megaptera novaeangliae*). Journal of Zoology, 270(2), 244–255. 10.1111/j.1469-7998.2006.00128.x

[fog12429-bib-0265] Thackeray, S. J. , Sparks, T. H. , Frederiksen, M. , Burthe, S. , Bacon, P. J. , Bell, J. R. , … Wanless, S. (2010). Trophic level asynchrony in rates of phenological change for marine, freshwater and terrestrial environments. Global Change Biology, 16(12), 3304–3313. 10.1111/j.1365-2486.2010.02165.x

[fog12429-bib-0266] Thakur, K. K. , Revie, C. , Stryhn, H. , Tibbetts, S. S. , Lavallée, J. , & Vanderstichel, R. (2017). Risk factors associated with soft‐shelled lobsters (*Homarus americanus*) in southwestern Nova Scotia, Canada. FACETS, 2(1), 15–33. 10.1139/facets-2016-0038

[fog12429-bib-0267] The Chronicle Herald . (2015, September 22). *Right whales off Cape Breton going the wrong way for shipping, fishing* . Retrieved from http://thechronicleherald.ca/novascotia/1312707-right-whales-off-cape-breton-going-the-wrong-way-for-shipping-fishing

[fog12429-bib-0268] Thibeault, J. M. , & Seth, A. (2014). Changing climate extremes in the Northeast United States: Observations and projections from CMIP5. Climatic Change, 127(2), 273–287. 10.1007/s10584-014-1257-2

[fog12429-bib-0269] Thomas, A. C. , Pershing, A. J. , Friedland, K. D. , Nye, J. A. , Mills, K. E. , Alexander, M. A. , … Henderson, M. E. (2017). Seasonal trends and phenology shifts in sea surface temperature on the North American northeastern continental shelf. Elem Sci Anth, 5, 48 10.1525/elementa.240

[fog12429-bib-0270] Thomas, A. C. , Townsend, D. W. , & Weatherbee, R. (2003). Satellite‐measured phytoplankton variability in the Gulf of Maine. Continental Shelf Research, 23(10), 971–989. 10.1016/S0278-4343(03)00086-4

[fog12429-bib-0271] Thomas, K. A. , Fornwall, M. D. , Weltzin, J. F. , & Griffis, R. B. (2014). Organization of marine phenology data in support of planning and conservation in ocean and coastal ecosystems. Ecological Informatics, 24, 169–176. 10.1016/j.ecoinf.2014.08.007

[fog12429-bib-0272] Thompson, B. C. , Jackson, J. A. , Burger, J. , Hill, L. A. , Kirsch, E. M. , & Atwood, J. L. (1997). Least tern (*Sterna antillarum*). The Birds of North America Online, 10.2173/bna.290

[fog12429-bib-0273] Tian, R. , Chen, C. , Qi, J. , Ji, R. , Beardsley, R. C. , & Davis, C. (2015). Model study of nutrient and phytoplankton dynamics in the Gulf of Maine: Patterns and drivers for seasonal and interannual variability. ICES Journal of Marine Science, 72(2), 388–402. 10.1093/icesjms/fsu090

[fog12429-bib-0274] Tillotson, M. D. , & Quinn, T. P. (2018). Selection on the timing of migration and breeding: A neglected aspect of fishing‐induced evolution and trait change. Fish and Fisheries, 19(1), 170–181. 10.1111/faf.12248

[fog12429-bib-0275] Townsend, D. W. (1983). The relations between larval fishes and zooplankton in two inshore areas of the Gulf of Maine. Journal of Plankton Research, 5(2), 145–173. 10.1093/plankt/5.2.145

[fog12429-bib-0276] Townsend, D. W. (1984). Comparison of inshore zooplankton and ichthyoplankton populations of the Gulf of Maine. Marine Ecology Progress Series. Oldendorf, 15(1), 79–90. 10.3354/meps015079

[fog12429-bib-0277] Townsend, D. W. , Cammen, L. M. , Holligan, P. M. , Campbell, D. E. , & Pettigrew, N. R. (1994). Causes and consequences of variability in the timing of spring phytoplankton blooms. Deep Sea Research Part I: Oceanographic Research Papers, 41(5–6), 747–765. 10.1016/0967-0637(94)90075-2

[fog12429-bib-0278] Townsend, D. W. , & Spinrad, R. W. (1986). Early spring phytoplankton blooms in the Gulf of Maine. Continental Shelf Research, 6(4), 515–529. 10.1016/0278-4343(86)90021-X

[fog12429-bib-0279] True, E. D. , & Wiitala, S. A. (1990). Annual temperature curves in twelve regions of the Gulf of Maine. NAFO Science Council Studies, 14, 21–27. Retrieved from http://archive.nafo.int/open/studies/s14/true.pdf

[fog12429-bib-0280] Trzcinski, M. K. , Devred, E. , Platt, T. , & Sathyendranath, S. (2013). Variation in ocean colour may help predict cod and haddock recruitment. Marine Ecology Progress Series, 491, 187–197. 10.3354/meps10451

[fog12429-bib-0281] Tupper, M. H. , Anthony, V. C. , Chenoweth, S. B. , & MacCluen, H. A. (1998). Biology and assessment of Gulf of Maine herring stocks (p. 104). Portland, ME: Gulf of Maine Aquarium Retrieved from https://www.researchgate.net/publication/286368676_Biology_and_assessment_of_the_Gulf_of_Maine_herring_stocks

[fog12429-bib-0282] USA National Phenology Network (2018). *USA National Phenology Network [webpage]* . Retrieved from https://usanpn.org/

[fog12429-bib-0283] Veit, R. R. , & Petersen, W. R. (1993). Birds of Massachusetts. Lincoln, MA: Massachusetts Audubon Society.

[fog12429-bib-0284] Vermeer, K. , Szabo, I. , & Greisman, P. (1987). The relationship between plankton‐feeding Bonaparte’s and Mew gulls and tidal upwelling at Active Pass, British Columbia. Journal of Plankton Research, 9(3), 483–501. 10.1093/plankt/9.3.483

[fog12429-bib-0285] Vincent, L. a. , Zhang, X. , Brown, R. d. , Feng, Y. , Mekis, E. , Milewska, E. j. , … Wang, X. l. (2015). Observed trends in Canada’s climate and influence of low‐frequency variability modes. Journal of Climate, 28(11), 4545–4560. 10.1175/JCLI-D-14-00697.1

[fog12429-bib-0286] Volk, C. J. , Volk, C. B. , & Kaplan, L. A. (1997). Chemical composition of biodegradable dissolved organic matter in streamwater. Limnology and Oceanography, 42(1), 39–44. 10.4319/lo.1997.42.1.0039

[fog12429-bib-0287] Walsh, H. J. , Richardson, D. E. , Marancik, K. E. , & Hare, J. A. (2015). Long‐term changes in the distributions of larval and adult fish in the Northeast U.S. Shelf ecosystem. PLoS ONE, 10(9), e0137382 10.1371/journal.pone.0137382 26398900 PMC4580593

[fog12429-bib-0288] Walters, C. J. (1986). Adaptive Management of Renewable Resources. Basingstoke, UK: Macmillan Publishers Ltd Retrieved from http://pure.iiasa.ac.at/2752/

[fog12429-bib-0289] Whidden, S. E. (2016). *Patterns of Natal Recruitment in the Atlantic Puffin (Fratercula arctica)* . University of New Brunswick. Retrieved from http://www.unb.ca/research/alar/_resources/pdf/alar-thesis/sewhidden_msc_thesis_final.pdf

[fog12429-bib-0290] Wiebe, K. L. , & Gerstmar, H. (2010). Influence of spring temperatures and individual traits on reproductive timing and success in a migratory woodpecker. The Auk, 127(4), 917–925. 10.1525/auk.2010.10025

[fog12429-bib-0291] Wilson, R. J. , Banas, N. S. , Heath, M. R. , & Speirs, D. C. (2016). Projected impacts of 21st century climate change on diapause in *Calanus finmarchicus* . Global Change Biology, 22(10), 3332–3340. 10.1111/gcb.13282 26990351

[fog12429-bib-0292] Wilson, S. G. , Lutcavage, M. E. , Brill, R. W. , Genovese, M. P. , Cooper, A. B. , & Everly, A. W. (2005). Movements of bluefin tuna (*Thunnus thynnus*) in the northwestern Atlantic Ocean recorded by pop‐up satellite archival tags. Marine Biology, 146(2), 409–423. 10.1007/s00227-004-1445-0

[fog12429-bib-0293] Winn, H. E. , Price, C. A. , & Sorensen, P. W. (1986). *The distributional biology of the right whale (Eubalaena glacialis) in the western North Atlantic* . Report of the International Whaling Commission Special Issue, 10, 129–138. Retrieved from https://experts.umn.edu/en/publications/the-distributional-biology-of-the-right-whale-eubalaena-glacialis

[fog12429-bib-0294] Wood, A. J. M. , Collie, J. S. , & Hare, J. A. (2009). A comparison between warm‐water fish assemblages of Narragansett Bay and those of Long Island Sound waters. Fishery Bulletin, 107(1), 89–100. http://fishbull.noaa.gov/1071/wood2.pdf

[fog12429-bib-0295] Wood, E. M. , & Kellermann, J. L. (2015). Phenological synchrony and bird migration: Changing climate and seasonal resources in North America. Boca Raton, FL: CRC Press Retrieved from http://www.crcnetbase.com/doi/pdfplus/10.1201/b18011-19

[fog12429-bib-0296] Wuenschel, M. J. , Able, K. W. , Buckel, J. A. , Morley, J. W. , Lankford, T. , Branson, A. c. , … Stormer, D. (2012). Recruitment patterns and habitat use of young‐of‐the‐year bluefish along the United States East Coast: Insights from coordinated coastwide sampling. Reviews in Fisheries Science, 20(2), 80–102. 10.1080/10641262.2012.660999

[fog12429-bib-0297] Wurtzell, K. V. , Baukus, A. , Brown, C. J. , Jech, J. M. , Pershing, A. J. , & Sherwood, G. D. (2016). Industry‐based acoustic survey of Atlantic herring distribution and spawning dynamics in coastal Maine waters. Fisheries Research, 178, 71–81. 10.1016/j.fishres.2015.11.011

[fog12429-bib-0298] Yako, L. A. , Mather, M. E. , & Juanes, F. (2002). Mechanisms for migration of anadromous herring: An ecological basis for effective conservation. Ecological Applications, 12(2), 521–534. 10.1890/1051-0761(2002)012[0521:MFMOAH]2.0.CO;2

[fog12429-bib-0299] Yakola, K. , & Brofsky, I. (2016). *Seal Island National Wildlife Refuge 2016 Season Report (May 2 – August 8)* (p. 47). National Audubon Society, Ithaca New York. Retrieved from https://www.audubon.org/

[fog12429-bib-0300] Zhai, L. , Platt, T. , Tang, C. , Sathyendranath, S. , & Hernández Walls, R. (2011). Phytoplankton phenology on the Scotian Shelf. ICES Journal of Marine Science, 68(4), 781–791. 10.1093/icesjms/fsq175

