# Supplementary material for: It’s about time: A synthesis of changing phenology in the Gulf of Maine ecosystem
Source: Fish Oceanogr. 2019 Apr 22;28(5):532–66. doi: 10.1111/fog.12429 (PMC6774335; doi:10.1111/fog.12429)
Supplement: Supplementary file 1 [file FOG-28-532-s001.pdf]

Appendix 1: Quantitative and detailed baseline phenology data from the Gulf of Maine ecosystem. Information is organized by functional groups, species, sub-region and season.

Functional Group	Species	Gulf of Maine Region	Winter	Spring	Summer	Fall	References
Phytoplankton		Central and eastern Gulf, Scotian Shelf, Massachusetts and Cape Cod Bays		Pronounced spring bloom in offshore surface waters. Average bloom initiation timing varies between day 67-90 across the Scotian Shelf and Slope waters		Relatively small bloom (compared to spring) in offshore waters	O'Reilly & Zetlin, 1998; Keller et al., 2001; Platt et al., 2010; Zhai et al., 2011
Phytoplankton		Scotian Shelf		Spring bloom on the eastern and western Scotian Shelf are short in duration but high in amplitude on average, while blooms in the slope and Gulf Stream waters are longer in duration but smaller in amplitude			DFO, 2015
Phytoplankton		Prince-5 time series station in the outer Bay of Fundy			Spring bloom initiation is later and duration is longer than on the Scotian Shelf; peaks occur in June and August-September		Johnson et al., 2016
Zooplankton	Mesozooplankton	Central Scotian Shelf, Halifax-2 time series station	Minimum biomass and abundance January-February	Maximum biomass and abundance in April			Johnson et al., 2016
Zooplankton	Mesozooplankton		Low species diversity			High species diversity	Record et al., 2010
Zooplankton	Mesozooplankton	Outer Bay of Fundy, Prince-5 time series station	Minimum biomass/abundance in January-May. Coastal influence is evident at Prince-5 with strong pulses of barnacle larvae in late winter-early spring		Maximum biomass/abundance in July-September		Johnson et al., 2010; Johnson et al., 2016
Zooplankton	*Calanus finmarchicus*	Central Gulf, Central Scotian Shelf, Halifax-2 time series station	Develop from diapausing fifth copepodite stages to adults in early January	Emergence from depth; first generation in the spring.	Peak abundance in late spring/early summer; smaller second generation in late summer	Late stage individuals enter diapause; annual minimum in surface waters	Pershing et al., 2005; Johnson et al., 2010; Johnson et al., 2016
Zooplankton	*Calanus finmarchicus*	Northwest Atlantic			Mean diapause duration is 200 days with onset in late summer		Durbin et al., 2000; Johnson et al., 2008; Melle et al., 2014;
Zooplankton	*Centropages hamatus*	Georges Bank			*Centropages hamatus* dominates biomass		Kane, 1993; Manning & Buckling, 2005

Functional Group	Species	Gulf of Maine Region	Winter	Spring	Summer	Fall	References
Zooplankton	*Calanus finmarchicus, Temora longicornis, Centropages typicus*	Coastal waters of central and western Gulf of Maine		Peak abundance; *Calanus finmarchicu* s dominates in the early spring	*Temora longicorni* s dominates biomass from late July to early August	*Centropages typicus* dominates from approximately October to January	Kane, 1993; Manning & Buckling, 2005
Zooplankton	*Centropages* spp; *Pseudocalanus* spp; *C. finmarchicus*	Cape Cod Bay	Centropages spp. dominate during early winter and Pseudocalanus spp. during late winter.	C. finmarchicus and Pseudocalanus spp. dominate during early spring. Sluggish currents allow for retention and aggregation of plankton.		Centropages spp. dominate during the fall.	Conversi et al., 2001; Bessinger et al., 2003; MERCINA, 2004; DeLorenzo Costa et al., 2006; Greene and Pershing, 2007; Jiang et al., 2007; Kane, 2007; Leeney et al., 2009; Pendleton et al., 2009
Zooplankton	*Sagitta elegans*	Coastal waters of GOM	Adult individuals dominate the population and are evenly distributed along the coast of Maine		Peak abundance, dominated by immature individuals	Population concentrates to the southwest	Sherman & Schaner 1968; Pettigrew et al., 2005
Macro-invertebrates	Longfin squid *(Doryteuthis pealeii)*		Offshore migration	Late spring, early summer spawning and cohort	Inshore migration	Late summer, early fall spawning and cohort	Black et al., 1987; Jacobson, 2005
Macro-invertebrates	Northern shortfin squid (*Illex illecebrosus*)		Spawning in southern latitudes	Primarily occur offshore	Migrate north via the Gulf Stream and occur in more inshore waters	Migration offshore and out of the GOM	Black et al., 1987; Hendrickson & Holmes, 2004
Macro-invertebrates	American lobster *(Homarus americanus)*			Molt and move from deep offshore waters into shallow coastal areas			Cooper & Uzmann, 1971; Aiken 1980; Dunnington et al., 2005; Glenn & Pugh, 2006; Mills et al., 2013
Macro-invertebrates	Acorn barnacle (*Semibalanus balanoides*)	Bay of Fundy			Reproduction is timed with adults developing immature oocytes between May-June; by late July-early September sperm are produced to fertilize brooded embryos		Bouchard & Aiken 2012
Macro-invertebrates	Northern shrimp *(Pandalus borealis)*	western Gulf of Maine	Females migrate near shore to hatch brood, mature males begin sex transition	Larval development in inshore waters, females migrate back offshore	Postlarvae settle to benthos	Mating and spawning in offshore waters	Shumway et al., 1985
Fish	Multiple ichthyoplankton species	Coastal waters of central and western Gulf of Maine		Peak abundance of larvae, March-April to May-June, depending on species and latitude (earlier in south compared to north)			Townsend, 1983; Townsend, 1984
Fish	Atlantic herring (*Clupea harengus*)	Georges Banks and coastal habitats				Spawning occurs between October-November close to shore	Richardson et al, 2010; Wurtzell et al., 2016

Functional Group	Species	Gulf of Maine Region	Winter	Spring	Summer	Fall	References
Fish	Alewife (*Alosa pseudoharengus*)	Coastal and inland freshwater habitats		Adult spawning migration is initiated when water temperatures are between 5-10 ºC. Spawning is initiated at temperatures of 10-22 ºC			Rounsefell, & Stringer, 1945; Loesch, 1987
Fish	Blueback herring (*Alosa aestivalis*)	Coastal and inland freshwater habitats		Adult spawning migration overlaps with alewife but is generally initiated 3-4 weeks later. Peak spawning occurs about 2-3 weeks after alewife			Collette & Klein-MacPhee, 2002; Saunders et al., 2006; Alexander et al., 2017
Fish	Lamprey (*Petromyzon marinus*)	Coastal and inland freshwater habitats		Highly variable timing of movements into spawning habitats, likely influenced by pheromone cues, which can be affected by fluctuating flow conditions			Hogg et al., 2013
Fish	Haddock (*Melanogrammus aeglefinus*)	Scotian Shelf, Georges Bank		Earlier spring primary production blooms support larger larval recruitment events and higher fish survival index		Size, location and timing of fall primary production bloom the year prior to spawning affects the quantity and/or quality of gametes and is associated with magnitude of recruitment events	Platt et al., 2003; Friedland et al., 2008; Trzcinski et al. 2013; Leaf & Friedland, 2014; Friedland et al. 2015
Fish	Bluefin tuna (*Thunnus thynnus*)				Post spawning bluefin tuna migrate to temperate regions to forage on prey with high energy densities. Bluefin tuna migrations coincide with the locations and timing of Atlantic herring (Clupea harengus) in the GOM, which have been shown through diet studies to be a major prey in teh northwest Atlantic shelf.	Bluefin tuna remain in the GOM until November and then migrate out to other Atlantic habitats	Crane, 1936; Lawson et al., 1998; Chase, 2002; Pleizier et al., 2012; Logan et al., 2015
Elasmobranch	Basking shark *(Cetorhinus maximus)*	Cape Cod Bay, Great South Channel, Bay of Fundy			Migrate into region; distribution particularly in the Bay of Fundy is affected by prey availability, bathymetry, SST, and chlorophyll-a		Campana et al., 2008; Gore et al., 2008; Siders et al., 2013; Curtis et al., 2014

Functional Group	Species	Gulf of Maine Region	Winter	Spring	Summer	Fall	References
Marine mammal	Atlantic Right Whale *(Eubalaena glacialis)*	Cape Cod Bay & Great South Channel	Arrival in Cape Cod Bay around January or February	Peak abundance of whales (by number of animals sighted) in March or April	Peak number of right whales occurs in the Great South Channel May through July		Gaskin, 1987; Murison & Gaskin, 1989; Brown et al., 1995; Kenney et al., 1995; Mayo et al., 2018
Marine mammal	Atlantic Right Whale *(Eubalaena glacialis)*	Bay of Fundy, Scotian Shelf & Deep basins	Known to occur on Outer Falls in December and January, but survey coverage is poor	Known to occur on Jeffreys Ledge, but survey coverage is poor	Between early to mid-summer and mid-fall the majority of sightings occur in the Bay of Fundy and on the Nova Scotian Shelf, especially Roseway Basin		Gaskin, 1987; Murison & Gaskin, 1989; Brown et al., 1995; Kenney et al., 1995
Marine mammal	Grey seal (*Halichoerus grypus)*	Scotian Shelf and Georges Bank; Sable Island, Gulf of St. Lawrence	Breeding at haul out sites during January	The Sable Island stock has a post-breeding pelagic phase from February until April, and return to Sable Island for their spring molt	Foraging	Foraging increases to prepare for the upcoming breeding season	Lesage & Hammill, 2001; Lesage et al., 2001; Boehme et al., 2012; Klimova et al., 2014;
Marine mammal	Harbor seal *(Phoca vitulina)*		Preference for ice-free waters compels harbor seals to move to Southern New England and Maine coastal waters in winter and spring.				Baechler et al., 2002; Gilbert et al., 2005; Waring et al., 2006; Bowen et al., 2007; Waring et al., 2010;
Seabird	Roseate, common, and arctic terns: (*Sterna dougallii, S. hirundo, S. paradisaea*)			Breeding	Chick rearing: Common tern first and peak hatch dates on Seal and Stratton Islands occurred approximately June 20, and 28th; earliest hatches were June 15 and 24 between 1991-2002		Hall and Kress 2004
Seabird	Shearwaters (Procellariidae)	Georges Banks, Bay of Fundy		Arrival: Great shearwaters arrive on Georges Banks last week of May and into June	Departure of Great shearwaters starting in August for breeding isalnds in South Atlantic or young birds moving up to Bay of Fundy.	Most birds reaching spots south of the equator by end of September; Fall migration out of GOM completed by mid-November	Nisbet et al., 2013
Seabird	Gulls (Laridae)			Energetic plankton feeding occurs typically in March in New England, which can last for weeks, through April			Vermeer et al., 1987
Seabird	Auks (Alcidae): Atlantic Puffin (*Fratercula arctica*); Razorbill (*Alca torda*); Common Murre (*Uria aalge*)			Arrive late April / early May, lay single egg mid to late May	Puffins, razorbills and murres hatch eggs, raise chicks	Auk chicks leave nest and go out to sea, late July (razorbills, murres) through late August (puffins)	Diamond & Devlin, 2003

Appendix 1 Literature Cited

Aiken, D. E. (1980). Molting and growth. In J. S. Cobb & B. F. Phillips (Eds.), *The Biology and Management of Lobsters. Vol. I: Physiology and Behavior*. (pp. 91–163). New York: Academic Press. Retrieved from https://books.google.com/books?hl=en&lr=&id=9ude00zGRiEC&oi=fnd&pg=PA91&dq=Molting+and+growth.+Aiken&ots=JOjTgY33ob&sig=KF7dAWl4cY_xUzfzCPttL6FCLN4#v=onepage&q=Molting%20and%20growth.%20Aiken&f=false

Alexander, K. E., Leavenworth, W. B., Willis, T. V., Hall, C., Mattocks, S., Bittner, S. M., … Jordaan, A. (2017). Tambora and the mackerel year: Phenology and fisheries during an extreme climate event. *Science Advances*, *3*(1), e1601635. https://doi.org/10.1126/sciadv.1601635

Baechler, J., Beck, C. A., & Bowen, W. D. (2002). Dive shapes reveal temporal changes in the foraging behaviour of different age and sex classes of harbour seals (*Phoca vitulina*). *Canadian Journal of Zoology*, *80*(9), 1569–1577. https://doi.org/10.1139/z02-150

Bessinger, M. K., Mayo, C. A., & Brown, M. W. (2003). *Using zooplankton enumeration to manage the Cape Cod Bay right whale critical habitat-2003*. Provincetown, MA: Center for Coastal Studies. Retrieved from http://www.coastalstudies.org/pdf/population2.pdf

Black, G. A. P., Rowell, T. W., & Dawe, E. G. (1987). *Atlas of the biology and distribution of the squids Illex illecebrosus and Loligo pealei in the Northwest Atlantic*. Ottawa, Ont: Dept. of Fisheries and Oceans. Retrieved from http://www.dfo-mpo.gc.ca/Library/106107.pdf

Boehme, L., Thompson, D., Fedak, M., Bowen, D., Hammill, M. O., & Stenson, G. B. (2012). How many seals were there? The global shelf loss during the last glacial maximum and its effect on the size and distribution of grey seal populations. *PLoS ONE*, *7*(12), e53000. https://doi.org/10.1371/journal.pone.0053000

Bouchard, G. M., & Aiken, R. B. (2012). Latitudinal variation in the reproductive cycle and size of the northern rock barnacle *Semibalanus balanoides* (L.) (Cirripedia, Archaeobalanidae) in the Bay of Fundy. *Crustaceana*, *85*(7), 779–787. https://doi.org/10.1163/156854012X650214

Bowen, W. D., McMillan, J. I., & Blanchard, W. (2007). Reduced population growth of gray

seals at Sable Island: Evidence from pup production and age of primiparity. *Marine Mammal Science*, *23*(1), 48–64. https://doi.org/10.1111/j.1748-7692.2006.00085.x

Brown, M. W., Allen, J. M., & Kraus, S. D. (1995). The designation of seasonal right whale conservation areas in the waters of Atlantic Canada. In N. L. Shackell & J. H. M. Willison (Eds.), *Marine protected areas and sustainable fisheries.* (pp. 90–98). Science and Management of Marine Protected Areas Association, Wolfville, NS. https://scholar.google.com/scholar?hl=en&as_sdt=0%2C22&q=The+designation+of+seasonal+right+whale+conservation+areas+in+the+waters+of+Atlantic+Canada.&btnG=

Campana, S. E., Gibson, J., Brazner, J., Marks, L., Joyce, W., Gosselin, J.-F., … Lawson, J. (2008). *Status of basking sharks in Atlantic Canada* (No. 2008/004) (p. 61). Canadian Science Advisory Secretariat. Retrieved from https://www.researchgate.net/profile/Steven_Campana/publication/268388877_Status_of_Basking_Sharks_in_Atlantic_Canada/links/546a30f20cf2397f78301415/Status-of-Basking-Sharks-in-Atlantic-Canada.pdf

Chase, B. C. (2002). Differences in diet of Atlantic bluefin tuna (*Thunnus thynnus*) at five seasonal feeding grounds on the New England continental shelf. *Fishery Bulletin*, *100*(2), 168–180. Retrieved from http://aquaticcommons.org/15201/

Collette, B. B., & Klein-MacPhee, G. (2002). *Bigelow and Schroeder's Fishes of the Gulf of Maine, Third Edition* (Subsequent edition). Washington, DC: Smithsonian Books. Retrieved from
https://scholar.google.com/scholar?hl=en&as_sdt=0%2C22&q=Bigelow+and+Schroeder%E2%80%99s+Fishes+of+the+Gulf+of+Maine%2C+Third+Edition&btnG=

Conversi, A., Piontkovski, S., & Hameed, S. (2001). Seasonal and interannual dynamics of *Calanus finmarchicus* in the Gulf of Maine (Northeastern US shelf) with reference to the North Atlantic Oscillation. *Deep Sea Research Part II: Topical Studies in Oceanography*, *48*(1–3), 519–530. https://doi.org/10.1016/S0967-0645(00)00088-6

Cooper, R. A., & Uzmann, J. R. (1971). Migrations and growth of deep-sea lobsters, *Homarus americanus*. *Science (New York, N.Y.)*, *171*(3968), 288–290. https://doi.org/10.1126/science.171.3968.288

Crane, J. (1936). Notes on the biology and ecology of giant tuna, *Thunnus thynnus* Linnaeus, observed at Portland, Maine. *Zoologica : Scientific Contributions of the New York*

Zoological Society., *21*(16), 207--212. Retrieved from https://scholar.google.com/scholar?hl=en&as_sdt=0%2C22&q=Notes+on+the+biology+and+ecology+of+giant+tuna%2C+Thunnus+thynnus+Linnaeus%2C+observed+at+Portland%2C+Maine.&btnG=

Curtis, T. H., Zeeman, S. I., Summers, E. L., Cadrin, S. X., & Skomal, G. B. (2014). Eyes in the sky: Linking satellite oceanography and biotelemetry to explore habitat selection by basking sharks. *Animal Biotelemetry*, *2*(1), 12. https://doi.org/10.1186/2050-3385-2-12

DeLorenzo Costa, A., Durbin, E. G., & Mayo, C. A. (2006). Variability in the nutritional value of the major copepods in Cape Cod Bay (Massachusetts, USA) with implications for right whales. *Marine Ecology*, *27*(2), 109–123. https://doi.org/10.1111/j.1439-0485.2006.00087.x

DFO. (2015). *Oceanographic conditions in the Atlantic zone in 2014* (Science Advisory Report No. 2015/030). Canadian Science Advisory Secretariat. Retrieved from http://www.dfo-mpo.gc.ca/csas-sccs/Publications/SAR-AS/2015/2015_030-eng.html

Diamond, A. W., & Devlin, C. M. (2003). Seabirds as indicators of changes in marine ecosystems: Ecological monitoring on Machias Seal Island. *Environmental Monitoring and Assessment*, *88*(1–3), 153–181. https://doi.org/10.1023/A:1025560805788

Dunnington, M. J., Wahle, R. A., Bell, M. C., & Geraldi, N. R. (2005). Evaluating local population dynamics of the American lobster, *Homarus americanus*, with trap-based mark-recapture methods and seabed mapping. *New Zealand Journal of Marine and Freshwater Research*, *39*(6), 1253–1276. https://doi.org/10.1080/00288330.2005.9517391

Durbin, E., Garrahan, P. R., & Casas, M. C. (2000). Abundance and distribution of *Calanus finmarchicus* on the Georges Bank during 1995 and 1996. *ICES Journal of Marine Science*, *57*(6), 1664–1685. https://doi.org/10.1006/jmsc.2000.0974

Friedland, K. D., Hare, J. A., Wood, G. B., Col, L. A., Buckley, L. J., Mountain, D. G., … Pilskaln, C. H. (2008). Does the fall phytoplankton bloom control recruitment of Georges Bank haddock, *Melanogrammus aeglefinus*, through parental condition? *Canadian Journal of Fisheries and Aquatic Sciences*, *65*(6), 1076–1086. https://doi.org/10.1139/F08-040

Friedland, K. D., Leaf, R. T., Kristiansen, T., & Large, S. I. (2015). Layered effects of parental

condition and larval survival on the recruitment of neighboring haddock stocks. *Canadian Journal of Fisheries and Aquatic Sciences*, *72*(11), 1672–1681. https://doi.org/10.1139/cjfas-2015-0084

Gaskin, D. E. (1987). Updated status of the right whale, *Eubalaena glacialis*, in Canada. *Canadian Field-Naturalist Ottawa ON*, *101*(2), 295–309. https://www.biodiversitylibrary.org/item/89248#page/5/mode/1up

Gilbert, J. R., Waring, G. T., Wynne, K. M., & Guldager, N. (2005). Changes in abundance of harbor seals in Maine, 1981–2001. *Marine Mammal Science*, *21*(3), 519–535. https://doi.org/10.1111/j.1748-7692.2005.tb01246.x

Glenn, R. P., & Pugh, T. L. (2006). Epizootic shell disease in American lobster (*Homarus americanus*) in Massachusetts coastal waters: Interactions of temperature, maturity, and intermolt duration. *Journal of Crustacean Biology*, *26*(4), 639–645. https://doi.org/10.1651/S-2754.1

Gore, M. A., Rowat, D., Hall, J., Gell, F. R., & Ormond, R. F. (2008). Transatlantic migration and deep mid-ocean diving by basking shark. *Biology Letters*, *4*(4), 395–398. https://doi.org/10.1098/rsbl.2008.0147

Greene, C. H., & Pershing, A. J. (2007). OCEANS: Climate drives sea change. *Science*, *315*(5815), 1084–1085. https://doi.org/10.1126/science.1136495

Hall, C. S., & Kress, S. W. (2004). Comparison of Common tern reproductive performance at four restored colonies along the Maine coast, 1991-2002. *Waterbirds*, *27*(4), 424–433. https://doi.org/10.1675/1524-4695(2004)027[0424:COCTRP]2.0.CO;2

Hendrickson, L. C., & Holmes, E. M. (2004). *Essential fish habitat source document: Northern Shortfin squid, Illex illecebrosus, life history and habitat characteristics. Second Edition* (NOAA Technical Memorandum No. NMFS-NE-191) (p. 36). U.S. Department of Commerce. Retrieved from https://www.nefsc.noaa.gov/publications/tm/tm191/tm191.pdf

Hogg, R., Coghlan, S. M., & Zydlewski, J. (2013). Anadromous sea lampreys recolonize a Maine coastal river tributary after dam removal. *Transactions of the American Fisheries Society*, *142*(5), 1381–1394. https://doi.org/10.1080/00028487.2013.811103

Jacobson, L. D. (2005). *Essential fish habitat source document: Longfin Inshore Squid, Loligo pealeii, life history and habitat characteristics. Second Edition* (NOAA Technical

Memorandum No. NMFS-NE-193) (p. 42). U.S. Department of Commerce. Retrieved from https://www.nefsc.noaa.gov/publications/tm/tm193/tm193.pdf

Jiang, M., Wallace, G. T., Zhou, M., Libby, S., & Hunt, C. D. (2007). Summer formation of a high-nutrient low-oxygen pool in Cape Cod Bay, USA. *Journal of Geophysical Research*, *112*(C5). https://doi.org/10.1029/2006JC003889

Johnson, C., Casault, B., Head, E., & Spry, J. (2016). *Optical, chemical, and biological oceanographic conditions on the Scotian Shelf and in the eastern Gulf of Maine in 2014.* (DFO Can. Sci. Advis. Sec. Res. Doc. No. 2016/003) (p. *v* +51). Retrieved from http://waves-vagues.dfo-mpo.gc.ca/Library/362284.pdf

Johnson, C., Curtis, A., Pepin, P., & Runge, J. (2010). Spatial patterns in zooplankton communities and their seasonal variability in the Northwest Atlantic. *The Atlantic Zone Monitoring Program/Le Programme de Monitorage de La Zone Atlantique AZMP Personnel/Personnel Du PMZA*, *9*, 27–31. Retrieved from http://citeseerx.ist.psu.edu/viewdoc/download?doi=10.1.1.721.5353&rep=rep1&type=pdf#page=27

Johnson, C. L., Leising, A. W., Runge, J. A., Head, E. J. H., Pepin, P., Plourde, S., & Durbin, E. G. (2008). Characteristics of *Calanus finmarchicus* dormancy patterns in the Northwest Atlantic. *ICES Journal of Marine Science*, *65*(3), 339–350. https://doi.org/10.1093/icesjms/fsm171

Kane, J. (1993). Variability of zooplankton biomass and dominant species abundance on Georges Bank, 1977-1986. *Fishery Bulletin*, *91*, 464–474. Retrieved from http://fishbull.noaa.gov/913/kane.pdf

Kane, J. (2007). Zooplankton abundance trends on Georges Bank, 1977–2004. *ICES Journal of Marine Science*, *64*(5), 909–919. https://doi.org/10.1093/icesjms/fsm066

Keller, A. A., Taylor, C., Oviatt, C., Dorrington, T., Holcombe, G., & Reed, L. (2001). Phytoplankton production patterns in Massachusetts Bay and the absence of the 1998 winter–spring bloom. *Marine Biology*, *138*(5), 1051–1062. Retrieved from https://scholar.google.com/scholar?hl=en&as_sdt=0%2C22&q=Phytoplankton+production+patterns+in+Massachusetts+Bay+and+the+absence+of+the+1998+winter%E2%80%93spring+bloom.&btnG=

Kenney, R. D., Winn, H. E., & Macaulay, M. C. (1995). Cetaceans in the Great South Channel,

1979-1989: right whale (*Eubalaena glacialis*). *Continental Shelf Research*, *15*, 385–414. https://doi.org/10.1016/0278-4343(94)00053-P

Klimova, A., Phillips, C. D., Fietz, K., Olsen, M. T., Harwood, J., Amos, W., & Hoffman, J. I. (2014). Global population structure and demographic history of the grey seal. *Molecular Ecology*, *23*(16), 3999–4017. https://doi.org/10.1111/mec.12850

Lawson, J., Magalhães, A., & Miller, E. (1998). Important prey species of marine vertebrate predators in the northwest Atlantic: proximate composition and energy density. *Marine Ecology Progress Series*, *164*, 13–20. https://doi.org/10.3354/meps164013

Leaf, R. T., & Friedland, K. D. (2014). Autumn bloom phenology and magnitude influence haddock recruitment on Georges Bank. *ICES Journal of Marine Science*, *71*(8), 2017–2025. https://doi.org/10.1093/icesjms/fsu076

Leeney, R. H., Stamieszkin, K., Mayo, C. A., & Marx, M. K. (2009). *Surveillance, monitoring, and management of North Atlantic right whales in Cape Cod Bay and adjacent waters-2009* (p. 89). Provincetown Center for Coastal Studies. Retrieved from https://www.mass.gov/files/documents/2016/08/qv/rwhale09.pdf

Lesage, V., & Hammill, M. O. (2001). The status of the grey seal, *Halichoerus grypus*, in the Northwest Atlantic. *Canadian Field-Naturalist*, *115*(4), 653–662. Retrieved from https://scholar.google.com/scholar?hl=en&as_sdt=0%2C22&q=The+status+of+the+grey+seal%2C+Halichoerus+grypus%2C+in+the+Northwest+Atlantic.&btnG=

Lesage, V., Hammill, M. O., & Kovacs, K. M. (2001). Marine mammals and the community structure of the Estuary and Gulf of St Lawrence, Canada: evidence from stable isotope analysis. *Marine Ecology Progress Series*, *210*, 203–221. Retrieved from http://www.jstor.org/stable/24863877

Loesch, J. (1987). Overview of Life History Aspects of Anadromous Alewife and Blueback Herring in Freshwater Habitats. *American Fisheries Society Symposium*. Retrieved from https://scholarworks.umass.edu/fishpassage_journal_articles/10

Logan, J. M., Golet, W. J., & Lutcavage, M. E. (2015). Diet and condition of Atlantic bluefin tuna (*Thunnus thynnus*) in the Gulf of Maine, 2004–2008. *Environmental Biology of Fishes*, *98*(5), 1411–1430. https://doi.org/10.1007/s10641-014-0368-y

Manning, C., & Bucklin, A. (2005). Multivariate analysis of the copepod community of near-shore waters in the western Gulf of Maine. *Marine Ecology Progress Series*, *292*, 233–

249. https://doi.org/10.3354/meps292233

Mayo, C. A., Ganley, L., Hudak, C. A., Brault, S., Marx, M. K., Burke, E., & Brown, M. W. (2018). Distribution, demography, and behavior of North Atlantic right whales (*Eubalaena glacialis*) in Cape Cod Bay, Massachusetts, 1998-2013. *Marine Mammal Science*, Advanced online publication. https://doi.org/10.1111/mms.12511

Melle, W., Runge, J., Head, E., Plourde, S., Castellani, C., Licandro, P., … Chust, G. (2014). The North Atlantic Ocean as habitat for *Calanus finmarchicus*: Environmental factors and life history traits. *Progress in Oceanography*, *129*, 244–284. https://doi.org/10.1016/j.pocean.2014.04.026

MERCINA. (2004). Supply-side ecology and the response of zooplankton to climate-driven changes in North Atlantic Ocean circulation. *Oceanography*, *17*(3), 60–71. https://doi.org/10.5670/oceanog.2004.31

Mills, K., Pershing, A. J., Brown, C., Chen, Y., Chiang, F.-S., Holland, D., … Wahle, R. (2013). Fisheries management in a changing climate: Lessons from the 2012 ocean heat wave in the Northwest Atlantic. *Oceanography*, *26*(2), 191–195. https://doi.org/10.5670/oceanog.2013.27

Murison, L. D., & Gaskin, D. E. (1989). The distribution of right whales and zooplankton in the Bay of Fundy, Canada. *Canadian Journal of Zoology*, *67*(6), 1411–1420. https://doi.org/10.1139/z89-200

Nisbet, I. C., Veit, R. R., Auer, S. A., & White, T. P. (2013). *Marine Birds of the Eastern United States and the Bay of Fundy*. Cambridge, Massachusetts: Harvard Univ Nuttall ornithological. Retrieved from http://www.buteobooks.com/product/NOM29.html

O'Reilly, J. E., & Zetlin, C. (1998). *Seasonal, horizontal, and vertical distribution of phytoplankton chlorophyll a in the Northeast U.S. Continental Shelf Ecosystem* (NOAA Technical Report, NMFS No. 139) (p. 119). U.S. Department of Commerce. Retrieved from http://spo.nwr.noaa.gov/tr139opt.pdf

Pendleton, D., Pershing, A., Brown, M., Mayo, C., Kenney, R., Record, N., & Cole, T. (2009). Regional-scale mean copepod concentration indicates relative abundance of North Atlantic right whales. *Marine Ecology Progress Series*, *378*, 211–225. https://doi.org/10.3354/meps07832

Pershing, A., J., Greene, C., Jossi, J., Obrien, L., Brodziak, J., & Bailey, B. (2005). Interdecadal

variability in the Gulf of Maine zooplankton community, with potential impacts on fish recruitment. *ICES Journal of Marine Science*, *62*(7), 1511–1523. https://doi.org/10.1016/j.icesjms.2005.04.025

Pettigrew, N. R., Churchill, J. H., Janzen, C. D., Mangum, L. J., Signell, R. P., Thomas, A. C., … Xue, H. (2005). The kinematic and hydrographic structure of the Gulf of Maine Coastal Current. *Deep Sea Research Part II: Topical Studies in Oceanography*, *52*(19–21), 2369–2391. https://doi.org/10.1016/j.dsr2.2005.06.033

Platt, T., Fuentes-Yaco, C., & Frank, K. T. (2003). Marine ecology: Spring algal bloom and larval fish survival. *Nature*, *423*(6938), 398–399. https://doi.org/10.1038/423398b

Platt, T., Sathyendranath, S., White, G. N., Fuentes-Yaco, C., Zhai, L., Devred, E., & Tang, C. (2010). Diagnostic properties of phytoplankton time series from remote sensing. *Estuaries and Coasts*, *33*(2), 428–439. https://doi.org/10.1007/s12237-009-9161-0

Pleizier, N. K., Campana, S. E., Schallert, R. J., Wilson, S. G., & Block, B. A. (2012). Atlantic Bluefin Tuna (*Thunnus thynnus*) Diet in the Gulf of St. Lawrence and on the Eastern Scotian Shelf. *Journal of Northwest Atlantic Fishery Science*, *44*, 67–76. https://doi.org/10.2960/J.v44.m685

Record, N. R., Pershing, A. J., & Jossi, J. W. (2010). Biodiversity as a dynamic variable in the Gulf of Maine continuous plankton recorder transect. *Journal of Plankton Research*, *32*(12), 1675–1684. https://doi.org/10.1093/plankt/fbq050

Richardson, D. E., Hare, J. A., Overholtz, W. J., & Johnson, D. L. (2010). Development of long-term larval indices for Atlantic herring (*Clupea harengus*) on the northeast US continental shelf. *ICES Journal of Marine Science*, *67*(4), 617–627. https://doi.org/10.1093/icesjms/fsp276

Rounsefell, G. A., & Stringer, L. D. (1945). Restoration and Management of the New England Alewife Fisheries with Special Reference to Maine. *Transactions of the American Fisheries Society*, *73*(1), 394–424. https://doi.org/10.1577/1548-8659(1943)73[394:RAMOTN]2.0.CO;2

Saunders, R., Hachey, M. A., & Fay, C. W. (2006). Maine's diadromous fish community: Past, present, and implications for Atlantic Salmon recovery. *Fisheries*, *31*(11), 537–547. https://doi.org/10.1577/1548-8446(2006)31[537:MDFC]2.0.CO;2

Sherman, K., & Schaner, E. G. (1968). Observations on the distribution and breeding of *Sagitta*

elegans (Chaetognatha) in coastal waters of the Gulf of Maine. *Limnology and Oceanography*, *13*(4), 618–625. https://doi.org/10.4319/lo.1968.13.4.0618

Shumway, S. E., Perkins, H. C., Schick, D. F., & Stickney, A. P. (1985). *Synopsis of biological data on the pink shrimp, Pandalus borealis Krøyer, 1838.* (NOAA Tech Report No. NMFS Series, 30) (p. 57). Retrieved from http://aquaticcommons.org/2793/

Siders, Z. A., Westgate, A. J., Johnston, D. W., Murison, L. D., & Koopman, H. N. (2013). Seasonal variation in the spatial distribution of basking sharks (*Cetorhinus maximus*) in the Lower Bay of Fundy, Canada. *PLoS ONE*, *8*(12), e82074. https://doi.org/10.1371/journal.pone.0082074

Townsend, D. W. (1983). The relations between larval fishes and zooplankton in two inshore areas of the Gulf of Maine. *Journal of Plankton Research*, *5*(2), 145–173. https://doi.org/10.1093/plankt/5.2.145

Townsend, D. W. (1984). Comparison of inshore zooplankton and ichthyoplankton populations of the Gulf of Maine. *Marine Ecology Progress Series. Oldendorf*, *15*(1), 79–90. http://www.int-res.com/articles/meps/15/m015p079.pdf

Trzcinski, M. K., Devred, E., Platt, T., & Sathyendranath, S. (2013). Variation in ocean colour may help predict cod and haddock recruitment. *Marine Ecology Progress Series*, *491*, 187–197. https://doi.org/10.3354/meps10451

Vermeer, K., Szabo, I., & Greisman, P. (1987). The relationship between plankton-feeding Bonaparte's and Mew Gulls and tidal upwelling at Active Pass, British Columbia. *Journal of Plankton Research*, *9*(3), 483–501. https://doi.org/10.1093/plankt/9.3.483

Waring, G. T., Gilbert, J. R., Belden, D., Van Atten, A., & DiGiovanni Jr, R. A. (2010). A review of the status of harbour seals (*Phoca vitulina*) in the Northeast United States of America. *NAMMCO Scientific Publications*, *8*, 191. https://doi.org/10.7557/3.2685

Waring, G. T., Josephson, E., Fairchild, C. P., & Maze-Foley, K. (2006). *U.S. Atlantic and Gulf of Mexico Marine Mammal Stock Assessments - 2005* (NOAA Technical Memorandum No. No. No. NMFS-NE-194) (pp. 163–168). U.S. Department of Commerce. Retrieved from https://www.nefsc.noaa.gov/publications/tm/tm194/pdfs/163.pdf

Wurtzell, K. V., Baukus, A., Brown, C. J., Jech, J. M., Pershing, A. J., & Sherwood, G. D. (2016). Industry-based acoustic survey of Atlantic herring distribution and spawning dynamics in coastal Maine waters. *Fisheries Research*, *178*, 71–81.

https://doi.org/10.1016/j.fishres.2015.11.011

Zhai, L., Platt, T., Tang, C., Sathyendranath, S., & Hernández Walls, R. (2011). Phytoplankton phenology on the Scotian Shelf. *ICES Journal of Marine Science*, *68*(4), 781–791. https://doi.org/10.1093/icesjms/fsq175
