# Supplementary material for: It’s about time: A synthesis of changing phenology in the Gulf of Maine ecosystem
Source: Fish Oceanogr. 2019 Apr 22;28(5):532–66. doi: 10.1111/fog.12429 (PMC6774335; doi:10.1111/fog.12429)
Supplement: Supplementary file 2 [file FOG-28-532-s002.pdf]

Appendix 2: A reanalysis of the Continuous Plankton Recorder to include the most recent data available for *Calanus* copepods (2001-2013).

With relatively large body size and high abundance, the calanoid copepod *Calanus finmarchicus* is a key trophic link between primary producers and upper trophic levels in the Gulf of Maine (GoM) food web (Payne et al., 1990). Its lifecycle is tuned to the annual cycles of primary production and predator communities, making its phenology critical to the population's persistence at the southern edge of its entire range, in the GoM. While several studies have examined annual cycles of *Calanus* production (e.g. Durbin et al., 1995; Conversi et al., 2001; Manning and Bucklin, 2005; Runge et al., 2015; Johnson et al., 2016), only two studies have evaluated how this cycle has changed in recent decades. One focused on Cape Cod Bay between 1999 and 2006, demonstrating that *C. finmarchicus* springtime phenology varied, with peak concentrations through March and April in 1999, 2000 and 2005, and a later peak (2-4 weeks lagged) in 2000 and 2003 compared to other years. In all years but 2002, this springtime peak declined in May (Pendleton et al., 2009). Pershing et al. (2005) estimated *Calanus* abundance in the GoM Continuous Plankton Recorder (CPR) time series during three historical periods between 1961 and 2000. We updated this analysis to include the most recent data (2001-2013), which also coincides with a rapid warming trend in the system (Pershing et al., 2015).

Calanus finmarchicus (CV-adult) and *Calanus* spp. (CI-CIV) concentrations (individuals m^{-3}) from the GoM CPR dataset (1961- May 2013) were analyzed for monthly averages, yielding a seasonal cycle for each year. Data from within the inner Gulf of Maine (excluding samples taken inshore of the 100 m isobath) were used to avoid confounding of the coastal and offshore systems (Tian et al., 2015), and were log-transformed due to the underlying log-normal

distribution of the data (Conversi *et al.*, 2001). After Pershing et al. (2005), the *Calanus* abundance seasonal cycles were averaged within three historical time periods (1961-1974, 1978-1988, 1992-2000), as well as the most recent (2001-2013), and all years combined. The three historical periods account for a data gap in the mid-1970s, and distinct periods in GoM oceanographic and zooplankton conditions (Pershing et al., 2005). Unlike Pershing et al. (2005), we elected not to use a spline smoothing function and anomaly analysis because our results were found to be consistent with the patterns presented by Pershing et al. (2005), and we preferred that results reflected measured organism concentrations.

This analysis showed that the average annual cycle of adult and late-stage (CV) *C. finmarchicus* has continued to reflect trends previously observed over the last several decades: their seasonal of production has broadened and increased in overall concentration compared to the earliest historical period in 1961-1974 (Figure 3A). The average annual cycle of juvenile *Calanus* spp. (CI-CIV) has also broadened and been elevated when comparing recent time periods (1978-2013) to the historical period of 1961-1974 (Figure 3B).

References

Conversi, A., Piontkovski, S., & Hameed, S. (2001). Seasonal and interannual dynamics of *Calanus finmarchicus* in the Gulf of Maine (Northeastern US shelf) with reference to the North Atlantic Oscillation. *Deep Sea Research Part II: Topical Studies in Oceanography*, *48*(1–3), 519–530. https://doi.org/10.1016/S0967-0645(00)00088-6

Durbin, E. G., Gilman, S. L., Campbell, R. G., & Durbin, A. G. (1995). Abundance, biomass, vertical migration and estimated development rate of the copepod *Calanus finmarchicus* in the southern Gulf of Maine during late spring. *Continental Shelf Research*, *15*(4), 571–

591. https://doi.org/10.1016/0278-4343(94)00060-Z

Johnson, C., Casault, B., Head, E., & Spry, J. (2016). *Optical, Chemical, and Biological Oceanographic Conditions on the Scotian Shelf and in the Eastern Gulf of Maine in 2014.* (DFO Can. Sci. Advis. Sec. Res. Doc. No. 2016/003) (p. *v* +51). Retrieved from http://waves-vagues.dfo-mpo.gc.ca/Library/362284.pdf

Manning, C., & Bucklin, A. (2005). Multivariate analysis of the copepod community of near-shore waters in the western Gulf of Maine. *Marine Ecology Progress Series*, *292*, 233–249. https://doi.org/10.3354/meps292233

Payne, P. M., Wiley, D. N., Pittman, S., Clapham, P. J., & Jossi, J. W. (1990). Recent fluctuations in the abundance of baleen whales in the southern Gulf of Maine in relation to changes in selected prey. *Fishery Bulletin*, *88*, 687–696. http://fishbull.noaa.gov/884/payne.pdf

Pershing, A. J., Greene, C., Jossi, J., Obrien, L., Brodziak, J., & Bailey, B. (2005). Interdecadal variability in the Gulf of Maine zooplankton community, with potential impacts on fish recruitment. *ICES Journal of Marine Science*, *62*(7), 1511–1523. https://doi.org/10.1016/j.icesjms.2005.04.025

Pershing, A. J., Alexander, M. A., Hernandez, C. M., Kerr, L. A., Bris, A. L., Mills, K. E., … Thomas, A. C. (2015). Slow adaptation in the face of rapid warming leads to collapse of the Gulf of Maine cod fishery. *Science*, *350*(6262), 809–812. https://doi.org/10.1126/science.aac9819

Runge, J. A., Ji, R., Thompson, C. R. S., Record, N. R., Chen, C., Vandemark, D. C., … Maps, F. (2015). Persistence of *Calanus finmarchicus* in the western Gulf of Maine during recent extreme warming. *Journal of Plankton Research*, *37*(1), 221–232.

https://doi.org/10.1093/plankt/fbu098

Tian, R., Chen, C., Qi, J., Ji, R., Beardsley, R. C., & Davis, C. (2015). Model study of nutrient and phytoplankton dynamics in the Gulf of Maine: patterns and drivers for seasonal and interannual variability. *ICES Journal of Marine Science*, *72*(2), 388–402. https://doi.org/10.1093/icesjms/fsu090
