# Supplementary material for: It’s about time: A synthesis of changing phenology in the Gulf of Maine ecosystem
Source: Fish Oceanogr. 2019 Apr 22;28(5):532–66. doi: 10.1111/fog.12429 (PMC6774335; doi:10.1111/fog.12429)
Supplement: Supplementary file 3 [file FOG-28-532-s003.pdf]

Appendix 3: Detailed description and results of a literature search conducted in ISI Web of Knowledge for phenology studies in the Gulf of Maine spanning 1960-2017.

A review of the literature was conducted using ISI Web of Knowledge using the joint term “phenology and Northwest Atlantic”, followed by searches of the major basins in the Gulf of Maine (i.e. [phenology and "name of sub-region"]) within the search field "Topic" for the time period of 1960-2018. Sub-regions searched included “Gulf of Maine”, “Scotian shelf”, “Bay of Fundy”, “Brown Banks”, “Georges Bank”, “Crowell Basin”, “Cashes Ledge”, “Halifax”, “Northeast Channel”, “New Scantum”, “Jeffreys Ledge”, “Nantucket Shoals”, “Prince”, and “Wilkinson Basin”. A total of twenty-seven papers were found that focused on coastal and marine organisms in the Gulf of Maine region and each study was reviewed to determine if analyses or results evaluated shifts in life history events over time. Only four studies were identified meeting this criteria (Richards, 2012; Lambert 2013; Friedland et al., 2015; Thomas et al., 2017). Two additional studies projected phenological shifts under future scenarios of climate change either using temperature increases or climate scenarios (Pierson et al., 2013; Wilson et al., 2016). The remainder investigated basic information on phenological events were classified as background or baseline studies and not counted as documented shifts in phenology. This included studies that documented phenological cycles and established seasonal patterns (Kaczmarska and Dowe, 1997; Bouchard et al., 2012) or evaluated the relationship between a climate variable and phenological event (e.g., Gilbert et al., 2010; Davies et al., 2014). Several studies developed models that may be used to advance understanding of species-specific and ecosystem-level responses to climate change including shifts in timing, but for their application

the authors did not directly address phenology in their analyses (Link et al., 2010; Neuheimer et al., 2010; Kleisner et al., 2016).

References

Bouchard, G. M., & Aiken, R. B. (2012). Latitudinal variation in the reproductive cycle and size of the northern rock barnacle *Semibalanus balanoides* (L.) (Cirripedia, Archaeobalanidae) in the Bay of Fundy. *Crustaceana*, *85*(7), 779–787. https://doi.org/10.1163/156854012X650214

Davies, K., Gentleman, W., DiBacco, C., & Johnson, C. (2014). Semi-annual spawning in marine scallops strengthens larval recruitment and connectivity on Georges Bank: a model study. *Marine Ecology Progress Series*, *516*, 209–227. https://doi.org/10.3354/meps10975

Friedland, K. D., Leaf, R. T., Kane, J., Tommasi, D., Asch, R. G., Rebuck, N., … Saba, V. S. (2015). Spring bloom dynamics and zooplankton biomass response on the US Northeast Continental Shelf. *Continental Shelf Research*, *102*, 47–61. https://doi.org/10.1016/j.csr.2015.04.005

Gilbert, C. S., Gentleman, W. C., Johnson, C. L., DiBacco, C., Pringle, J. M., & Chen, C. (2010). Modelling dispersal of sea scallop (*Placopecten magellanicus*) larvae on Georges Bank: The influence of depth-distribution, planktonic duration and spawning seasonality. *Progress in Oceanography*, *87*(1–4), 37–48. https://doi.org/10.1016/j.pocean.2010.09.021

Kaczmarska, I., & Dowe, L. L. (1997). Reproductive biology of the red alga Polysiphonia lanosa (Ceramiales) in the Bay of Fundy, Canada. *Marine Biology*, *128*(4), 695–703. https://doi.org/10.1007/s002270050137

Kleisner, K. M., Fogarty, M. J., McGee, S., Barnett, A., Fratantoni, P., Greene, J., … Pinsky, M. L. (2016). The Effects of Sub-Regional Climate Velocity on the Distribution and Spatial

Extent of Marine Species Assemblages. *PLOS ONE*, *11*(2), e0149220. https://doi.org/10.1371/journal.pone.0149220

Lambert, W. J. (2013). Population Biology of an Intertidal Dorid Nudibranch (*Onchidoris muricata*) in the Southern Gulf of Maine, U.S.A.: Changes in Phenology Due to an Invasive Prey? *American Malacological Bulletin*, *31*(1), 17–23. https://doi.org/10.4003/006.031.0109

Link, J. S., Fulton, E. A., & Gamble, R. J. (2010). The northeast US application of ATLANTIS: A full system model exploring marine ecosystem dynamics in a living marine resource management context. *Progress in Oceanography*, *87*(1–4), 214–234. https://doi.org/10.1016/j.pocean.2010.09.020

Neuheimer, A. B., Gentleman, W. C., Pepin, P., & Head, E. J. H. (2010). How to build and use individual-based models (IBMs) as hypothesis testing tools. *Journal of Marine Systems*, *81*(1–2), 122–133. https://doi.org/10.1016/j.jmarsys.2009.12.009

Pierson, J. J., Batchelder, H., Saumweber, W., Leising, A., & Runge, J. (2013). The impact of increasing temperatures on dormancy duration in Calanus finmarchicus. *Journal of Plankton Research*, *35*(3), 504–512. https://doi.org/10.1093/plankt/fbt022

Richards, R. A. (2012). Phenological shifts in hatch timing of northern shrimp *Pandalus borealis*. *Marine Ecology Progress Series*, *456*, 149–158. https://doi.org/10.3354/meps09717

Thomas, A. C., Pershing, A. J., Friedland, K. D., Nye, J. A., Mills, K. E., Alexander, M. A., … Henderson, M. E. (2017). Seasonal trends and phenology shifts in sea surface temperature on the North American northeastern continental shelf. Elem Sci Anth, 5(0), 48. https://doi.org/10.1525/elementa.240

Wilson, R. J., Banas, N. S., Heath, M. R., & Speirs, D. C. (2016). Projected impacts of 21st century climate change on diapause in *Calanus finmarchicus*. *Global Change Biology*, *22*(10), 3332–3340. https://doi.org/10.1111/gcb.13282

Appendix 3: Results of ISI Web of Knowledge literature search using the joint term "marine and phenology". The 27 results are organized by functional group and give a brief description of the type of phenology data each study contains. * indicates a study that documented a shift in phenology and **indicates a study that projected phenological shifts under future scenarios of climate change.

**Functional Group**	**Species**	**Time period**	**Region**	**Phenological shift**	**Observation type**	**Reference**
Abiotic variables		1982-2014	GoM, Georges Bank, Northeast Shelf	Spring and fall transition dates	Daily, optimally interpolated NOAA SST data (OISST)	Thomas et al., 2017
Phytoplankton		n/a	Scotian Shelf and Gulf of Maine	Established relationship between earlier bloom timing and freshening / salinity	Biological–physical coupled model system using ocean-colour and satellite observations (SeaWiFS)	Ji et al., 2008
Phytoplankton		1998–2007	Northwest Atlantic Ocean	Baseline study of how phytoplankton blooms vary interannually and with latitude	Phytoplankton–substrate model developed using SeaWiFS and NASA Giovanni	Platt et al., 2009
Phytoplankton		1998-2008	Nova Scotian Shelf, Gulf of Maine	Spring bloom timing is positively correlated with sea surface salinity (SSS); fall bloom timing is negatively correlated with SSS and SST	Modeling study using SeaWiFS	Song et al., 2010
Phytoplankton		1998-2007; winter-spring	Scotian Shelf and Scotian Slope	Shoaling of the mixed-layer depth was related to early spring bloom; bloom amplitutde correlated with the nitrate in winter surface waters	Modeling study using SeaWiFS	Zhai et al., 2011
Marine plants & algae	*Porphyra yezoensis*	1996, 1999	New Brunswick, Canada; Cobscook Bay, ME; East Ross Island	Baseline study to understand production for net seeding and growth conditions in aquaculture	Field and laboratory studies	Chopin et al., 1999
Marine plants & algae	Red alga (*Polysiphonia lanosa*)	1990-1992	Bay of Fundy	Baseline study on reproductive biology	Field collections	Kaczmarska and Dowe, 1997
Zooplankton**	*Calanus finmarchicus*	n/a	Gulf of Maine	Projected reduction in overwintering diapause duration of ~39 days with a 2°C temperature increase	Modeling study using temperature projections relative to empirical measurements of biological observations	Pierson et al., 2013

Functional Group	Species	Time period	Region	Phenological shift	Observation type	Reference
Zooplankton**	*Calanus finmarchicus*	2000-2009; 2090-2099	Northwest and Northeast Atlantic	Projected reduction in overwintering diapause duration of ≥ 50%	Modeling study using temperature projections and field collections of biological observations	Wilson et al., 2016
Zooplankton	*Calanus finmarchicus*	1999-2000	Gulf of St. Lawrence	Baseline study providing a mechanistic understanding of factors controling dormancy duration and lipid content	Modeling study	Maps et al. 2010
Zooplankton	*Calanus finmarchicus*	n/a	Gulf of St. Lawrence and Gulf of Maine	Baseline study to establish the seasonal cycle of dormancy and energy content	Modeling study	Maps et al. 2012
Zooplankton	*Calanus finmarchicus*	1999-2007	Nova Scotia, Canada	Develped a model to aid in studies of recruitment, abundance, timing and environmental influences	Individual-based models (IBMs)	Neuheimer et al., 2010
Zooplankton*		1998-2013	Northeast Shelf Ecosystem	Showed shift towards earlier spring transition dates and later fall transition dates	Modeling study using SeaWiFS and Moderate Resolution Imaging Spectroradiometer (MODIS) sensors	Friedland et al., 2015a
Multispecies	Phytoplankton and zooplankton	2005-2008	Gulf of Maine	Baseline stdy investigating causal relationships between leading and lagging (sequence and sucession) relationships for primary and secondary production	Modeling study	Seip, 2015
Macro-invertebrates	Sea scallop (*Placopecten magellanicus*)	n/a	Georges Bank	Baseline study on transport, recruitment, and distribution	3D particle-tracking mode	Gilbert et al., 2010
Macro-invertebrates	Sea scallop (*Placopecten magellanicus*)	1984-2004	Georges Bank	Baseline study to understand larval production and connectivity over spatio-temporal scales	Modeling study	Davies et al., 2014
Macro-invertebrates	Intertidal amphipod, (*Corophium volutator*)	1997	Bay of Fundy	Baseline study of seasonal mate limitation	Field survey	Forbes et al., 2006
Macro-invertebrates*	Intertidal Dorid Nudibranch (*Onchidoris muricat*)	2005-2009	Newcastle, NH	Spawning extended into summer with expansion of recruitment in early autum. Peak spawning shifted from January-March to May/June	Biological census of intertidal habitats and populations	Lambert, 2013
Macro-invertebrates	Northern rock barnacle (*Semibalanus balanoides*)	2006-2007	Bay of Fundy	Baseline study of the latitudinal variation in reproductive phenology	Field collections	Bouchard and Aiken, 2012

Functional Group	Species	Time period	Region	Phenological shift	Observation type	Reference
Macro-invertebrates*	Northern shrimp (*Pandalus borealis*)	1980-1983; 1989-2011	Gulf of Maine	Showed earlier hatch initiation, later completion, and overall longer duration of hatching period	Commercial fishery catch data	Richards, 2012
Macro-invertebrates	Northern shrimp (*Pandalus borealis*)	1998-2012	Cape Ann, MA - Penobscot Bay, ME	Established relationship between SST and larval survival around summer emergence	Modeling study using SeaWiFS	Richards et al., 2016
Fish	Atlantic herring (*Clupea harengus*)	2012	Coastal Maine	Baseline monitoring study to establish spatio-temporal habitat use	Industry-based acoustic survey	Wurtzell et al., 2016
Fish	Haddock (*Melanogrammus aeglefinus*)	1960–2010	Georges Bank	Establishes relationship between autumn bloom timing and haddock recruitment strength	Modeling study using SeaWiFS, MODIS and NMFS population assessments	Leaf and Friedland, 2014
Seabirds	Common tern (*Sterna hirundo*)	1991-2002	Eastern Egg Rock, Seal, Jenny and Stratton Islands	Compares phenology metrics among islands and in relationship to population size	Field monitoring	Hall and Kress, 2004
Multispecies	45 functional groups including plants, fish, invertebrates, marine mammals and seabirds	1964-2014	Northeast Shelf Ecosystem (Gulf of Maine to Cape Hatteras)	Developed an ecosystem model to help understand multispecies dynamics	Modeling study	Link et al., 2010
Multispecies	8 assemblages of demersal, pelagic and reef-associated finfish and elasmobranchs	1968–2012	Gulf of Maine, Georges Bank, Mid-Atlantic Bight	Examined climate-induced shifts in biomass distribution relative to shifts in temperature	Modeling study using NEFSC trawl survey data	Kleisner et al., 2016
Multispecies	Alewives (*Alosa pseudoharengus*), American shad (*Alosa sapidissima*) and salmon (*Salmo salar*), Atlantic herring (*Clupea harengus*), and American mackerel (*Scomber scombru* s) and menhaden (*Brevoortia tyrannus*)	1804-1880	Gulf of Maine	Historical analysis of system response after the extreme climate event caused by the eruption of the volcano Tambora in 1815	Complex adaptive systems theory and historical fish export data	Alexander et al., 2017
