# Supplementary material for: It’s about time: A synthesis of changing phenology in the Gulf of Maine ecosystem
Source: Fish Oceanogr. 2019 Apr 22;28(5):532–66. doi: 10.1111/fog.12429 (PMC6774335; doi:10.1111/fog.12429)
Supplement: Supplementary file 4 [file FOG-28-532-s004.pdf]

Appendix 4: Examples of long-term monitoring programs in the Gulf of Maine that have potential timeseries to evaluate regional phenology-related inqueries.

Organization	Monitoring Program Name	Monitoring_objective	Years of program	Parameters Measured	Habitat and location	State/Province	Latitude	Longitude	Collection frequency	Data collection equipment	Waterbody secondary	Website
University of Maine	Gulf of Maine NERACOOS buoys	Operate and maintain a system of high-tech-buoys to collect near real-time ocean and weather data. Currently, the system consists of 6 buoys from offshore to inshore, including Penobscot Bay to Massachusetts Bay.	2000 - present (depending on buoy)	Wind speed, Wind direction, Wind gust, Wave height, Dominant wave period, Air temperature, Visibility, Atmospheric pressure, Water temperature, Current direction, Current speed, Salinity, Density, Turbidity, Chlorophyll concentration, Dissolved oxygen, Oxygen saturation, Percent oxygen saturation	Pelagic, coastal	ME, MA	various	various	Hourly	Sondes, pressure sensor, water quality probes	Gulf of Maine	http://gyre.umeoce.maine.edu/ http://www.neracoos.org/realtime_map
NOAA Northeast Fisheries Science Center	Ecosystem Monitoring (EcoMon) survey	Assess the pelagic components of the Northeast U.S. Continental Shelf Ecosystem.	1970 - present	Zooplankton, ichthyoplankton, water column	Pelagic	GoM	multiple locations	multiple locations	Seasonal	CTD, bongo net, acoustics using EK60		https://www.nefsc.noaa.gov/epd/ocean/MainPage/shelfwide.html
	Continuous Plankton Recorder survey		1961-2013	Zooplankton, phytoplankton	Pelagic	GoM	multiple locations	multiple locations	Bi-monthly			
	Environmental Monitors on Lobster Traps (eMOLT)	To provide long term bottom temperature time series for the Gulf of Maine and Southern New England Shelf.	2001 - present	Bottom temperature	bottom water	MA and ME	multiple locations	multiple locations	Hourly	VEMCO minilogs and Aquatec loggers	Gulf of Maine	https://www.nefsc.noaa.gov/epd/ocean/MainPage/lob/index.html
Canadian Department of Fisheries and Oceans	Atlantic Zonal Monitoring Program	To provide biological, chemical and physical data in support of characterizing and understanding causes of seasonal, annual and decadal oceanic variability and development of sound ocean activities	1999-present	Multiple oceanographic, chemical, and biological time series	Pelagic	NB, NS	44.930 and multiple locations	-66.850 and multiple locations	Monthly at Prince-5 and seasonal surveys			http://www.meds-sdmm.dfo-mpo.gc.ca/isdm-gdsi/azmp-pzma/index-eng.html
National Estuarine Research Reserve System Monitoring Program	Great Bay NERR, Wells NERR	To identify and track short-term variability and long-term changes in the integrity and biodiversity of estuarine ecosystems.	1997 or 2002 - present (depending on station)	Water and air temperatures, water depth, tides, salinity, conductivity, pH, turbidity, DO, TSS, Chlorophyll-a, Phaeopigments, Ammonium, Nitrite + Nitrate, Orthophosphate, TDN, PON, POC, Silica, PAR, atmospheric pressure, relative humidity, precipitation; wind speed, direction and gusts	Riverine, estuarine, pelagic	NH and ME	multiple locations	multiple locations	Continuous; grab samples for nutrients are monthly; diel water quality are monthly 24-h	Buoy; pressure sensor, water quality probes, sensors, sondes, meterological instrumentation; YSI 6600 and EXO dataloggers, Campbell Scientific MET Station, grab sampling	Great Bay, Lamprey River, Oyster River, Squamscott River, Webhannet River	http://cdmo.baruch.sc.edu/ http://www.wellsreserve.org/ http://greatbay.org/programs/monitoring.htm
Stellwagen Bank National Marine Sanctuary (SBNMS)	Right Whale Listening Buoys; Ship Strikes to Baleen Whales; Soundscape (Passive acoustic monitoring)	To alert commercial shippers to the presence of North Atlantic right whales; monitor the number of baleen whales reported from within and around the sanctuary; assess ocean background noise at different frequencies in SBNMS, characterize the noise, and understand the acoustic environment experienced by, as well as created by, vocally-active animals in the sanctuary.	1996-1999, 2002, 2004, 2006, 2007-2010, 2012-present	Right Whale sounds; baleen whale occurrence and low-frequency underwater sound (10 to 1000 Hz)	Pelagic, water column	MA	42.76639	-70.21694	Continuous	Buoys; Automatic Identification System (AIS); Visual surveys; Marine Autonomous Recording Units (MARUs)	NE Atlantic Cape Cod Bay	http://www.listenforwhales.org/Page.aspx?pid=430
Maine Department of Marine Resources (ME DMR)	Boothbay Harbor (BBH) Sea Water Temperature Record	To provide a long-term, continuous series of sea temperature observations for the U.S. Atlantic Coast.	1905 - present	Water and air temperatures, relative humidity, barometric pressure, wind speed/direction, tide height	Pelagic, embayment, water column	ME	43.843873	-69.640237	Daily	electronic sensors	Boothbay Harbor	http://www.maine.gov/dmr/science-research/weather-tides/index.html#
	Lobster Sea Sampling	Collect catch, effort, and biological data on harvested and discarded lobsters	1985-present	Lobster carapace length, sex, cull status, v-notch condition, egg development stage, molt status, presence or extent of shell disease	Benthic	ME	multiple locations	multiple locations	3 trips per month per lobster management zone	sea sampler	Gulf of Maine	http://www.maine.gov/dmr/science-research/species/lobster/seasampling.html
	Marine Biotoxin Monitoring Program	Monitoring biotoxins in shellfish in coastal Maine	2014-present	Domoic acid, saxitoxin, other compounds	Coastal	ME	multiple locations	multiple locations	Weekly			https://www.maine.gov/dmr/shellfish-sanitation-management/programs/biotoxinmonitoring.html
Marine & Environmental Research Institute (MERI)	Phytoplankton Red tide Monitoring	To conduct red tide monitoring in Blue Hill Bay in partnership with the Maine Department of Marine Resources (DMR).	2004 - 2013	Water quality- pH, temperature, salinity, conductivity, chlorophyl-a, dissolved oxygen, turbidity; weather conditions, tide phase	Pelagic	ME	44.407774	-68.578412	Weekly (April-October)	YSI 6600 V2 DataSonde	Blue Hill Bay	http://www.meriresearch.org/coastalmonitoring

Appendix 4: Examples of long-term monitoring programs in the Gulf of Maine that have potential timeseries to evaluate regional phenology-related inqueries.

Organization	Monitoring Program Name	Monitoring_objective	Years of program	Parameters Measured	Habitat and location	State/Province	Latitude	Longitude	Collection frequency	Data collection equipment	Waterbody secondary	Website
New Hampshire Department of Environmental Services and Piscataqua Region Estuary Partnership	Great Bay Estuary Water Quality Monitoring Program	To Provide a Nearly Continuous Record of Physical and Chemical Water Quality in Great Bay and its Tributaries.	2003 - present	Salinity, water Level, conductivity, temperature, pH, turbidity, and dissolved Oxygen at 10 stations and grab samples for nutrients, DOC, POC, silica, bacteria, dissolved oxygen, chlorophyll-a, phaeophytin and TSS at 11 stations	Riverine and Estuary	NH	43.111	-70.862	Continuous data sondes (30 minute intervals May to December); grab samples biweekly	Sondes, pressure sensor, water quality probes	Great Bay tributaries	https://www.des.nh.gov/organization/divisions/water/wmb/coastal/great-bay-estuary.htm
Bigelow Laboratory for Ocean Sciences	Booth Bay Plankton Monitoring (Dock Study)	To monitor long-term phytoplankton distributions and population dynamics.	2000 - present	Temperature, salinity, phytoplankton, bacteria, and eukaryotic heterotrophs (composition and abundance), chl-a	Pelagic, water column	ME	43.859782	-69.580268	Weekly	Flow cytometry	Booth Bay	https://www.st.nmfs.noaa.gov/copepod/time-series/us-10401/ ; https://seabass.gsfc.nasa.gov/experiment/GNATS/
	Gulf of Maine North Atlantic Time Series (GNATS)	Coordinated ship/satellite monitoring to measure carbon production and temporal variation. .	1998 - present	Temperature, salinity, water chemistry, carbon-specific standing stocks and rate measurements; hydrographic, chemical and optical measurements	Pelagic	ME-Nova Scotia	multiple locations	multiple locations	Monthly			
	Maine Coastal Time Series Station	Oceanographic monitoring and education	2006-present	CTD, chlorophyll, zooplankton counts	Coastal	ME	43.7448	-69.5043	monthly	CTD, ring net		
Northeastern Regional Association of Coastal Ocean Observing Systems (NERACOOS)	NERACOOS System	Buoys and stations collecting real-time weather and ocean data	2005 - present (depending on station)	Water and air temperatures, salinity, turbidity, DO, Chlorophyll-a, PAR, wind speed, direction and gusts, atmospheric pressure, pCO2 in air and water, alkalinity, pH	Pelagic	GoM	multiple locations	multiple locations	Hourly	Sondes, pressure sensor, water quality probes	Gulf of Maine; Piscataqua River, NH; Great Bay, NH	http://www.neracoos.org/
Provincetown Center for Coastal Studies	Right Whale Program	To monitor right whales and their food resource	1984 - present	Marine mammal, plankton, and other biological monitoring; oceanography monitoring	Pelagic	MA and ME	multiple locations	multiple locations	Weekly (winter, spring)	CTD with PAR sensor & fluorometer, conical net	Cape Cod Bay	http://coastalstudies.org/
	Cape Cod Bay Water Quality Program	To monitor water quality in Cape Cod Bay and surrounding waters	2006 - present	Water quality and oceanography monitoring	Pelagic, coastal	MA	multiple locations	multiple locations	Monthly	CTD with DO sensor, PAR sensor, & fluorometer, Niskin bottle, YSI Pro Plus & Pro DSS, secci disk	Cape Cod Bay	
Audubon	Project Puffin	To advance science, conservation, and protection of seabirds	1978-present	Seabird provisioning diets, nesting and fledging phenology; population assessments	Coastal	ME	multiple locations	multiple locations	Daily	Visual observations; weather station		http://projectpuffin.audubon.org/
Damariscotta River Association	Damariscotta River Monitoring	To monitor water quality, shellfish and other habitat, using citizen volunteers, high school students, and professional aquaculturists who look to the river for their livelihood.	1988 - present	Water quality information including DO, nitrogen, pH, temperature, salinity	Estuarine: 7 locations from Great Salt Bay to Walpole	ME	44.0517708	-69.513727	Bi-weekly	YSI 85, TN sample bottles	Damariscotta River, ME	http://www.damariscottariver.org/
Friends of Casco Bay	Monitoring By Sea	Monitoring the health of Casco Bay		Temperature, salinity, DO, pH, chlorophyll, depth, partial pressure of carbon dioxide, color, alkalinity, DIC, calcium carbonate saturation state, total nitrogen, dissolved inorganic nitrogen (nitrate and nitrite, ammonium, silicate, phosphate)	Pelagic, coastal	ME	multiple locations	multiple locations	Daily to monthly, depending on station	Date sonde, carbon dioxide sensor		https://www.cascobay.org/our-work/science/
Boothbay Region Land Trust	Water Sampling	Monitoring ocean health	2015-present	Temperature, salinity, DO, pH, secchi depth	Pelagic, coastal	ME	multiple locations	multiple locations	Bi-weekly	pH meter, salinity meter, secchi disk		http://www.bbrlt.org/water-samplers/
